# Supplementary material for: SpatialSNV: A novel method for identifying and analyzing spatially resolved SNVs in tumor microenvironments
Source: Gigascience. 2025 Jun 14;14:giaf065. doi: 10.1093/gigascience/giaf065 (PMC12166308; doi:10.1093/gigascience/giaf065)
Supplement: giaf065_Supplementary_Files_r3 [file giaf065_supplementary_files_r3.docx]

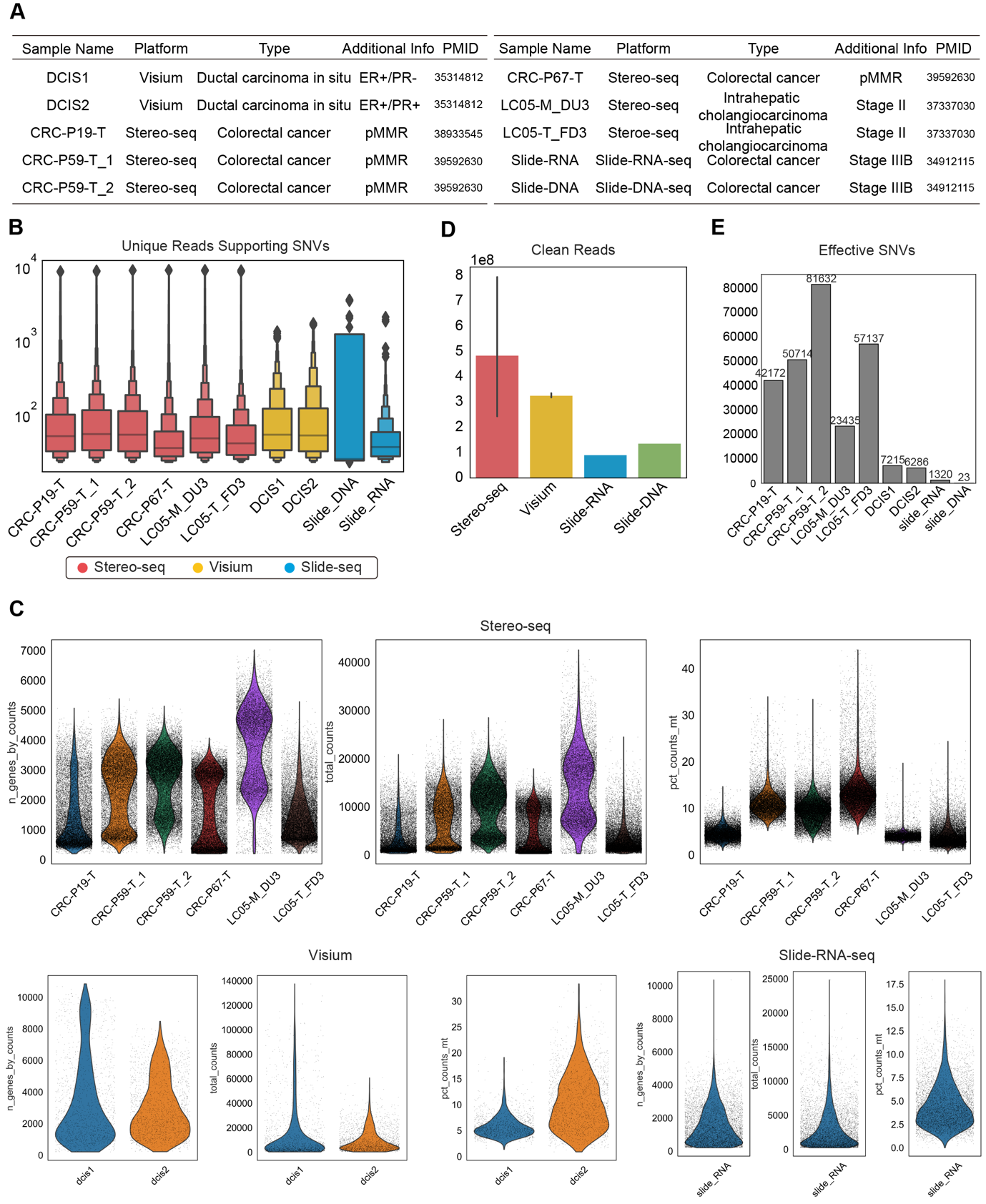


Supplementary Figure 1. The Quality Control of Spatial RNA and SNV Data Across Various Sections

**(A)** Table presenting the data sources for each sample. **(B)** Bar plot displaying the number of unique reads supporting SNVs for each section. **(C)** Violin plot illustrating the distribution of basic quality control metrics for spatial transcriptomics data across all sections, including the number of genes by counts (n_gene_by_counts), total counts, and percentage of mitochondrial counts (pct_count_mt). **(D)** Bar plot showing the quantity of clean reads across different platforms, with error bars indicating variability among sections. **(E)** Bar plot depicting the number of effective reads for all section.


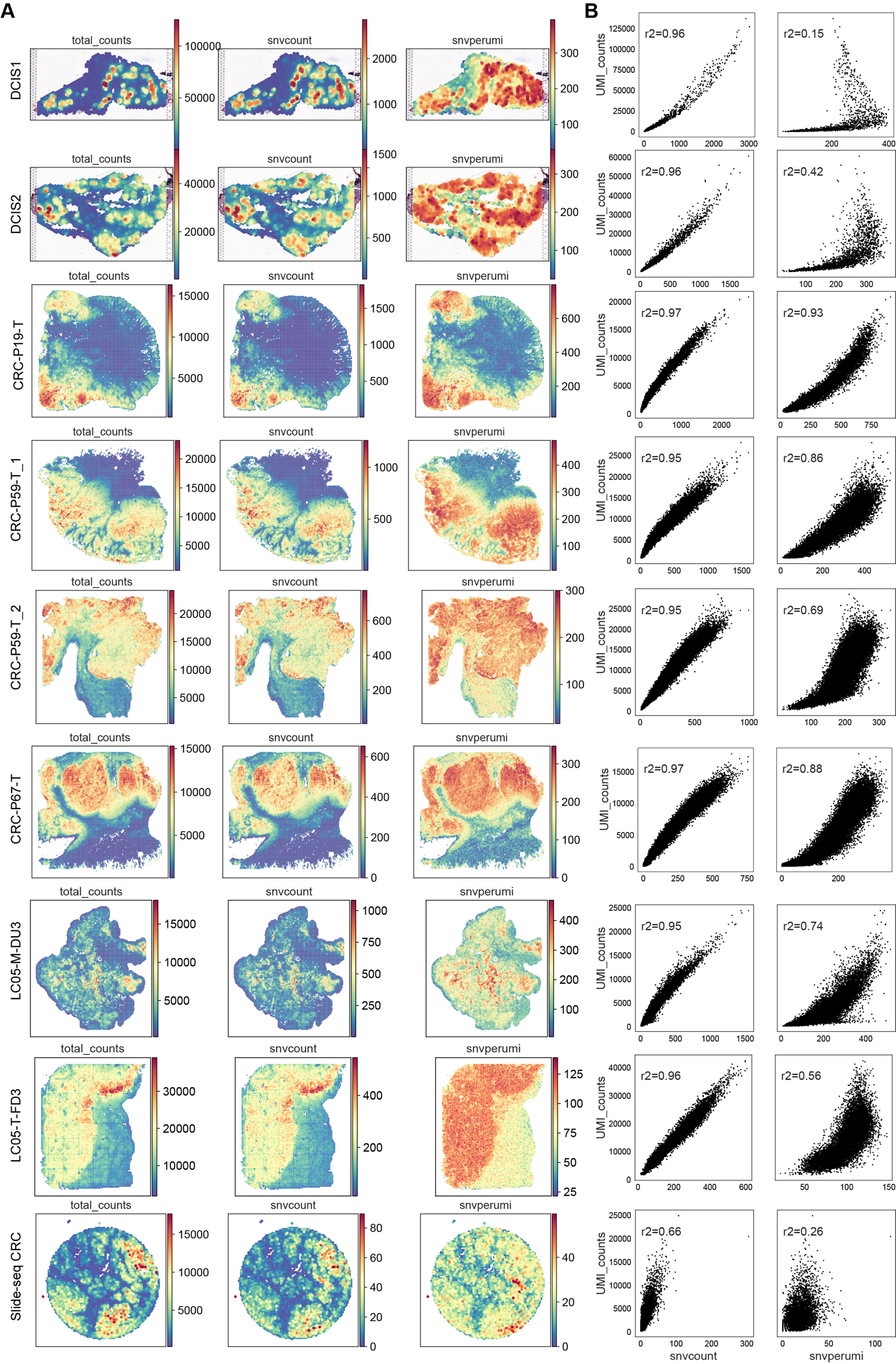


Supplementary Figure 2. Relationship Between Spatial Transcriptomics and Spatial SNVs

**(A)** Spatial visualization illustrating the total RNA UMI counts(left), SNV counts(mid), and normalized SNV per UMI (Normalized SNV Count)(right). **(B)** Scatter plots correlating SNV count and normalized SNV count with UMI count per spot, including the Pearson correlation coefficient (R²).


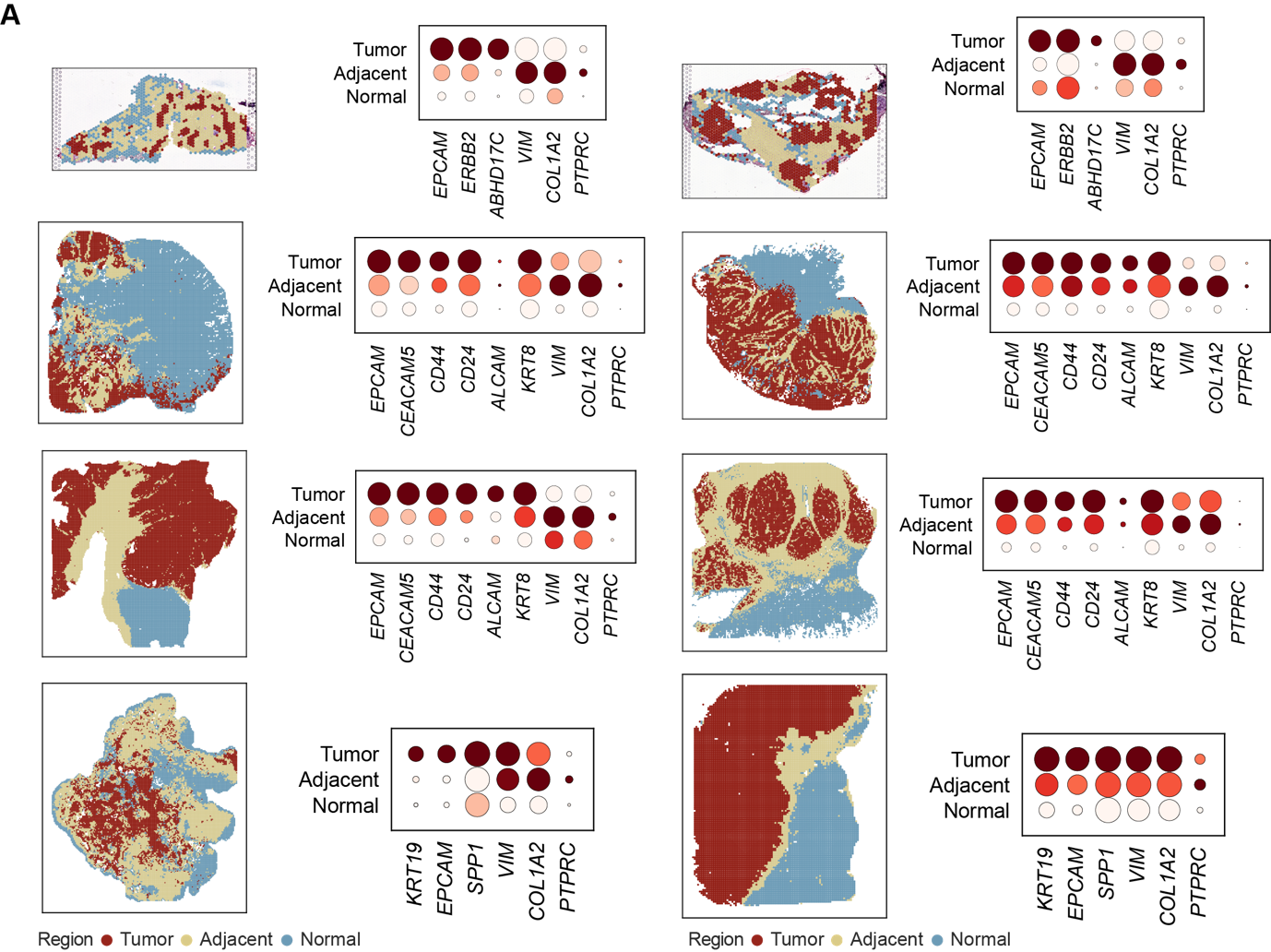


Supplementary Figure 3. The Definition of spatial region

**(A)** Spatial visualization of region clustered by spatial transcriptomics and the dot plot showing the main marker of each cluster region.


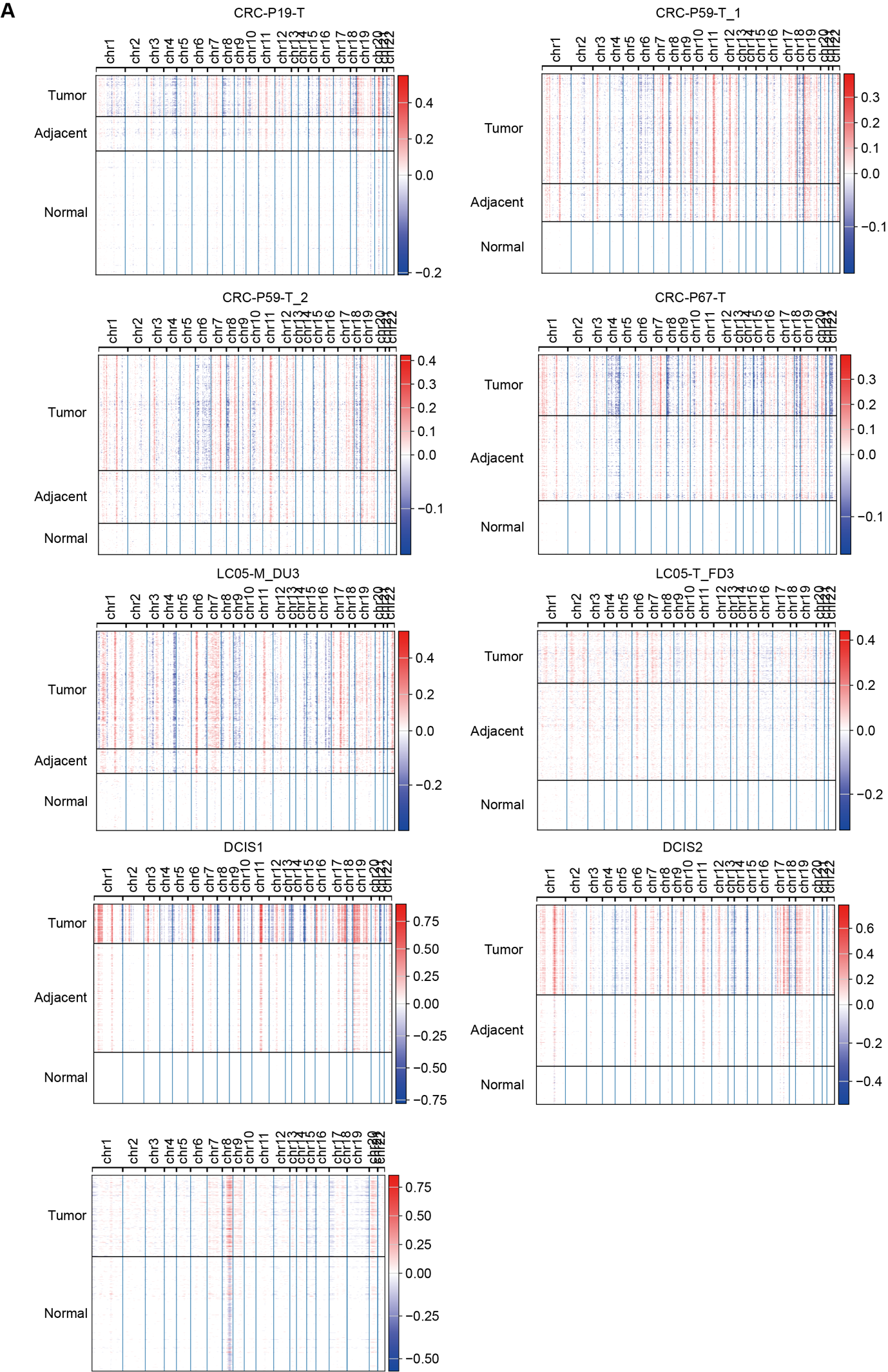


Supplementary Figure 4. The inferCNV results of each section

**(A)** Heatmap generated by inferCNV displaying inferred CNA profiles for each region clustered by spatial transcriptomics.


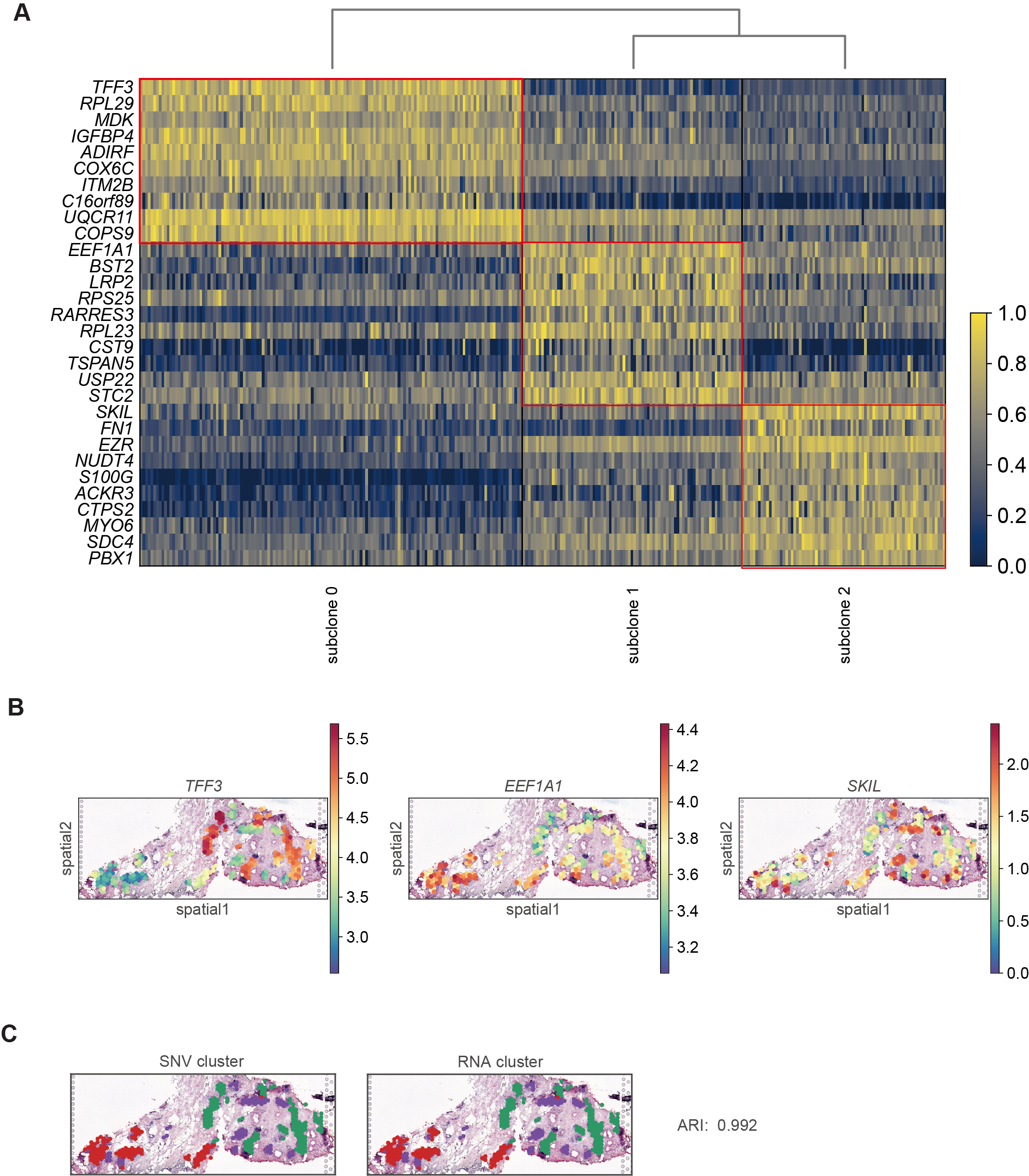


Supplementary Figure 5. Transcriptional Characteristics of Tumor Subclones in the DCIS1 Section.

**(A)** Heatmap of the top 10 differential genes of three subclones. **(B)** Spatial visualization of main differential genes of three subclones. **(C)** Spatial visualization of SNV and RNA clustering for tumor subclones.


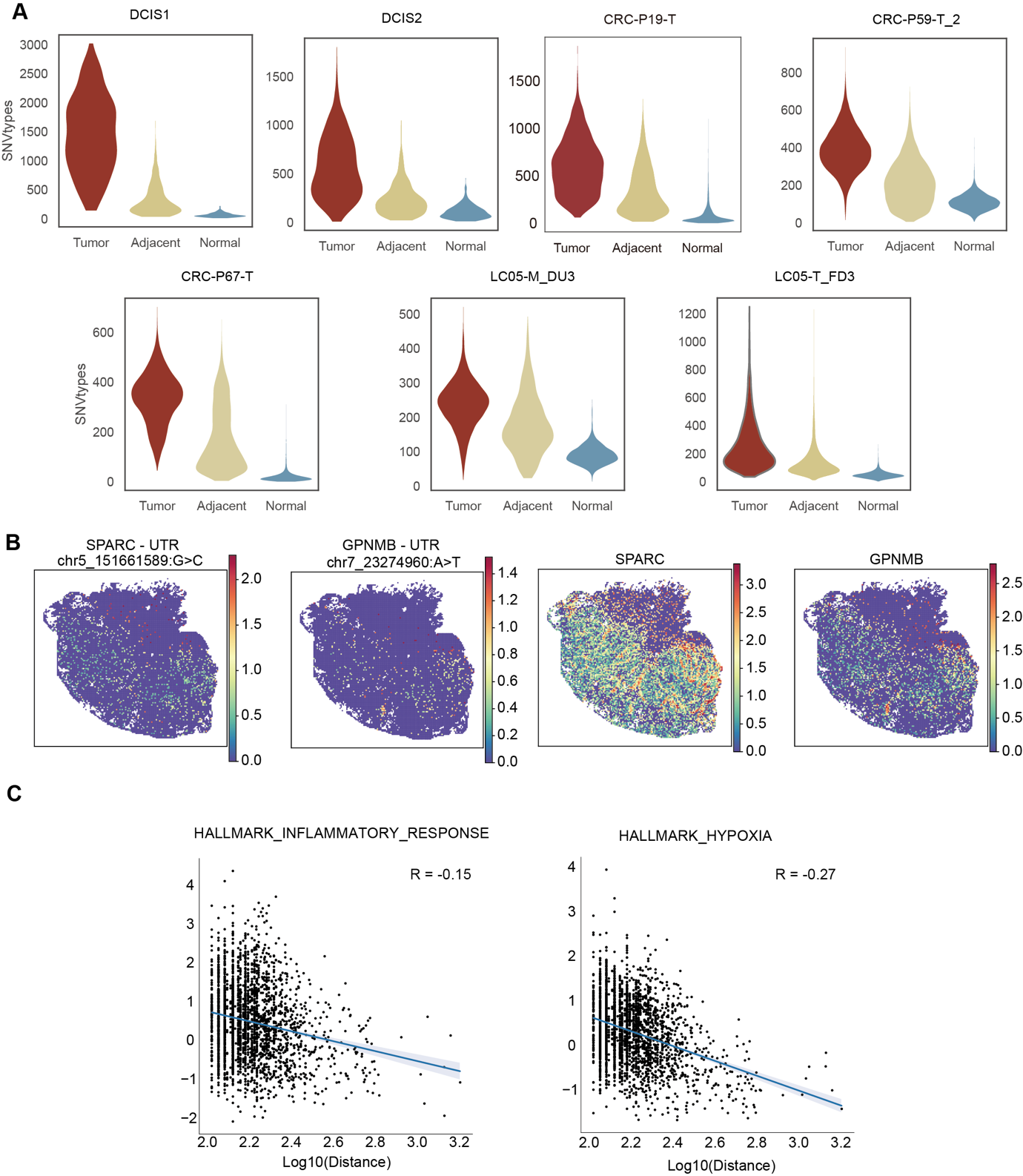


Supplementary Figure 6. Characteristics of SNVs at Tumor Margins

**(A)** Violin plot illustrating the distribution differences of SNV types across cluster regions in various sections. **(B)** Spatial visualization of *SPARC* and *GPNMB* gene expression and the distribution of corresponding representative SNVs. **(C)** Scatter plot showing the correlation between GSVA scores for inflammatory response and hypoxia with normalized SNV counts on spatial spots, including the Pearson correlation coefficient (R).


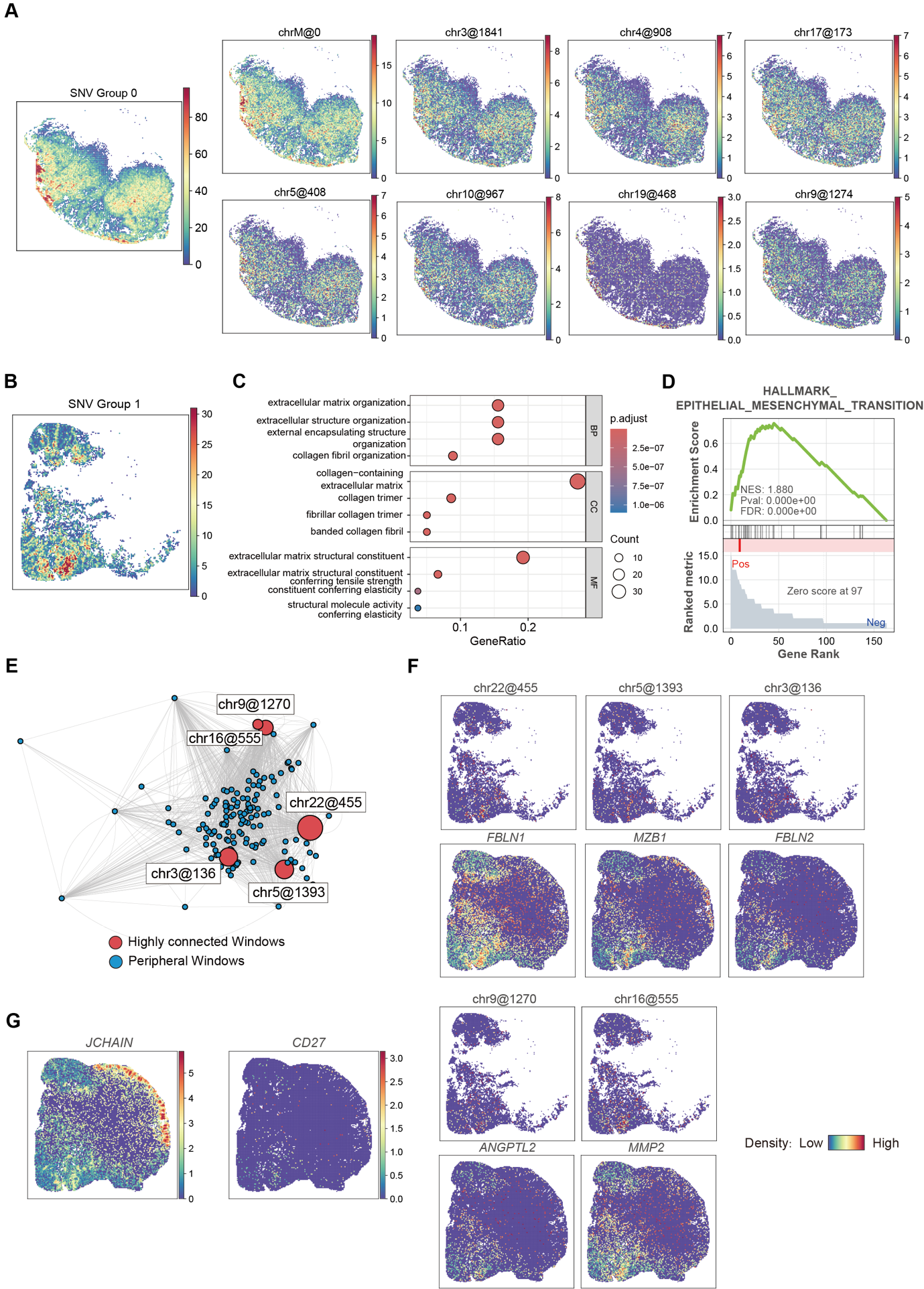


Supplementary Figure 7. SNV Group Reveals the Tumor Microenvironment

**(A)** Spatial visualization of SNV Group 0, including the top 8 SNVs ranked by Moran’s index. **(B)** Spatial visualization highlighting SNV Group associated with tumor-adjacent regions in the CRC-P19-T. **(C)** GO term analysis of genes within the SNV windows of SNV Group 1 in CRC-P19-T. **(D)** Gene Set Variation Analysis illustrating the enriched pathways for genes within all SNV windows of SNV Group 1 of CRC-P19-T. **(E)** Network graph showing the connectivity among SNV windows in SNV Group 1 of CRC-P19-T, with points representing SNV windows and line lengths indicating the degree of correlation. **(F)** Spatial visualization of the spatial distribution of highly connected SNV windows (top) and the representative genes contained within these windows (bottom). **(G)** Spatial visualization of markers associated with B cells in the CRC-P19-T section.


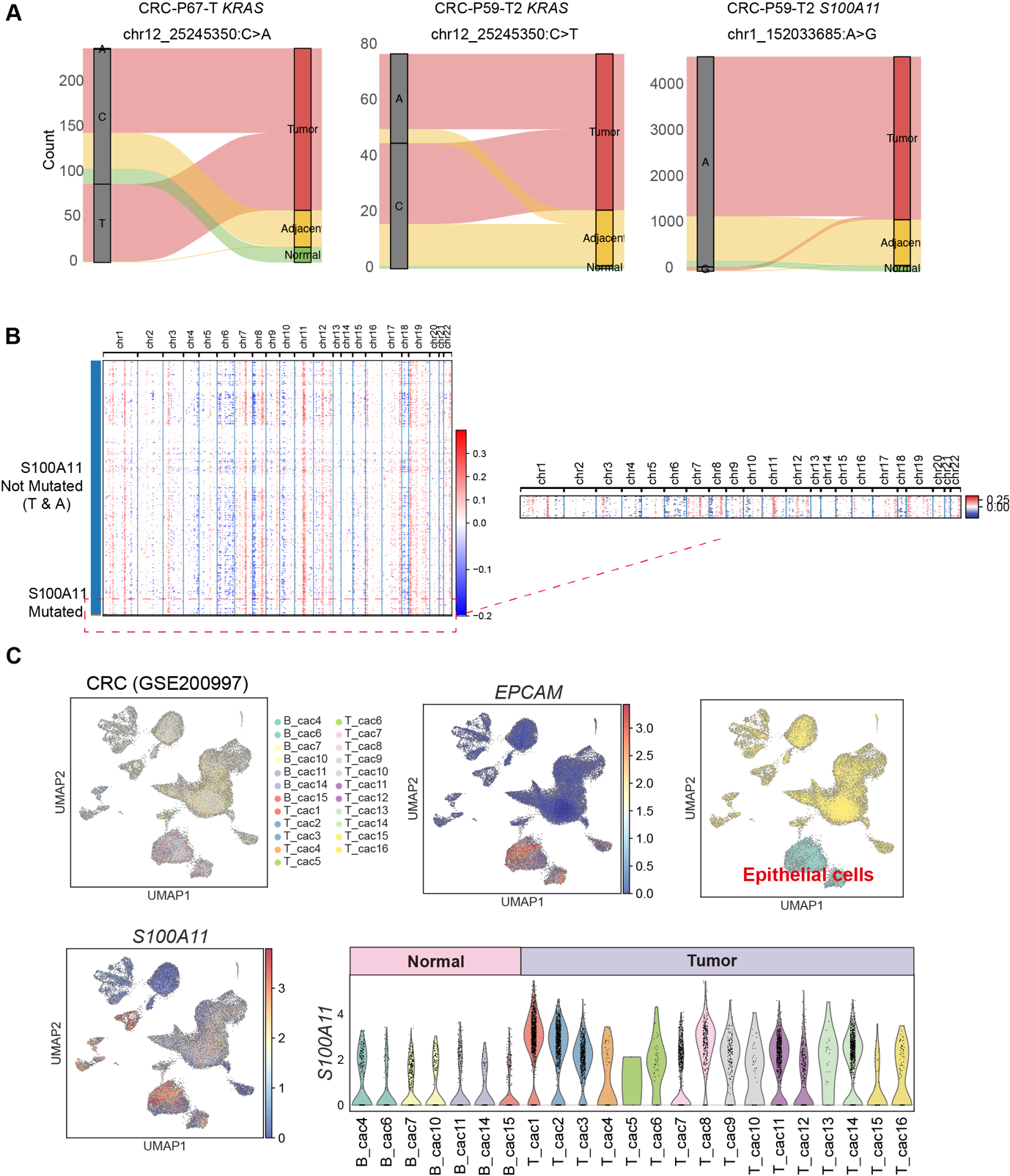


Supplementary Figure 8. Predicting Neoantigens from Spatial SNVs

**(A)** Sankey diagram illustrating the differences in mutated and reference base occurrences across various cluster regions. **(B)** Heatmap generated by inferCNV displaying inferred CNA profiles for mutated and non-mutated spots of S100A11 in the CRC-P59-T2 sample. **(C)** Single-cell RNA-seq analysis from GSE200997. Top: UMAP projection showing different clusters and epithelial cell distributions. Bottom: Both UMAP projection and violin plot highlighting the specific expression of S100A11 in tumor cells.

Supplementary Table 1. Potential neoantigens from the three CRC sample

| **Sample** | **Mutated peptide** | **WT peptaide** | **Gene** |
| --- | --- | --- | --- |
|  | DGVGKSAL | GGVGKSAL | *KRAS* |
|  | GADGVGKSA | GAGGVGKSA | *KRAS* |
|  | KLVVVGADGV | KLVVVGAGGV | *KRAS* |
| **CRC-P59-T1** | VVVGADGVGK | VVVGAGGVGK | *KRAS* |
|  | GADGVGKSAL | GAGGVGKSAL | *KRAS* |
|  | VGADGVGKSAL | VGAGGVGKSAL | *KRAS* |
|  | IMAHCILDL | IIAHCILDL | *SRP9* |
|  | LSKTEFPSF | LSKTEFLSF | *S100A11* |
|  | IMAHCILDL | IIAHCILDL | *SRP9* |
|  | AAADAAAAA | AAAAAAAAA | *TBL1XR1* |
|  | IDEQENWQEGKENI | IDEQENWHEGKENI | *LAP3* |
|  | DGVGKSAL | GGVGKSAL | *KRAS* |
| **CRC-P59-T2** | GADGVGKSA | GAGGVGKSA | *KRAS* |
|  | KLVVVGADGV | KLVVVGAGGV | *KRAS* |
|  | VVVGADGVGK | VVVGAGGVGK | *KRAS* |
|  | GADGVGKSAL | GAGGVGKSAL | *KRAS* |
|  | VGADGVGKSAL | VGAGGVGKSAL | *KRAS* |
|  | TLSSLTSSIL | TLSPLTSSIL | *MUC3A* |
|  | KKKSGLSVR | EKKSGLSVR | *WBP11* |
|  | IMAHCILDL | IIAHCILDL | *SRP9* |
|  | LSKTEFPSF | LSKTEFLSF | *S100A11* |
|  | VGVGKSAL | GGVGKSAL | *KRAS* |
|  | YKLVVVGAV | YKLVVVGAG | *KRAS* |
|  | VVGAVGVGK | VVGAGGVGK | *KRAS* |
| **CRC-P67-T** | KLVVVGAVGV | KLVVVGAGGV | *KRAS* |
|  | VVVGAVGVGK | VVVGAGGVGK | *KRAS* |
|  | TEYKLVVVGAV | TEYKLVVVGAG | *KRAS* |
|  | YKLVVVGAVGVGKSA | YKLVVVGAGGVGKSA | *KRAS* |
|  | KLVVVGAVGVGKSAL | KLVVVGAGGVGKSAL | *KRAS* |
|  | TLSSLTSSIL | TLSPLTSSIL | *MUC3A* |

Supplementary Table 2. HLA genotyping prediction

| **Sample** | **HLA-A** | **score** | **HLA-B** | **score** | **HLA-C** | **score** |
| --- | --- | --- | --- | --- | --- | --- |
| CRC-P59-T1 | HLA-A03:279N | 1 | HLA-B67:02 | 60 | HLA-C07:02 | 48 |
|  | HLA-A11:275 | 6 | HLA-B07:381 | 60 | HLA-C04:470 | 17 |
| CRC-P59-T2 | HLA-A11:01 | 60 | HLA-B67:02 | 60 | HLA-C07:722 | 4 |
| CRC-P67-T | HLA-A11:01 | 48 | HLA-B07:386N | 60 | HLA-C07:02 | 60 |
|  | - | - | HLA-B40:446 | 60 | - | - |
