# Supplementary material for: SpatialSNV: A novel method for identifying and analyzing spatially resolved SNVs in tumor microenvironments
Source: Gigascience. 2025 Jun 14;14:giaf065. doi: 10.1093/gigascience/giaf065 (PMC12166308; doi:10.1093/gigascience/giaf065)
Supplement: giaf065_GIGA-D-24-00553_Revision_2 [file giaf065_giga-d-24-00553_revision_2.pdf]

// GigaScience

SpatialSNV: A Novel Method for Identifying and Analyzing Spatially Resolved SNVs in Tumor Microenvironments

--Manuscript Draft--

Manuscript Number:	GIGA-D-24-00553R2
Full Title:	SpatialSNV: A Novel Method for Identifying and Analyzing Spatially Resolved SNVs in Tumor Microenvironments
Article Type:	Technical Note
Funding Information:	
Abstract:	Background<br>The dynamics of single nucleotide variants (SNVs) play a critical role in understanding tumor development, yet their influence on shaping tumor microenvironments remains largely unexplored. Spatial transcriptomics offers an opportunity to map SNVs within the tumor context, potentially uncovering new insights into tumor microenvironment dynamics.<br>Results<br>This study developed SpatialSNV for identifying effective SNVs across tumor sections using multiple spatial transcriptomics platforms. The analysis revealed that SNVs reflect regional tumor evolutionary traces and extend beyond RNA expression changes. The tumor margins exhibited a distinct mutational profile, with novel SNVs diminishing in a distance-dependent manner from the tumor boundary. These mutations were significantly linked to inflammatory and hypoxic microenvironments. Furthermore, spatially correlated SNV groups were identified, exhibiting distinct spatial patterns, and implicating specific roles in tumor-immune system crosstalk. Among these, critical SNVs such as S100A11L40P in colorectal cancer were identified as tumor region-specific mutations. This mutation, located within exonic nonsynonymous regions, may produce neoantigens presented by HLAs, marking it as a potential therapeutic target.<br>Conclusions<br>SpatialSNV represents a promising framework for unraveling the mechanisms underlying tumor-immune crosstalk within the tumor microenvironment by leveraging spatial transcriptomics and SNV-based tissue domain characterization. This approach is designed to be scalable, integrative, and adaptable, making it accessible to researchers aiming to explore tumor heterogeneity and identify therapeutic targets.
Corresponding Author:	Young Li, Ph.D<br>BGI Research, Hangzhou<br>Hangzhou, Zhejiang / 浙江 CHINA
Corresponding Author Secondary Information:	
Corresponding Author's Institution:	BGI Research, Hangzhou
Corresponding Author's Secondary Institution:	
First Author:	Yi Liu
First Author Secondary Information:	
Order of Authors:	Yi Liu
	Fan Zhu
	Xinxing Li
	Xiangyu Guan
	Yong Hou
	Yu Feng
	Xuan Dong

Powered by Editorial Manager® and ProduXion Manager® from Aries Systems Corporation

	YOUNG Li, Ph.D
**Order of Authors Secondary Information:**	
**Response to Reviewers:**	Dear Editor,<br>We sincerely appreciate the attention and valuable assistance provided regarding our manuscript. In response to the comments and requirements, the following revisions have been made:<br>1. The manuscript format has been thoroughly revised to align with the specified guidelines.<br>2. In the Methods section, URLs have been replaced with RRIDs to standardize the citation format, ensuring better compliance with journal standards.<br>3. Updated figures have been provided, incorporating all necessary modifications and improvements. For example, we have updated Figure 1 panels B, D, E, and F; Figure 4 panels I and J; and Figure 6 panels A-D.<br>4. Additionally, the GitHub repository in the RRID is https://github.com/ly4dsg/SpatialSNV. We will add a redirect link to https://github.com/YoungLi88/SpatialSNV/ in this GitHub repository. Meanwhile, we are applying via email to update the GitHub website in the RRID.<br>We believe these revisions have significantly enhanced the quality and clarity of the manuscript. We are grateful for the opportunity to improve our work and hope that the revised version meets the journal's standards for publication.<br><br>Thank you for your time and consideration. Sincerely yours,<br>Young Li on behalf of all authors<br>Young Li, Ph.D., BGI Research, Hangzhou 310030, China.<br>Email: liyang13@genomics.cn
**Additional Information:**	
**Question**	**Response**
Are you submitting this manuscript to a special series or article collection?	No
**Experimental design and statistics**<br><br>Full details of the experimental design and statistical methods used should be given in the Methods section, as detailed in our Minimum Standards Reporting Checklist. Information essential to interpreting the data presented should be made available in the figure legends.<br><br>Have you included all the information requested in your manuscript?	Yes
**Resources**<br><br>A description of all resources used, including antibodies, cell lines, animals and software tools, with enough information to allow them to be uniquely identified, should be included in the Methods section. Authors are strongly	No

encouraged to cite Research Resource Identifiers (RRIDs) for antibodies, model organisms and tools, where possible.<br><br>Have you included the information requested as detailed in our Minimum Standards Reporting Checklist?	
If not, please give reasons for any omissions below.<br><br>as follow-up to "**Resources**<br><br>A description of all resources used, including antibodies, cell lines, animals and software tools, with enough information to allow them to be uniquely identified, should be included in the Methods section. Authors are strongly encouraged to cite Research Resource Identifiers (RRIDs) for antibodies, model organisms and tools, where possible.<br><br>Have you included the information requested as detailed in our Minimum Standards Reporting Checklist?<br><br>"	Subsequent additions
**Availability of data and materials**<br><br>All datasets and code on which the conclusions of the paper rely must be either included in your submission or deposited in publicly available repositories (where available and ethically appropriate), referencing such data using a unique identifier in the references and in the "Availability of Data and Materials" section of your manuscript.<br><br>Have you have met the above requirement as detailed in our Minimum Standards Reporting Checklist?	Yes
GigaScience has policies and guidelines	Yes

in place for the use of generative AI-writing tools such as ChatGPT. If you have used such writing tools to assist with writing the manuscript this must be declared and cited in the text. Authors should not list AI-writing tools and other AI-assisted technologies as an author or co-author and should acknowledge that they are fully responsible for text generated or refined by AI-writing tools.<p><br><br>A summary of use (particularly in the introduction or among methods) needs to be included at the end of the paper, and the outputs should also be included as a supplementary file hosted in GigaDB or other open repositories. Please read our guidelines for more information. <p><br><br>By submitting to GigaScience, you are aware of the journal's AI-writing tools policy, and if you have declared use of such tools below, you have acknowledged this where appropriate in your manuscript and have made a summary of use and outputs available. <p><br><b>AI-assisted writing tools have been used in the preparation of this manuscript?	

Powered by Editorial Manager® and ProduXion Manager® from Aries Systems Corporation

Manuscript

Click here to access/download;Manuscript;Manuscript_gigascience_method

SpatialSNV: A Novel Method for Identifying and Analyzing Spatially Resolved SNVs in Tumor Microenvironments

Yi Liu[1, 2], Fan Zhu[1, 2], Xinxing Li[2], Xiangyu Guan[1, 2], Yong Hou[1, 3], Yu Feng[2], Xuan Dong[2], Young Li[2, 4]

1. College of Life Sciences, University of Chinese Academy of Sciences, Beijing 100049, China.

2. BGI Research, Hangzhou 310030, China

3. BGI Research, Shenzhen, 518083, China

4. Zhejiang Key Laboratory of Spatial Omics, Zhejiang, China.

Correspondence: liyang13@genomics.cn

Abstract

Background

The dynamics of single nucleotide variants (SNVs) play a critical role in understanding tumor development, yet their influence on shaping tumor microenvironments remains largely unexplored. Spatial transcriptomics offers an opportunity to map SNVs within the tumor context, potentially uncovering new insights into tumor microenvironment dynamics.

Results

This study developed SpatialSNV for identifying effective SNVs across tumor sections using multiple spatial transcriptomics platforms. The analysis revealed that SNVs reflect regional tumor evolutionary traces and extend beyond RNA expression changes. The tumor margins exhibited a distinct mutational profile, with novel SNVs diminishing in a distance-dependent manner from the tumor boundary. These mutations were significantly linked to inflammatory and hypoxic microenvironments. Furthermore, spatially correlated SNV groups were identified, exhibiting distinct spatial patterns, and implicating specific roles in tumor-immune system crosstalk. Among these, critical SNVs such as $S100A11^{L40P}$ in colorectal cancer were identified as tumor region-specific mutations. This mutation, located within exonic nonsynonymous regions, may produce neoantigens presented by HLAs, marking it as a potential therapeutic target.

Conclusions

SpatialSNV represents a promising framework for unraveling the mechanisms underlying tumor-immune crosstalk within the tumor microenvironment by leveraging spatial transcriptomics and SNV-based tissue domain characterization. This approach is designed to be scalable, integrative, and adaptable, making it accessible to researchers aiming to explore tumor heterogeneity and identify therapeutic targets.

Keywords

Spatial transcriptome, Tumor neoantigens, Single nucleotide variants

Background

Mutation is one of the hallmarks of tumorigenesis, playing a crucial role in programming tumor pathogenesis [1], which emerges mainly in three forms, including single-nucleotide variant (SNV), insertion

and deletion (Indel), and structural variation (SV). Indels and SVs, such as copy number variants (CNVs), involve long-range genomic alterations that are occasionally acquired during tumorigenesis and remain relatively stable during tumor expansion. SNVs are the most common type of genetic variant, occurring at various loci throughout the genome [2]. The majority of actionable causal mutations in diseases, including cancers and many other genetic disorders, are SNVs [3]. Consequently, sequencing targeted gene panels has dramatically transformed and facilitated disease diagnosis and the customization of therapeutic strategies over the past decade [4]. SNVs accumulate during tumorigenesis and exhibit intercellular heterogeneity[5]. Therefore, SNVs are theoretically more capable of tracing the dynamics of tumor development. However, creating a comprehensive map of SNVs with high resolution and precision remains challenging.

Single-cell sequencing allows us to investigate tumor heterogeneity at single-cell resolution and has greatly extended our knowledge about tumor evolution [6]. However, losing tissue context information makes revealing intra-tumor clonal ecosystems and progression difficult. It is essential to capture tumor mutations in situ to address these issues. Recently, spatially resolved sequencing technologies have become powerful tools for uncovering tissue slides' molecular and cellular landscapes, including clonal distribution, genetic variation, and tumor cell evolution [7, 8]. The spatial structure of intra-tumor CNVs can be inferred using spatial transcriptome and genome sequencing [7, 9]. Probe-based *in situ* sequencing has been employed to illustrate the clonal mutation heterogeneity of tumor tissues [10]. While some studies have restored SNVs to their spatial context [7, 11-13], these works mostly focused on the clonal evolution of tumors. There remains a lack of integration across multiple platforms, standardized data formats, and, more importantly, a comprehensive understanding of how spatial SNVs shape the tumor microenvironment in situ.

Here, we developed SpatialSNV for calling and analyzing effective SNVs on tumor tissues using spatial transcriptome data. We normalized the SNV count against the total unique molecular identifiers (UMI) counts per spatial spot to mitigate the impact of sequencing depth. By further exploring the spatially distinct SNVs, we demonstrated that SNV patterns are more suitable for tracing the spatial clonal evolution of tumors. To gain deeper insights into tumor microenvironments, we focused on the tumor margins, a region known for its high genetic and expressive heterogeneity [8, 14, 15]. We found that the mutational burden in this region declined in a distance-dependent manner, associated with the inflammatory and hypoxic conditions in the tumor margin microenvironment. Furthermore, we used SpatialSNV to identify spatially correlated

SNVs, termed SNV groups, and found that SNVs within the same groups exhibited similar spatial distributions. SNV groups may reflect the states of tumor cells and surrounding cells in the microenvironment at the tumor margin. We further observed that critical tumor SNVs, such as tumor driver mutations, exhibit specific spatial patterns within the tissue context. Examination of exonic nonsynonymous SNVs also identified $KRAS^{G12D}$, $KRAS^{G12V}$, and $S100A11^{L40P}$ as tumor region-specific mutations in CRC samples, which may generate neoantigens presented by the human leukocyte antigens (HLAs), representing the potential therapeutic target. Notably, single-cell RNA-seq analysis showed that *S100A11* was highly expressed in CRC tumor cells, indicating a potential therapeutic target of $S100A11^{L40P}$. SpatialSNV integrates spatial SNV and transcriptomic data to unveil a more comprehensive genetic map of tumor tissue, enhancing our understanding of how SNVs shape the tumor microenvironment. Moreover, effective SNVs can be used to pinpoint tumor-specific exonic nonsynonymous mutations, providing potential therapeutic targets for developing precision medicine strategies.

Analyses

SpatialSNV enables effective SNV calling on spatial transcriptomics data

To investigate SNVs within tumors *in situ*, SpatialSNV utilized Mutect2 [16] to call somatic mutations, using PON (panel of normal) germline resource from the GATK resource bundle to filter out potential germline mutations (Methods, Fig. 1A). At the same time, we tested the performance of spatialSNV on multiple spatial transcriptomics platforms, including Stereo-seq [15, 17, 18], Visium [19], Slide-seq [20] and Slide-DNA-seq [9] (Supplementary Fig. 1A). To eliminate false positive callings caused by low sequencing depth, we examined the number of unique reads supporting each SNV (Supplementary Fig. 1B) and only retained those SNVs supported by more than 20 unique reads. Furthermore, we examined the spatial distribution of SNVs in representative samples including “DCIS1”, “CRC-P19-T”, “Slide-RNA-CRC”, “Slide-DNA-CRC”(Fig. 1B). Corresponding spatial transcriptomic profiles were also prepared to assist in the analysis of spatial SNVs (Supplementary Fig. 1C). Most SNVs appeared in only a small number of spots, likely due to technical noise. SpatialSNV regarded SNVs detected in multiple spots as effective SNVs, which might indicate similar evolutionary progress in tissue. Following this quality control process, we found that Stereo-seq had a relatively high number of effective SNVs, likely due to its higher sequencing depth. We then assessed the correlation between the number of SNV types and the number of unique reads per sample.

As anticipated, datasets with higher sequencing depths generally resulted in more effective SNV callings (Fig. 1C, Supplementary Fig. 1D). Notably, due to the extremely low sequencing depth, SpatialSNV could only recover 23 effective SNVs in Slide-DNA-seq data (Supplementary Fig. 1D-E). Therefore, Slide-DNA-seq data were not included in further analysis. Subsequently, we annotated the genomic locations of these effective SNVs and found that SNVs identified through spatial transcriptomics predominantly originate from gene body regions, with a considerable proportion in untranslated region (UTR) regions (Fig. 1D-E). Then we compared the spatial distribution of effective SNVs derived from public data on the Stereo-seq platform (Fig. 1F). The high consistency of SNVs among samples from the same patient demonstrates the robust SNVs calling pipeline of SpatialSNV. Moreover, the high correlation of SNVs within the same tumor and across different tumors suggests associations with driver mutations during tumorigenesis. Consequently, we conducted a Gene Ontology (GO) term enrichment analysis on genes affected by common SNVs across all samples (Fig. 1G). These SNVs impact molecular functions such as cadherin binding and transcription coregulator activity, which are associated with aberrant transcriptional activity[21]. Then, we observed a strong linear association between SNV raw count and gene expression (Supplementary Fig. 2B). To reduce the influence of gene expression and sequencing depth on SNV quantity, SpatialSNV converted the SNV count matrix to a binary matrix and normalized the SNV counts against the total mRNA UMI captured. With this correction, we obtained distinct spatial profiles between transcriptomics and SNVs (Supplementary Fig. 2A) and a significant correlation decrease between RNA expression and SNV counts (Supplementary Fig. 2B). We then observed significant enrichment of SNV mutations on the tumor regions on tumor sections across different spatial transcriptomics platforms (Fig. 1H).

SpatialSNV identifies Tumorigenesis-associated SNVs

Gene mutations play a crucial role in the onset and progression of tumors. To explore whether SpatialSNV is able to identify essential SNVs in tumorigenesis, we utilized transcriptomics data to divide the tumor section into the tumor (marked by *EPCAM* and other tumor-associated markers), tumor-adjacent margins (marked by *VIM*, *COL1A2*, *PTPRC*), and normal tissues (Fig. 2A-B, Supplementary Fig. 3). Subsequently, we validated the reliability of RNA region clustering using the inferCNV(method) (Supplementary Fig. 4). Using the ductal carcinoma in situ (DCIS) section from the Visium platform and colorectal cancer (CRC) section from the Stereo-seq platform as examples, we calculated the SNV mutation

frequency and occurrence on each gene (Fig. 2C). We found the B-cell receptor related genes to be highly mutated with low occurrence, due to the somatic hypermutation progress during the development and maturation of B cells. Mitochondrial genes also exhibited high mutation frequency and relatively high occurrence, consistent with the accumulation of mitochondrial mutations in tumor cells [22]. We further compared the mutated genes with those detected in the BRCA and COAD cohorts from the TCGA database and found that most mutated genes were shared in the TCGA database (Fig. 2D). However, we observed that tumor driver genes such as *GATA3* and *ESR* in DCIS [23, 24] were almost uniformly distributed spatially, but their corresponding SNVs were only present in the tumor. The SNVs and gene expression exhibited different patterns. (Fig. 2E). Chi-square tests of SNVs and RNA with tumor incidence indicated that the occurrence of SNVs is more closely associated with tumor regions (Fig. 2F). Given the close association of SNVs and genes with tumorigenesis, we further examined the distribution of other driver genes and SNVs to provide additional support for this perspective. Despite these driver genes being widely expressed across different spatial regions, the SNVs associated with these driver genes were confined solely to the tumor region (Fig. 2G). Moreover, we performed Pearson correlation analysis between normalized SNV counts and hallmark gene sets from MSigDB[25] on tumor samples. We found that the mutation burden in both DCIS and CRC samples was highly correlated to pathways such as MYC regulation, oxidative phosphorylation, and *KRAS* signaling, which are known to play critical roles in tumorigenesis [26-28](Fig. 2H). These findings demonstrate that spatially resolved SNVs identified by SpatialSNV can offer genetic information from an additional dimension, extending beyond the insights provided by spatial transcriptomics data.

SpatialSNV helps to trace tumor development in *situ*

SNVs can serve as imprints to trace tumor development. We then explored a DCIS sample previously shown to have three subclones [19]. Consistent with the original study, we observed a pronounced clonal distribution from the transcriptome data (Fig. 3A, Supplementary Fig. 5A-B). We also analyzed the CNVs using the InferCNV algorithm, which confirmed the clonal diversity of the three tumor subclones (Fig. 3B). Additionally, to determine whether SNVs could reproduce the clustering patterns, we performed dimensionality reduction and clustering on the spots using SNV windows as features. This analysis revealed clustering results similar to those observed with CNVs (Supplementary Fig. 5C). We used SpatialSNV to

perform differential analysis on SNVs and observed different SNV profiles of three tumor subclones (Fig. 3C). For example, *THOP1* was evenly expressed in three subclones, but the associated SNV chr19_2813592:G>A occurred more frequently in subclone 1. Similarly, *PRSS23* expressed high in subclones 0 and 1, while the corresponding SNV chr11_86808877:T>C occurred predominantly in subclone 1 (Fig. 3D-E). The inconsistency of the spatial patterns between SNVs and gene expression indicated a dynamic clonal development progress within the tumor tissue.

To trace the clonal development *in situ* of tumor cells, we employed the Mononcle2 algorithm [29] to construct the developmental trajectory using the differentially distributed SNVs. We found that subclones 1 and 2 might have differentiated from subclone 0 (Fig. 3F), which is consistent with the CNV profiles of the subclones. Subclone 2 appeared to have CNV gains on chr3, and CNV loss on chr6 and chr11, and subclone 1 suffered CNV gain on chr5 (Fig. 3B). Therefore, subclone 1 and 2 would accumulate more mutations during clonal differentiation theoretically. As anticipated, we identified more SNVs in subclone 1 and 2 (Fig. 3G-H). We also observed that the differential SNVs such as chr7_98385752:A>G and chr19_2813592:G>A were elevated along clonal differentiation trajectories (Fig. 3I), indicating their contributions to the tumor clonal development. Moreover, we mapped the predicted pseudotime to the tumor section and found that tumor subclone 0 gradually differentiated into subclones 1 and 2 in the co-occurring tumor regions (Fig. 3J). Additionally, in subclone 2-specific tumor regions, spots on the tumor margins appeared at a later developmental stage (Fig. 3J), suggesting that subclone 2 was still undergoing rapid evolution. SpatialSNV facilitates the analysis of regional tumor evolution traces imprinted on spatially resolved SNVs.

SpatialSNV reveals mutational dynamics at the tumor margins

As tumors invade, the tumor margins often exhibit a chronic inflammatory microenvironment [30], which may trigger more mutation events. Consistently, we investigated the genetic landscape of the tumor tissues and observed that tumor margins gained significantly more SNVs than distant normal regions (Fig. 4A-B, Supplementary Fig. 6A). Moreover, we found that the tumor margins had unique mutational profiles (Fig. 4C-D). For example, SNVs chr20_4024544:C>T and chr20_4024581:C>T occurred more frequently in the tumor margins of the CRC-P59-T1 sample and exhibited a clear spatial preference for the tumor margins (Fig. 4D-E). When surveying their genomic loci, we found significantly more sequencing reads

covering this region in the tumor margins than in tumor and normal tissues, implying that these SNVs may alter gene expression (Fig. 4F). For instance, The mutation chr5_151661589:G>C is located in the UTR region of *SPARC*, which is highly expressed in metastatic tumors[31], potentially indicating the invasiveness of the tumor margin. Similarly, the UTR mutation chr7_23274960:A>T in *GPNMB* may also influence *GPNMB* expression, thereby promoting tumor metastasis[32] (Supplementary Fig. 6B). We further investigated the genes associated with SNVs predominantly present in tumor margins and found them to be highly related to the extracellular matrix and cell adhesion processes (Fig. 4G), suggesting that the tumor cell in the adjacent margins may help reshape the microenvironment, potentially contributing to tumor immune escape [33]. To verify this, we examined the inflammatory response and hypoxia processes, which are reported to be essential in tumor immune escape [34, 35]. We found that the expression of genes related to both pathways declined along with the normalized SNV count in the tumor margins as the distance increased from the tumor boundary (Fig. 4H, Supplementary Fig. 6C). This suggests that tumor expansion may lead to the emergence of new tumor cells at the margin, which could subsequently give rise to novel SNVs.

The relationship between the characteristics of the tumor margin microenvironment and tumor progression remains largely unknown. To address this, we plotted the normalized SNV counts against the distance to the tumor boundaries. We found that SNV accumulation was negatively correlated with the distance from the tumor boundary (Fig. 4I), a phenomenon similar to the higher somatic copy number alteration (SCNA) burden observed in the tumor center[36]. Furthermore, we observed varying rates of decline in the mutational dynamics at the tumor margins across tumor samples and found that the rate of decline in mutational dynamics is highly correlated with the immune activity of the tumor section (Fig. 4J). Tumors with higher immune activity exhibited a slower decline in mutational dynamics, suggesting that the tumor margin may harbor more neoantigens, leading to the accumulation of immune cells.

Spatially correlated SNVs contribute to reshaping tumor microenvironments synergistically

Subsequently, we aimed to identify specific SNVs responsible for reshaping the microenvironment of the tumor margins. To reduce the sparsity of the spatial SNV matrices, SpatialSNV partitioned the genome into 100,000 bp windows and aggregated SNV events in each window for each spatial spot. We hypothesized that SNVs occurring in close spatial proximity are genetically associated. Therefore, we calculated the spatial

correlations between SNV windows and constructed a connectivity graph of the SNV windows for SNV cluster detection (Fig. 5A). Using the Leiden algorithm, we partitioned SNVs into different groups, termed SNV groups. SNV groups exhibited more significant spatial distribution patterns than the individual SNVs they contained (Fig. 5B, Supplementary Fig. 7A). The spatial patterns of SNV groups differed from each other (Fig. 5C), indicative of different biological processes. Consequently, we examined the corresponding genes of each SNV group and found them to be involved in distinct GO terms. For example, SNV Group 1 was primarily associated with chemokine activity, while SNV Group 2 mainly affected components related to the extracellular matrix (Fig. 5D), consistent with the specific spatial distribution of SNV Group 2 at the tumor margins. Considering that the extracellular matrix is highly related to epithelial-mesenchymal transition (EMT) [37], we speculated that SNVs within SNV Group 2 may contribute to the tumor EMT process. We then performed GSVA [38] analysis and confirmed that genes associated with SNVs within SNV group 2 were highly enriched in the EMT pathway (Fig. 5E). Similar SNV groups were also observed across tumor samples. For instance, SNV Group 1 of the CRC-P19-T sample was distributed at the tumor margins and was also highly enriched in the EMT pathway (Supplementary Fig. 7B-D). These data suggested that genes involved in similar biological processes may mutate synergistically.

To reveal the potential connections between SNV groups, we analyzed the levels of calculated connectivity between SNVs (Fig. 5F). We found that some SNV windows had higher connectivity than others. In SNV Group 2, for example，chr22@328, chr7@232, and chr19@449 were associated with TIMP3, GPNMB, and APOE, respectively, which are marker genes of tumor-associated macrophages (TAMs) (Fig. 5G). This suggests that SNV Group 2 may reflect the presence of TAMs in the tumor microenvironment and the genomic alterations driven by their strong transcriptional activity. Therefore, we examined the TAM signature and observed that the TAM signal was highly co-localized with SNV group 2 (Fig. 5H). Moreover, we found that the pleiotrophin (PTN) signaling pathway was also highly expressed around these regions, suggesting that SNV group 2 may be responsible for the TAM microenvironment at the tumor margins. In the CRC-P19-T section, SNV Group 1 contained highly connected SNV windows such as chr22@455, chr3@136, chr16@555 (Supplementary Fig. 7E), and associated with tumor metastasis-related genes of *FBLN1, FBLN2,* and *MMP2* [39-41], respectively. Another highly connected SNV window, chr5@1393, is associated with *MZB1* (Supplementary Fig. 7F), an endoplasmic reticulum stress-related gene in B-cells [42]. We also observed B cell and plasma cell marker genes such as *JCHAIN* and *CD27* in SNV

Group 1 enriched region (Supplementary Fig. 7G), suggesting high transcriptional activity of B cells and plasma cells. These findings showed that SpatialSNV is able to identify SNV groups comprised of SNVs with similar spatial patterns and biological functions, which contribute to the tumor microenvironment synergistically.

SpatialSNV identifies tumor region-specific exonic nonsynonymous SNVs as potential neoantigens

Tumorgenesis-assocaited SNVs may serve as therapeutic targets. Specifically, those exonic nonsynonymous SNVs may result in neoantigens and subsequently activate the adaptive immune system. To identify potential neoantigens from exonic nonsynonymous SNVs *in situ*, SpatialSNV used sliding window translation of exonic mutations to predict potential neoantigen peptides and filtered out normally expressed peptides by searching public databases and protein from the human coding DNA sequence (CDS) (Fig. 6A). In CRC samples (CRC-P59-T1, CRC-P59-T1, CRC-P67-T), we found thousands of mutations occurring in gene exon regions, most of which were nonsynonymous SNVs (Fig. 6B). We observed that these nonsynonymous SNVs from different samples of the same patient overlapped significantly, while those from other patients also shared a substantial proportion of nonsynonymous SNVs (Fig. 6C), indicating potential therapeutic neoantigens for CRC.

To identify potential therapeutic neoantigens from the nonsynonymous SNVs, we used the HLA Ligand Atlas database [43] and protein CDS to exclude epitopes presented in healthy individuals. Subsequently, we searched the IEDB[44], MHC Motif Atlas[45], and dbPepNeo [46] to find known peptides presented by HLAs (Fig. 6D). We identified 64 potential neoantigens from the three CRC samples (Fig. 6D, Supplementary Table 1). We then examined the corresponding genes of these neoantigens and found that mutated peptides from *KRAS*, *SRP9*, *MUC3A*, and *S100A11* appeared in multiple samples (Fig. 6E). *KRAS* mutations appeared in approximately 40% of CRC patients, predominantly $KRAS^{G12D}$ and $KRAS^{G12V}$ mutations [47]. T cells targeting $KRAS^{G12D}$ and $KRAS^{G12V}$ have been proven to control tumors efficiently [48, 49]. *MUC3A* can promote the progression of colorectal cancer through the PI3K/Akt/mTOR pathway [50] and has been predicted to be a potential CAR-T therapy target [51]. We traced the spatial distribution of these nonsynonymous mutations and observed that although *KRAS* and *S100A11* were expressed in various regions, the mutated gene form was mainly restricted in the tumor regions (Fig. 6F, Supplementary Fig. 8A), suggesting potential neoantigens.

Mutated peptides should be presented by the HLA molecules to serve as neoantigens. We determined the HLA types of the patients from the spatial transcriptomics data using T1K [52] and found both patients to be HLA-A*11:01 and HLA-C*07:02 allele types (Supplementary Table 2). We then performed HLA affinity prediction of the mutated peptides using TransPHLA [53]. We found that mutated $KRAS^{G12D}$ and $KRAS^{G12V}$ peptides were predicted to have strong affinity with HLA-A*11:01, with a $KRAS^{G12V}$ peptide also predicted to be presented by HLA-C*07:02 (Fig. 6G). Considering that HLA-A*11:01 is one of the most abundant HLA alleles, especially in East Asia [54], these mutated KRAS peptides can potentially be good TCR-T therapy targets. Besides mutated KRAS peptides, we also found a peptide "LSKTEFPSF" derived from $S100A11^{L40P}$ to have a strong affinity with HLA-C*07:02 (Fig. 6G). To verify whether *S100A11* is expressed in CRC tumor cells, we explored the CNV profile of the spots with $S100A11^{L40P}$ mutation and found them to have significant genome alterations (Fig. 6H, Supplementary Fig. 8B). Moreover, we examined the *S100A11* expression in published CRC single-cell sequencing data [55] and confirmed that *S100A11* is highly expressed in CRC tumor cells (Fig. 6I-K, Supplementary Fig. 8C). These data suggested that $S100A11^{L40P}$ can serve as a potential therapeutic target for precision medicine design.

Discussion

In this study, we developed SpatialSNV for calling and analyzing effective SNVs from spatial transcriptomics data. Incorporating spatial mutation information provides a more comprehensive tissue context of tumors. We observed significant platform bias when calling SNVs from spatial transcriptomics data generated by different techniques, likely attributed to the varying RNA capture efficiency and sequencing depth among the platforms. Notably, Stereo-seq data tend to have higher total UMI counts, enhancing effective SNV calling [56]. Although SpatialSNV was developed on spatial transcriptomics data, calling SNVs from transcriptomics data still has limitations: a relatively higher false positive rate [6, 57] and high correlation with gene expression, compared to DNA-seq data. We selected SNVs supported by multiple unique reads and observed across several spots to reduce the false positive error rate. Furthermore, we normalized the SNV counts against the sequencing depth (total UMI count) at each spot, which has been proved to reduce the influence of gene expression on SNV quantities efficiently. Thus, our study provides a relatively robust method to recover SNV information from spatial transcriptomics data. Moreover, SpatialSNV can also be modified for spatial DNA sequencing data such as Slide-DNA-seq. Due to the poor

sequencing depth, we could not recover enough SNVs for subsequent analysis, demonstrating that calling SNVs from spatial DNA-seq data remains a big challenge. More precise spatial SNV analyzing will need a further developed spatial DNA-seq technology with higher data quality and deeper sequencing depth. It is also important to note that platforms such as Visium, Stereo-seq, and Slide-seq are poly(T) capturing-based sequencing methods, which may introduce a strong bias toward SNVs near the 3' end. Recent spatial techniques, such as Patho-DBiT [58], could provide a relatively even gene body coverage. This experimental advancement may help to improve the efficiency in capturing variations across the gene body.

Many cancer cells at solid tumor margins exhibit reversible invasiveness, coordinated by a developmental regulatory program known as EMT [59]. Specific conditions, such as hypoxia and factors such as cytokines secreted by stromal cells, can induce EMT, thereby promoting invasiveness [27]. EMT regions are associated with tumor progression and often coexist with immune responses. A high degree of immune infiltration is closely linked to EMT, suggesting that EMT progress closely interacts with the immune system. Our data showed that the mutational dynamics of the tumor margins are involved in hypoxic and immune regulatory processes, which are essential in shaping the tumor microenvironment and promoting tumor expansion [60]. We recognized that mutations at the tumor margins may originate from newly proliferating tumor cells, which may influence the tumor microenvironment in the peripheral regions. However, it is important to note that somatic mutation frequencies are also high in normal non-blood tissues[61]. Therefore, when examining the mutational landscape of the tumor microenvironment, the extent of the influence of tumor cells on this landscape remains unclear. Although the tumor center in ccRCC has been characterized by proliferation, necrosis, and hypoxia[36], the tumor margin exhibits significant collagen deposition, leading to increased ECM density and stiffness, which further reduces oxygen supply and creates a hypoxic environment[62]. By integrating SNVs with the spatial context, we identified spatially correlated SNVs, termed SNV groups. We found that SNVs within the same group tend to be involved in similar biological processes. Moreover, we noticed that specific SNV groups are associated for the EMT process of the tumor margin regions, which are conserved across different tumor samples, indicating that these SNV groups may be critical factors in regulating the tumor margin environment. We observed these intriguing phenomena in the collected samples, but the potential role of SNV groups in EMT requires more data to support, as does the role of SNVs at the tumor margin. Moreover, the biological significance of SNV Groups may require further investigation. We hypothesize that SNVs appearing in other cells may indicate

high transcriptional activity in these cells within the tumor microenvironment, leading to certain genetic mutations. Since SNVs are essential sources of tumor-specific neoantigens, these SNVs can potentially be used as therapeutic biomarkers targeting tumor margin regions.

Although SNVs are the most prevalent type of mutation at the genomic level in tumor cells [63], CNVs involving tumor driver genes are generally regarded as the characteristics of many cancers[64, 65]. Analyzing SNVs and CNVs at the single-cell level allows for the reconstruction of tumor evolutionary pathways [66, 67], aiding in identifying tumor driver genes. Notably, tumor-specific neoantigens, resulting from mutations in tumor cells, present promising targets for cancer immunotherapy[63]. Although neoantigens can arise from structural variations such as CNVs [68], the majority of tumor neoantigens are derived from SNVs [69, 70]. In our study, we used SpatialSNV to identify tumor region-specific SNVs in CRC samples. By focusing on the nonsynonymous SNVs in exonic regions of protein-coding genes, we found potential neoantigens of the tumors and predicted their HLA affinities. With this approach, we identified $KRAS^{G12D}$, $KRAS^{G12V}$, and $S100A11^{L40P}$ mutations as potential therapeutic targets. $KRAS^{G12D}$ and $KRAS^{G12V}$ are common mutations used as targets for universal therapy strategies, while $S100A11^{L40P}$ is more likely an individually expressed mutation, which can be targeted in personalized therapies. We provided a neoantigen prediction guidance in this study, but not performed experimental validations. However, the *KRAS* associated neoantigens, which have been widely documented in the literature to be genuinely present in colorectal cancer, could demonstrate the reliability of our methodology to some extent. Therefore, spatially resolved SNVs are useful in finding therapeutic targets for precision medicine design.

Methods

Spatial transcriptomics data processing

For Visium (RRID:SCR_023571) spatial transcriptomics, we employed Space Ranger version 1.3.1 (RRID:SCR_025848) to generate binary alignment map (BAM) files, utilizing the hg38 reference genome (GRCh38-2020-A provided by 10x Genomics). For the analysis of Stereo-seq data, we utilized the Stereo-seq Analysis Workflow[71] (SAW V4.0, RRID:SCR_025001), with default parameters to align sequences against the hg38 reference genome, using the GRCh38.p12 GTF file for gene annotations. Slide-DNA-seq was performed in accordance with the established protocols . For Slide-RNA-seq, we implemented the Drop-

seq tools integrated within the Slide-seq Tools suite, aligning to the hg38 reference genome, consistent with the reference used for Visium analyses.

Analysis and visualization of spatial transcriptomics data were conducted using Scanpy[72] (RRID:SCR_018139). For the Stereo-seq platform, gene counts were aggregated within 100x100 spot blocks to construct the bin100 gene count matrix. Spots containing fewer than 100-200 genes and genes present in fewer than 10 spots were excluded to ensure data quality. Additional spot filtering was applied based on metrics such as n_genes_by_counts and pct_counts_mt, which were tailored to the specific characteristics of each sample. Counts were normalized using the normalize_total function in Scanpy, followed by dimensionality reduction through principal component analysis (PCA). The top 15 principal components were selected to construct neighbor graphs with a parameter setting of 15 neighbors. Clustering of spatial spots was performed using the Leiden algorithm to identify potential clusters. Spatial domains within the tissue, such as tumor region, adjacent margins, and normal region, were preliminarily categorized using marker genes, including *MKI67*, *TP53*, *EPCAM*, *VIM*, and *PTPRC*.

Inferred copy number variation (inferCNV) analysis

To validate the effectiveness of tumor regions identified from spatial transcriptomics, we used inferCNV to verify copy number variations in different areas further to determine the accuracy of the region segmentation. The gene matrix was used as input for inferCNV (inferCNV of the Trinity CTAT Project, RRID:SCR_025804). Simultaneously, chromosomal positions for all genes are annotated by searching the GTF file, which also serves as input for inferCNV. The cnv.tl.infercnv function was set with parameters: lfc_clip = 3, window_size = 250, and exclude_chromosomes=('chrX','chrY'). The normal region was selected as a reference for inferCNV analysis.

Expression track visualization

The BigWig files are created from BAMtype files using the program bamCoverage from deepTools(RRID:SCR_016366)[73] with parameters "--binSize 1", "--normalizeUsing RPKM", " --exactScaling", "--minMappingQuality 10". All BigWig data were stored in CyVerse (RRID:SCR_014531) [74] for uploading to UCSC genome Browser (RRID:SCR_005780)[75] for visualization.

Somatic mutation calling with mutect2

Aligned SAM files were converted to BAM files and sorted by coordinate using Samtools (RRID:SCR_002105)[76](version 1.11). To remove duplicated reads on spatial transcriptomics data, we

utilized a custom Python script to combine UMI and barcode tags. The MarkDuplicate function of Picard (RRID:SCR_006525) was applied to remove duplicated reads from each BAM file.

GATK's Mutect2[16] (RRID:SCR_026692, version 4.2.6.1) was used to call mutations according to their somatic pipeline . We first used addOrReplaceReadGroups to add group information to the BAM file, which facilitates the recognition of tumor files by Mutect2. Following GATK's recommendations for calling mutations on transcriptomes, we proceeded with splitNCigarReads and ApplyBQSR to process the BAM files, where ApplyBQSR's –known-sites parameter used dbsnp_151.hg38.vcf.gz from the GATK resource bundle. Mutect2's Tumor-only mode was employed for mutation calling in spatial transcriptomics with default parameters, where the germline resource and Panel of Normals were af-only-gnomad.hg38.vcf.gz and 1000g_pon.hg38.vcf.gz respectively, from the GATK resource bundle. Although Mutect2 is not the optimal choice for calling mutations from DNA, we utilized Mutect2 without the splitNCigarReads option for SNV calling in slide-DNA-seq.

Following SNV calling, we employed FilterMutectCalls to filter the SNVs, excluding those annotated with tags such as weak_evidence, germline, strand_bias, slippage, contamination, and panel_of_normals in the VCF file. We then traced the reads aligned to each SNV locus, extracting the coordinates of reads that contained the alternative base. These coordinates were used to construct a binary matrix, indicating the occurrence of specific SNV events at each spot. Specifically, on the Stereo-seq platform, SNV counts were aggregated within blocks of 100x100 spots to form the bin100 SNV count matrix. For an SNV to be considered effective, it was required to be supported by at least 20 unique reads and appear in at least five spots. Effective SNVs were annotated using ANNOVAR (RRID:SCR_012821)[77].

Gene mutation frequency

We quantified the number of effective SNV sites per gene to assess gene mutation frequency and normalized these figures by gene length. A higher gene mutation frequency suggests an increased mutational pressure on that gene.

Normalization of SNV counts

For the calculation of the Normalized SNV cont, in order to eliminate the impact of sequencing depth on SNV counts, we used the UMI counts of each spot to normalize the expression of SNVs.

$$Norm\ SNV_i = \log\left(\frac{s_i}{\sum_{j=1}^{n} u_j} + 1\right)$$

where i is the ith SNV, s_i represents the SNV count number, u_j represents the UMI count number, and n is the total number of genes.

Identification of tumor subclones and pseudotime analysis

The expression matrix from spots designated as tumor regions was extracted. This matrix was then normalized and subjected to dimensionality reduction clustering via Scanpy, consistent with the methods described in the "Spatial Transcriptomics Data Processing".

Next, normalized SNV count matrices were extracted from spots designated as tumor regions. Analytical objects were created using Monocle2's GaussianFF model (RRID:SCR_016339)[29]. SNVs for inferring spot pseudotime were identified by Scanpy as differential SNVs (log-fold change ⩾ 1, p-values < 0.5). Pseudotime ordering of different spots was determined using reduceDimension with norm_method=none and orderCells function. Visualization of SNVs across evolutionary branches was facilitated using Python.

Correlation between normalized SNV counts and distance

The KDTree function in the Python package scipy was used for Nearest Neighbor Search (NNS). The average distance and feature expression of the 5 (k = 5) nearest tumor region spots around each adjacent margins spot are calculated. We employed the scipy.stats.linregress function to fit a linear model between the logarithm of normalized SNV count and the logarithm of average distances from the tumor for each spot. The correlation between these was quantified using the coefficient of determination(R^2), derived from the squared Pearson correlation coefficient provided by the regression.

Enrichment analysis

For Gene Ontology (GO) analysis and visualization, we utilized the R package clusterProfiler (RRID:SCR_016884)[78]. GO enrichment analysis was conducted using the enrichGO function, targeting specific gene sets with the following key parameters: pAdjustMethod was set to 'BH' for multiple testing correction, and qvalueCutoff was established at 0.01 to filter significant terms.

The Gene Matrix Transposed File Format (GMT) file, h.all.v2023.1.Hs.symbols.gmt, was sourced from the MSigDB[79] database to facilitate these analyses. For GSVA analysis and visualization, we employed the Python package gseapy (RRID:SCR_025803)[80]. The gp.prerank function was utilized for GSVA analysis on specified gene sets, with parameters configured as min_size = 5, max_size = 1000, and

permutation_num = 1000 to ensure robust statistical interpretation.

For GSEA analysis and subsequent visualization, the raw expression matrix from spatial transcriptomics data was first converted into a Seurat[81] object. Normalization was performed using the NormalizeData function. Subsequent GSEA analysis was conducted using the gsva function from the GSVA[38] R package, with kcdf set to 'Gaussian' to assess the enrichment of gene sets in the context of our spatial transcriptomics data.

Assessment of immune scoring in spatial transcriptomics

To evaluate the proportion of immune cells in each section, we utilized the R package estimate[82]. Initially, gene count matrices for each section were aggregated to create pseudobulk data. This pseudobulk data was normalized using counts per million (CPM) normalization followed by log transformation. Immune cell proportions were then assessed using the estimateScore function, specifying the platform="affymetrix" parameter.

Calculation of SNV spatial correlations and construction of SNV groups

Due to the abundance of SNV features and the proximity of some SNVs on the genome, we implemented a sliding window approach to aggregate SNVs. SNVs were consolidated into windows of 100,000 base pairs each. We hypothesized that the co-occurrence of SNVs in space may indicate alterations in certain biological processes. This phenomenon of co-occurrence makes UMI normalization less crucial; therefore, we utilized unique counts of SNVs to represent the expression levels of SNV windows. On the resulting SNV windows counts matrix, distances between spots were computed using the K-nearest neighbors (KNN) method. Additionally, to refine the SNV windows matrix, we applied a Gaussian weighting scheme to attenuate the influence of distant neighbors, enhancing the accuracy and relevance of the spatial genomic data,

$$M_w = W \times M$$

$$W_{ij} = \exp(-\frac{D_{ij}^2}{2\alpha^2})$$

where M represents the SNV windows count matrix, and W is a matrix of weights applied to adjust these counts based on spatial proximity between spots. Each weight W_{ij} is calculated using a Gaussian function of the squared distances between spots, moderated by a scaling parameter α . M_w represents the adjusted SNV windows matrix.

Due to the high sparsity in the SNV windows matrix, we focused on spots representing the top 50% of expression levels for each window. This approach allowed us to calculate correlations specifically among these more informative spots, thereby optimizing our analysis efficiency,

$$W_{corr} = M_w^T \times M_w$$

where W_{corr} represents the spatial correlation between SNV windows, and M_w represents the transposed matrix of adjusted SNV windows.

After isolating the top 50% of the most correlated windows for each SNV window, we refined the correlation matrix using Gaussian decay. A directed graph was then constructed, with nodes representing the SNV windows and edge weights derived from the adjusted correlations. Then, we applied the Leiden algorithm to cluster SNVs, setting the resolution parameter at approximately 5 to ensure distinct SNV groups were identified. This approach was chosen to preserve groups of SNVs with strong internal correlations. Finally, the UMI count for each spot is used to normalize the SNV Group counts to eliminate the effects of sequencing depth.

Identification of representative genes in SNV windows

Since SNV windows are generated by integrating SNVs, extracting representative genes from SNV windows involves focusing on the gene locations of all SNVs within these windows. We disregard those SNV windows that contain only intergenic SNVs. When all SNVs in a window are located within the same gene, that gene is designated as the representative gene. If an SNV window encompasses multiple genes, we select the gene whose expression pattern aligns with that of the entire SNV window as the representative gene.

Calculation of moran's I

Moran's I was calculated using Squidpy's (RRID:SCR_026157)[83] spatial_neighbors and spatial_autocorr functions to assess the spatial autocorrelation of SNV groups and individual SNVs

TAM signature

To calculate the spatial signature of TAMs, we selected *CD68*, *CD14*, *MMP2*, *MMP9*, *VTCN1*, *CD163*, and *MRC1* as the gene set for TAMs. The scores were computed using the sc.tl.score_genes function in Scanpy.

Cell communication in spatial transcriptomics

COMMOT[84] was utilized to analyze and visualize cell communication within spatial transcriptomics data. Initially, the human-specific cell communication database from CellChat was selected using the ligand_receptor_database function. Potential ligand-receptor pairs were filtered using the filter_lr_database function with a minimum cell percentage threshold set at 0.01. Subsequent analysis of spatial cell communication was conducted through the spatial_communication function, applying parameters such as dis_thr=500 for the distance threshold, heteromeric=True to consider heteromeric complexes, and pathway_sum=True to aggregate signals by pathway.

Neoantigen prediction

We extracted nonsynonymous SNVs annotated by ANNOVAR to construct a potential mutant peptide database. Utilizing amino acid mutation annotations provided by ANNOVAR, we extracted 15 amino acids surrounding each mutated site from the CDS reference matching the reference genome (GCF_000001405.38_GRCh38.p12). A sliding window approach generated potential mutant peptides ranging from 8 to 15 amino acids. Using a Python script, we obtained all peptides of 8-15 amino acids in length from the reference CDS and peptides from the HLA Ligand Atlas database[43] to filter epitopes derived from normal proteins. Concurrently, we employed IEDB[44], MHC Motif Atlas[45], and dbPepNeo[46] to identify known peptides presented by HLAs. Additionally, the affinity between neoantigens and HLA is predicted using T1K with default parameters.

Availability of source code and requirements

Project name: SpatialSNV

Project home page: https://github.com/YoungLi88/SpatialSNV

Operating system(s): Linux

Programming language: Python

Other requirements: Python 3.8 or higher

License: MIT License

RRID: SCR_026221

Data Availability

Public spatial transcriptomics data utilized for analysis on the Visium platform were sourced from the Gene Expression Omnibus (GEO, accession GSE181254). Spatial transcriptomics and DNA data on the Slide-seq platform were obtained from the Sequence Read Archive (SRA, accession PRJNA768453). Liver cancer spatial transcriptomics data on the Stereo-seq platform were acquired from the China National GeneBank (CNGB) Sequence Archive (CNSA, accession code: CNP0002199). The spatial transcriptomics data of colorectal cancer obtained through Stereo-seq were sourced from the Genome Sequence Archive (GSA, accession number: PRJCA020107) and the China National GeneBank DataBase (CNSA, accession code: CNP0002432), while the scRNA-seq data were derived from GSE200997. Other data further supporting this work are openly available in the GigaScience database, GigaDB [85].

Abbreviations

CNVs: copy number variants; COAD: colon adenocarcinoma; CRC: colorectal cancer; DCIS: ductal carcinoma in situ; GO: Gene Ontology; HLAs: human leukocyte antigens; LIHC: liver hepatocellular carcinoma; SNVs: single nucleotide variants; SV: structural variation; UMI: unique molecular identifiers; UTR: untranslated region.

Declarations

Not applicable.

Consent for publication

Not applicable.

Competing interests

The author(s) declare that they have no competing interests.

Funding

This research received no specific grant from any funding agency in the public, commercial, or not-for-profit sectors.

Authors' contributions

Y. Li conceived the idea. Y. Li supervised the work with the help of Y. Feng and X. Dong. Y. Li collected the public data with the help of the Y. Li, F. Zhu and X. Li. Y. Liu performed the data analysis with the help of Y. Li, F. Zhu and X. Guan. Y. Liu and Y. Li wrote the manuscript with the help of Y. Feng and X. Dong.

Acknowledgements

We would like to thank DCS Cloud (https://cloud.stomics.tech/) for providing the computational resources and software support necessary for this study. This work was jointly supported by the Zhejiang Key Laboratory of Spatial Omics.

References

1. Hanahan D and Weinberg RA. Hallmarks of cancer: the next generation. Cell. 2011;144 5:646-74. doi:10.1016/j.cell.2011.02.013.

2. Human Genome Structural Variation Working G, Eichler EE, Nickerson DA, Altshuler D, Bowcock AM, Brooks LD, et al. Completing the map of human genetic variation. Nature. 2007;447 7141:161-5. doi:10.1038/447161a.

3. Macintyre G, Ylstra B and Brenton JD. Sequencing Structural Variants in Cancer for Precision Therapeutics. Trends Genet. 2016;32 9:530-42. doi:10.1016/j.tig.2016.07.002.

4. Rehm HL. Disease-targeted sequencing: a cornerstone in the clinic. Nat Rev Genet. 2013;14 4:295-300. doi:10.1038/nrg3463.

5. Li G, Yang Z, Wu D, Liu S, Li X, Li T, et al. Evolution under Spatially Heterogeneous Selection in Solid Tumors. Molecular Biology and Evolution. 2022;39 1:msab335. doi:10.1093/molbev/msab335.

6. Liu F, Zhang Y, Zhang L, Li Z, Fang Q, Gao R, et al. Systematic comparative analysis of single-nucleotide variant detection methods from single-cell RNA sequencing data. Genome Biology. 2019;20 1:242. doi:10.1186/s13059-019-1863-4.

7. Erickson A, He M, Berglund E, Marklund M, Mirzazadeh R, Schultz N, et al. Spatially resolved clonal copy number alterations in benign and malignant tissue. Nature. 2022;608 7922:360-7. doi:10.1038/s41586-022-05023-2.

8. Arora R, Cao C, Kumar M, Sinha S, Chanda A, McNeil R, et al. Spatial transcriptomics reveals distinct and conserved tumor core and edge architectures that predict survival and targeted therapy response. Nature Communications. 2023;14 1:5029. doi:10.1038/s41467-023-40271-4.

9. Zhao T, Chiang ZD, Morriss JW, LaFave LM, Murray EM, Del Priore I, et al. Spatial genomics enables multi-modal study of clonal heterogeneity in tissues. Nature. 2022;601 7891:85-91. doi:10.1038/s41586-021-04217-4.

10. Lomakin A, Svedlund J, Strell C, Gataric M, Shmatko A, Rukhovich G, et al. Spatial genomics maps the structure, nature and evolution of cancer clones. Nature. 2022;611 7936:594-602. doi:10.1038/s41586-022-05425-2.

11. Chen L, Chang D, Tandukar B, Deivendran D, Pozniak J, Cruz-Pacheco N, et al. STmut: a framework for visualizing somatic alterations in spatial transcriptomics data of cancer. Genome Biology. 2023;24 1:273. doi:10.1186/s13059-023-03121-6.

12. Shafighi S, Geras A, Jurzysta B, Sahaf Naeini A, Filipiuk I, Ra Czkowska A, et al. Integrative spatial and genomic analysis of tumor heterogeneity with Tumoroscope. Nat Commun. 2024;15 1:9343. doi:10.1038/s41467-024-53374-3.

13. Mo C-K, Liu J, Chen S, Storrs E, Targino da Costa ALN, Houston A, et al. Tumour evolution and microenvironment interactions in 2D and 3D space. Nature. 2024;634 8036:1178-86. doi:10.1038/s41586-024-08087-4.

14. Ji AL, Rubin AJ, Thrane K, Jiang S, Reynolds DL, Meyers RM, et al. Multimodal Analysis of Composition and Spatial Architecture in Human Squamous Cell Carcinoma. Cell. 2020;182 2:497-514.e22. doi:https://doi.org/10.1016/j.cell.2020.05.039.

15. Wu L, Yan J, Bai Y, Chen F, Zou X, Xu J, et al. An invasive zone in human liver cancer identified by Stereo-seq promotes hepatocyte–tumor cell crosstalk, local immunosuppression and tumor progression. Cell Research. 2023;33 8:585-603. doi:10.1038/s41422-023-00831-1.

16. Van der Auwera GA and O'Connor BD. Genomics in the cloud: using Docker, GATK, and WDL in Terra. O'Reilly Media; 2020.

17. Zhang R, Feng Y, Ma W, Guo Y, Luo M, Li Y, et al. Spatial transcriptome unveils a discontinuous inflammatory pattern in proficient mismatch repair colorectal adenocarcinoma. Fundamental Research. 2023;3 4:640-6. doi:https://doi.org/10.1016/j.fmre.2022.01.036.

18. Feng Y, Ma W, Zang Y, Guo Y, Li Y, Zhang Y, et al. Spatially organized tumor-stroma boundary determines the efficacy of immunotherapy in colorectal cancer patients. Nature Communications. 2024;15 1:10259. doi:10.1038/s41467-024-54710-3.

19. Wei R, He S, Bai S, Sei E, Hu M, Thompson A, et al. Spatial charting of single-cell transcriptomes in tissues. Nat Biotechnol. 2022;40 8:1190-9. doi:10.1038/s41587-022-01233-1.

20. Rodriques SG, Stickels RR, Goeva A, Martin CA, Murray E, Vanderburg CR, et al. Slide-seq: A scalable technology for measuring genome-wide expression at high spatial resolution. Science. 2019;363 6434:1463-7. doi:doi:10.1126/science.aaw1219.

21. Vaiopoulos AG, Kostakis ID, Athanasoula K and Papavassiliou AG. Targeting transcription factor corepressors in tumor cells. Cell Mol Life Sci. 2012;69 11:1745-53. doi:10.1007/s00018-012-0986-5.

22. Mahmood M, Liu EM, Shergold AL, Tolla E, Tait-Mulder J, Huerta-Uribe A, et al. Mitochondrial DNA mutations drive aerobic glycolysis to enhance checkpoint blockade response in melanoma. Nature Cancer. 2024;5 4:659-72. doi:10.1038/s43018-023-00721-w.

23. Bianco G, Coto-Llerena M, Gallon J, Kancherla V, Taha-Mehlitz S, Marinucci M, et al. GATA3 and MDM2 are synthetic lethal in estrogen receptor-positive breast cancers. Communications Biology. 2022;5 1:373. doi:10.1038/s42003-022-03296-x.

24. Robinson DR, Wu YM, Vats P, Su F, Lonigro RJ, Cao X, et al. Activating ESR1 mutations in hormone-resistant metastatic breast cancer. Nat Genet. 2013;45 12:1446-51. doi:10.1038/ng.2823.

25. Subramanian A, Tamayo P, Mootha VK, Mukherjee S, Ebert BL, Gillette MA, et al. Gene set enrichment analysis: a knowledge-based approach for interpreting genome-wide expression profiles. Proc Natl Acad Sci U S A. 2005;102 43:15545-50. doi:10.1073/pnas.0506580102.

26. Dhanasekaran R, Deutzmann A, Mahauad-Fernandez WD, Hansen AS, Gouw AM and Felsher DW. The MYC oncogene - the grand orchestrator of cancer growth and immune evasion. Nat Rev Clin Oncol. 2022;19 1:23-36. doi:10.1038/s41571-021-00549-2.

27. Hanahan D. Hallmarks of Cancer: New Dimensions. Cancer Discov. 2022;12 1:31-46. doi:10.1158/2159-8290.Cd-21-1059.

28. Huang L, Guo Z, Wang F and Fu L. KRAS mutation: from undruggable to druggable in cancer. Signal Transduction and Targeted Therapy. 2021;6 1:386. doi:10.1038/s41392-021-00780-4.

29. Qiu X, Mao Q, Tang Y, Wang L, Chawla R, Pliner HA, et al. Reversed graph embedding resolves complex single-cell trajectories. Nature Methods. 2017;14 10:979-82. doi:10.1038/nmeth.4402.

30. Aran D, Camarda R, Odegaard J, Paik H, Oskotsky B, Krings G, et al. Comprehensive analysis of normal adjacent to tumor transcriptomes. Nat Commun. 2017;8 1:1077. doi:10.1038/s41467-017-01027-z.

31. Tai IT and Tang MJ. SPARC in cancer biology: Its role in cancer progression and potential for therapy. Drug Resistance Updates. 2008;11 6:231-46. doi:https://doi.org/10.1016/j.drup.2008.08.005.

32. Lazaratos AM, Annis MG and Siegel PM. GPNMB: a potent inducer of immunosuppression in cancer. Oncogene. 2022;41 41:4573-90. doi:10.1038/s41388-022-02443-2.

33. Labani-Motlagh A, Ashja-Mahdavi M and Loskog A. The Tumor Microenvironment: A Milieu Hindering and Obstructing Antitumor Immune Responses. Front Immunol. 2020;11:940. doi:10.3389/fimmu.2020.00940.

34. Zhao H, Wu L, Yan G, Chen Y, Zhou M, Wu Y, et al. Inflammation and tumor progression: signaling pathways and targeted intervention. Signal Transduction and Targeted Therapy. 2021;6 1:263. doi:10.1038/s41392-021-00658-5.

35. Wu Q, You L, Nepovimova E, Heger Z, Wu W, Kuca K, et al. Hypoxia-inducible factors: master regulators of hypoxic tumor immune escape. J Hematol Oncol. 2022;15 1:77. doi:10.1186/s13045-022-01292-6.

36. Zhao Y, Fu X, Lopez JI, Rowan A, Au L, Fendler A, et al. Selection of metastasis competent subclones in the tumour interior. Nat Ecol Evol. 2021;5 7:1033-45. doi:10.1038/s41559-021-01456-6.

37. Dongre A and Weinberg RA. New insights into the mechanisms of epithelial-mesenchymal transition and implications for cancer. Nat Rev Mol Cell Biol. 2019;20 2:69-84. doi:10.1038/s41580-018-0080-4.

38. Hänzelmann S, Castelo R and Guinney J. GSVA: gene set variation analysis for microarray and RNA-Seq data. BMC Bioinformatics. 2013;14 1:7. doi:10.1186/1471-2105-14-7.

39. Yu C-F, Chen F-H, Lu M-H, Hong J-H and Chiang C-S. Dual roles of tumour cells-derived matrix metalloproteinase 2 on brain tumour growth and invasion. British Journal of Cancer. 2017;117 12:1828-36. doi:10.1038/bjc.2017.362.

40. Jiang H and Li H. Prognostic values of tumoral MMP2 and MMP9 overexpression in breast cancer: a systematic review and meta-analysis. BMC Cancer. 2021;21 1:149. doi:10.1186/s12885-021-07860-2.

41. Wei S, Liu W, Xu M, Qin H, Liu C, Zhang R, et al. Cathepsin F and Fibulin-1 as novel diagnostic biomarkers for brain metastasis of non-small cell lung cancer. British Journal of Cancer. 2022;126 12:1795-805. doi:10.1038/s41416-022-01744-3.

42. Huang Y, Xu Z, Holtappels G, Shen Y, Van Zele T, Wen W, et al. MZB1-expressing cells are essential for local immunoglobulin production in chronic rhinosinusitis with nasal polyps. Ann Allergy Asthma Immunol. 2024;132 2:198-207.e14. doi:10.1016/j.anai.2023.10.008.

43. Marcu A, Bichmann L, Kuchenbecker L, Kowalewski DJ, Freudenmann LK, Backert L, et al. HLA Ligand Atlas: a benign reference of HLA-presented peptides to improve T-cell-based cancer immunotherapy. J Immunother Cancer. 2021;9 4 doi:10.1136/jitc-2020-002071.

44. Vita R, Mahajan S, Overton JA, Dhanda SK, Martini S, Cantrell JR, et al. The Immune Epitope Database (IEDB): 2018 update. Nucleic Acids Research. 2018;47 D1:D339-D43. doi:10.1093/nar/gky1006.

45. Tadros DM, Eggenschwiler S, Racle J and Gfeller D. The MHC Motif Atlas: a database of MHC binding specificities and ligands. Nucleic Acids Research. 2022;51 D1:D428-D37. doi:10.1093/nar/gkac965.

46. Tan X, Li D, Huang P, Jian X, Wan H, Wang G, et al. dbPepNeo: a manually curated database for human tumor neoantigen peptides. Database (Oxford). 2020;2020 doi:10.1093/database/baaa004.

47. Zhu G, Pei L, Xia H, Tang Q and Bi F. Role of oncogenic KRAS in the prognosis, diagnosis and treatment of colorectal cancer. Mol Cancer. 2021;20 1:143. doi:10.1186/s12943-021-01441-4.

48. Lu D, Chen Y, Jiang M, Wang J, Li Y, Ma K, et al. KRAS G12V neoantigen specific T cell receptor for adoptive T cell therapy against tumors. Nat Commun. 2023;14 1:6389. doi:10.1038/s41467-023-42010-1.

49. Poole A, Karuppiah V, Hartt A, Haidar JN, Moureau S, Dobrzycki T, et al. Therapeutic high affinity T cell receptor targeting a KRAS(G12D) cancer neoantigen. Nat Commun. 2022;13 1:5333. doi:10.1038/s41467-022-32811-1.

50. Su W, Feng B, Hu L, Guo X and Yu M. MUC3A promotes the progression of colorectal cancer through the PI3K/Akt/mTOR pathway. BMC Cancer. 2022;22 1:602. doi:10.1186/s12885-022-09709-8.

51. Sotoudeh M, Shirvani SI, Merat S, Ahmadbeigi N and Naderi M. MSLN (Mesothelin), ANTXR1 (TEM8), and MUC3A are the potent antigenic targets for CAR T cell therapy of gastric adenocarcinoma. J Cell Biochem. 2019;120 4:5010-7. doi:10.1002/jcb.27776.

52. Song L, Bai G, Liu XS, Li B and Li H. Efficient and accurate KIR and HLA genotyping with massively parallel sequencing data. Genome Res. 2023;33 6:923-31. doi:10.1101/gr.277585.122.

53. Chu Y, Zhang Y, Wang Q, Zhang L, Wang X, Wang Y, et al. A transformer-based model to predict peptide–HLA class I binding and optimize mutated peptides for vaccine design. Nature Machine Intelligence. 2022;4 3:300-11. doi:10.1038/s42256-022-00459-7.

54. Habel JR, Nguyen AT, Rowntree LC, Szeto C, Mifsud NA, Clemens EB, et al. HLA-A*11:01-restricted CD8+ T cell immunity against influenza A and influenza B viruses in Indigenous and non-Indigenous people. PLoS Pathog. 2022;18 3:e1010337. doi:10.1371/journal.ppat.1010337.

55. Lee H-O, Hong Y, Etlioglu HE, Cho YB, Pomella V, Van den Bosch B, et al. Lineage-dependent gene expression programs influence the immune landscape of colorectal cancer. Nature Genetics. 2020;52 6:594-603. doi:10.1038/s41588-020-0636-z.

56. Cheng M, Jiang Y, Xu J, Mentis AA, Wang S, Zheng H, et al. Spatially resolved transcriptomics: a comprehensive review of their technological advances, applications, and challenges. J Genet Genomics. 2023;50 9:625-40. doi:10.1016/j.jgg.2023.03.011.

57. Schnepp PM, Chen M, Keller ET and Zhou X. SNV identification from single-cell RNA sequencing data. Human Molecular Genetics. 2019;28 21:3569-83. doi:10.1093/hmg/ddz207.

58. Bai Z, Zhang D, Gao Y, Tao B, Zhang D, Bao S, et al. Spatially exploring RNA biology in archival formalin-fixed paraffin-embedded tissues. Cell. 2024;187 23:6760-79.e24. doi:10.1016/j.cell.2024.09.001.

59. Pastushenko I, Brisebarre A, Sifrim A, Fioramonti M, Revenco T, Boumahdi S, et al. Identification of the tumour transition states occurring during EMT. Nature. 2018;556 7702:463-8. doi:10.1038/s41586-018-0040-3.

60. Chen Z, Han F, Du Y, Shi H and Zhou W. Hypoxic microenvironment in cancer: molecular mechanisms and therapeutic interventions. Signal Transduction and Targeted Therapy. 2023;8 1:70. doi:10.1038/s41392-023-01332-8.

61. Martincorena I, Roshan A, Gerstung M, Ellis P, Van Loo P, McLaren S, et al. High burden and pervasive positive selection of somatic mutations in normal human skin. Science. 2015;348 6237:880-6. doi:10.1126/science.aaa6806.

62. Samuel T, Rapic S, O'Brien C, Edson M, Zhong Y and DaCosta RS. Quantitative intravital imaging for real-time monitoring of pancreatic tumor cell hypoxia and stroma in an orthotopic mouse model. Science Advances. 9 23:eade8672. doi:10.1126/sciadv.ade8672.

63. Xie N, Shen G, Gao W, Huang Z, Huang C and Fu L. Neoantigens: promising targets for cancer therapy. Signal Transduction and Targeted Therapy. 2023;8 1:9. doi:10.1038/s41392-022-01270-x.

64. Stephens PJ, Greenman CD, Fu B, Yang F, Bignell GR, Mudie LJ, et al. Massive genomic rearrangement acquired in a single catastrophic event during cancer development. Cell. 2011;144 1:27-40. doi:10.1016/j.cell.2010.11.055.

65. Besedina E and Supek F. Copy number losses of oncogenes and gains of tumor suppressor genes generate common driver mutations. Nature Communications. 2024;15 1:6139. doi:10.1038/s41467-024-50552-1.

66. Markowska M, Cąkała T, Miasojedow B, Aybey B, Juraeva D, Mazur J, et al. CONET: copy number event tree model of evolutionary tumor history for single-cell data. Genome Biology. 2022;23 1:128. doi:10.1186/s13059-022-02693-z.

67. Yuan K, Sakoparnig T, Markowetz F and Beerenwinkel N. BitPhylogeny: a probabilistic framework for reconstructing intra-tumor phylogenies. Genome Biology. 2015;16 1:36. doi:10.1186/s13059-015-0592-6.

68. Shi Y, Jing B and Xi R. Comprehensive analysis of neoantigens derived from structural variation across whole genomes from 2528 tumors. Genome Biol. 2023;24 1:169. doi:10.1186/s13059-023-03005-9.

69. Turajlic S, Litchfield K, Xu H, Rosenthal R, McGranahan N, Reading JL, et al. Insertion-and-deletion-derived tumour-specific neoantigens and the immunogenic phenotype: a pan-cancer analysis. Lancet Oncol. 2017;18 8:1009-21. doi:10.1016/s1470-2045(17)30516-8.

70. Lin M, Zhang XL, You R, Yang Q, Zou X, Yu K, et al. Neoantigen landscape in metastatic nasopharyngeal carcinoma. Theranostics. 2021;11 13:6427-44. doi:10.7150/thno.53229.

71. Gong C, Li S, Wang L, Zhao F, Fang S, Yuan D, et al. SAW: an efficient and accurate data analysis workflow for Stereo-seq spatial transcriptomics. GigaByte. 2024;2024:gigabyte111. doi:10.46471/gigabyte.111.

72. Wolf FA, Angerer P and Theis FJ. SCANPY: large-scale single-cell gene expression data analysis. Genome Biol. 2018;19 1:15. doi:10.1186/s13059-017-1382-0.

73. Ramirez F, Ryan DP, Gruning B, Bhardwaj V, Kilpert F, Richter AS, et al. deepTools2: a next generation web server for deep-sequencing data analysis. Nucleic Acids Res. 2016;44 W1:W160-5. doi:10.1093/nar/gkw257.

74. Merchant N, Lyons E, Goff S, Vaughn M, Ware D, Micklos D, et al. The iPlant Collaborative: Cyberinfrastructure for Enabling Data to Discovery for the Life Sciences. PLoS Biol. 2016;14 1:e1002342. doi:10.1371/journal.pbio.1002342.

75. Kent WJ, Sugnet CW, Furey TS, Roskin KM, Pringle TH, Zahler AM, et al. The human genome browser at UCSC. Genome Res. 2002;12 6:996-1006. doi:10.1101/gr.229102.

76. Li H, Handsaker B, Wysoker A, Fennell T, Ruan J, Homer N, et al. The Sequence Alignment/Map format and SAMtools. Bioinformatics. 2009;25 16:2078-9. doi:10.1093/bioinformatics/btp352.

77. Wang K, Li M and Hakonarson H. ANNOVAR: functional annotation of genetic variants from high-throughput sequencing data. Nucleic Acids Res. 2010;38 16:e164. doi:10.1093/nar/gkq603.

78. Yu G, Wang L-G, Han Y and He Q-Y. clusterProfiler: an R Package for Comparing Biological Themes Among Gene Clusters. OMICS: A Journal of Integrative Biology. 2012;16 5:284-7. doi:10.1089/omi.2011.0118.

79. Liberzon A, Birger C, Thorvaldsdóttir H, Ghandi M, Mesirov JP and Tamayo P. The molecular signatures database hallmark gene set collection. Cell systems. 2015;1 6:417-25.

80. Fang Z, Liu X and Peltz G. GSEApy: a comprehensive package for performing gene set enrichment analysis in Python. Bioinformatics. 2022;39 1 doi:10.1093/bioinformatics/btac757.

81. Hao Y, Stuart T, Kowalski MH, Choudhary S, Hoffman P, Hartman A, et al. Dictionary learning for integrative, multimodal and scalable single-cell analysis. Nature Biotechnology. 2024;42 2:293-304. doi:10.1038/s41587-023-01767-y.

82. Scire J, Huisman JS, Grosu A, Angst DC, Lison A, Li J, et al. estimateR: an R package to estimate and monitor the effective reproductive number. BMC Bioinformatics. 2023;24 1:310. doi:10.1186/s12859-023-05428-4.

83. Palla G, Spitzer H, Klein M, Fischer D, Schaar AC, Kuemmerle LB, et al. Squidpy: a scalable framework for spatial omics analysis. Nature Methods. 2022;19 2:171-8. doi:10.1038/s41592-021-01358-2.

84. Cang Z, Zhao Y, Almet AA, Stabell A, Ramos R, Plikus MV, et al. Screening cell–cell communication in spatial transcriptomics via collective optimal transport. Nature Methods. 2023;20 2:218-28. doi:10.1038/s41592-022-01728-4.

85. Liu Y, Zhu F, Li X, Guan X, Hou Y, Feng Y, et al. Supporting data for "SpatialSNV: A Novel Method for Identifying and Analyzing Spatially Resolved SNVs in Tumor Microenvironments". 2025. http://gigadb.org/dataset/102711.

Figure legend

Figure 1. SpatialSNV exhibits spatial distribution mutations by calling SNVs from spatial transcriptomics.

(A) Schematic depicting the methodology for detecting SNVs from spatial transcriptomics data. **(B)** Frequency histogram illustrating the distribution of shared SNVs among spatial spots, with representative sections selected for each platform: CRC-P19-T (Stereo-seq), DCIS1 (Visium), and primary human colorectal cancer (Slide-RNA-seq and Slide-DNA-seq). **(C)** Scatter plot showing the correlation between unique reads (excluding duplicates) from spatial transcriptomics and the number of detected effective SNVs. **(D)** Stacked percentage bar chart showing genomic regions containing effective SNVs. Circular markers denote the platforms of the samples, and square markers specify the cancer types. **(E)** Bar plot detailing the proportion of effective SNVs within gene body regions. **(F)** UpSet plots demonstrating the intersections of effective SNVs among different sections. Yellow bar represents samples from the same patient, while brown and purple bars correspond to samples from liver cancer or colorectal cancer, respectively. Pink bars indicate SNVs that are common in all patients. Sample IDs are colored according to the same patient. **(G)** Bar plots illustrating GO Term enrichment for Molecular Function associated with effective SNVs, as detailed in panel D. The chord diagram illustrates the genes involved in key GO pathways, highlighting their connections and functional relevance. **(H)** Hematoxylin and eosin (H&E) staining (left) and spatial visualization(right) of normalized SNV counts across representative samples from various platforms.

Figure 2. Tumorgenesis associated SNVs inferred from spatial transcriptomics data.

(A) Spatial visualization of the region clustered by spatial transcriptomics. **(B)** Heatmaps of the primary markers of the cluster region as shown in panel a, with expression counts normalized by z-score. **(C)** Scatter plots demonstrating the mutation frequency of genes alongside the average occurrence of corresponding SNVs across spatial spots. **(D)** Venn diagrams showing the overlap of mutated genes from spatial transcriptomics with those identified in related cancer types in TCGA. **(E)** Spatial visualization representing the distribution of driver genes *GATA3* and *ESR1*, along with their corresponding representative SNVs. **(F)** Bar plot illustrating the correlation between *GATA3* expression and its representative SNVs with the prevalence of tumor regions, with statistical significance assessed using the chi-square test. **(G)** Dot plot highlighting the expression levels of driver genes *GATA3* and *ESR1* and their corresponding SNVs in different regions of DCIS1. **(H)** Bar plots demonstrating correlations between various pathways scores and normalized SNV counts per spot. Red bars indicate positive correlations, while blue bars denote negative correlations.

Figure 3. SpatialSNV traces the mutation history of different tumor subclones.

(A) Top: Spatial visualization illustrating the distribution of the tumor subclones. Bottom: UMAP projection of the tumor subclones. **(B)** Heatmap generated by inferCNV displaying inferred CNA profiles for three tumor subclones. **(C)** Heatmap showing the normalized SNV count of specific SNVs within three tumor subclones at the pseudo-bulk level. **(D)** Spatial visualization of normalized SNV counts and gene expression. **(E)** Violin plot showing the expression levels of specific SNVs and genes within the tumor subclones. **(F)** Spatial spot trajectory generated by Spatial SNV analysis, depicting the pseudotime and evolutionary trajectories of three tumor subclones. **(G)** Spatial spot trajectory showing the accumulation of SNV types along the evolutionary trajectories. **(H)** Violin plot displaying the number of SNV types across all spots within three tumor subclones. **(I)** Scatter plot displaying the expression variations of tumor specific SNVs and corresponding genes across different evolutionary branches. **(J)** Spatial visualization of the pseudotime of tumor subclones.

Figure 4. The immune microenvironment shapes mutational pressure in tumor margins.

(A) Spatial visualization of the regions in the Stereo-seq section CRC-P59-T1. **(B)** Violin plots showing the distribution of SNV types across different regions with statistical significance determined by T-tests; **** indicates p-values < 0.01. **(C)** Venn diagram illustrating the differences in nonsynonymous SNVs between distinct regions. **(D)** Heatmap displaying the normalized counts of the top 20 spatial SNVs specific to adjacent margins. SNVs located in intergenic regions are highlighted in blue. **(E)** Spatial visualization showing specific intergenic SNVs from panel D. **(F)** Reads track visualizing the alignment of reads in intergenic regions across different regions. **(G)** GO term analysis of genes affected by intergenic SNVs, categorizing into Biological Process (BP), Cellular Component (CC), and Molecular Function (MF). **(H)** Ridgeline plot illustrating specific pathway scores calculated through GSVA across different regions. **(I)** Scatter plot showing the relationship between normalized SNV count and tumor distance in each spatial spot, with both axes log-transformed. R^2 represents the Pearson correlation coefficient. **(J)** Scatter plot displaying the correlation between the slopes of normalized SNV counts versus tumor distance from panel I and estimate scores. Translucent bands around the regression line indicate the confidence interval for the regression estimate.

Figure 5. Spatially correlated SNVs contribute to shaping tumor microenvironments synergistically.

(A) Schematic depicting the methodology for detecting groups of SNVs. **(B)** Boxplot of the Moran's index for the top 10 SNV groups. Gray dots represent the Moran's index of individual SNVs within each group, while red dots indicate the Moran's index for each SNV group. **(C)** Top: Spatial visualization of SNV Group 1 and SNV Group 2. Bottom: Dot plot of the top 10 SNV groups expression in tumor and adjacent margins. **(D)** GO term analysis of genes within the SNV windows of SNV Group 1 and SNV Group 2. **(E)** Gene Set Variation Analysis of the enriched pathways for genes within SNV windows of SNV Group 2. **(F)** Network graph illustrating the connectivity among SNV windows in SNV Group 2, with points representing SNV windows and line lengths indicating the degree of correlation. **(G)** Spatial visualization of the spatial distribution of highly connected SNV windows (top) and the representative genes contained within these windows (bottom). **(H)** Spatial visualization of the TAM score (up) and the trend of the PTN signaling pathway across the spatial domain (bottom).

Figure 6. SpatialSNV reveals potential neoantigens in tumors.

(A) Schematic illustrating the methodology for constructing mutated peptides. **(B)** Stacked bar chart showing the number of effective SNVs occurring on exons in colorectal cancer samples, with layers representing different patterns of impact on protein-coding caused by SNVs. **(C)** Venn diagram depicting the quantity and shared extent of nonsynonymous SNVs across different samples. **(D)** Heatmap displaying mutated peptides that are supported by the common epitope database and their sharing levels among samples. **(E)** Bar plot showing the detection of genes originating the mutated peptides featured in panel D across different samples. **(F)** Spatial visualization of wild-type and mutated *KRAS* or *S100A11* spots. **(G)** Bar plot illustrating the predicted binding affinity of mutated peptides to different HLA types. **(H)** Heatmap generated by inferCNV depicting the inferred copy number alterations (CNA) profiles for spots exhibiting the chr1_152033685 (S100A11^{L40P}) mutation. **(I)** UMAP projection of the single-cell data from GSE132465. **(J)** Dot plot of the expression of primary markers for tumor and *S100A11* across different clusters. **(K)** Dot plot of the expression of primary markers for tumor and *S100A11* across different samples.

Figure 1

Click here to access/download;Figure;Figure1_r2.pdf

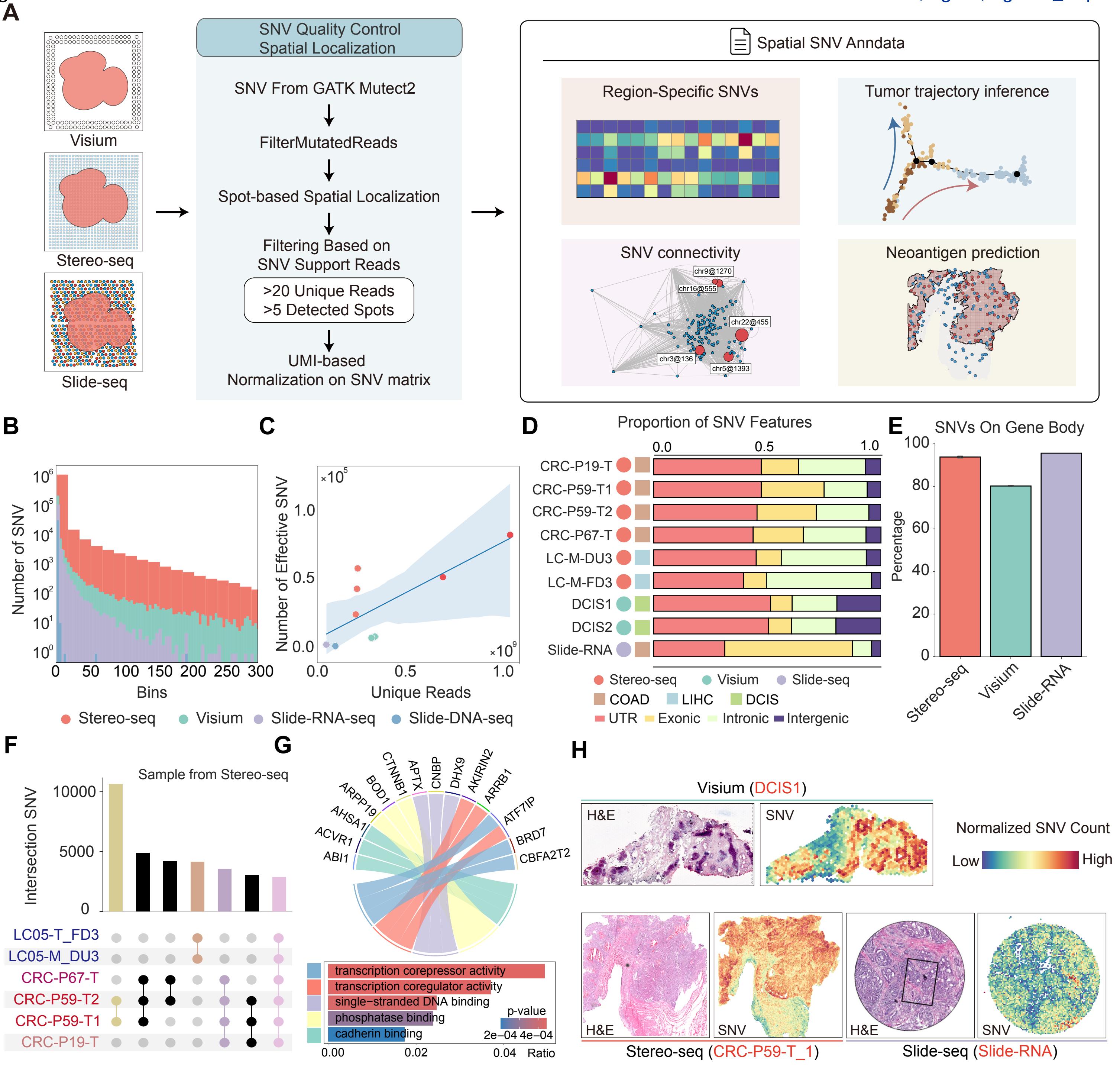

Figure2

Click here to access/download;Figure;Figure2.pdf

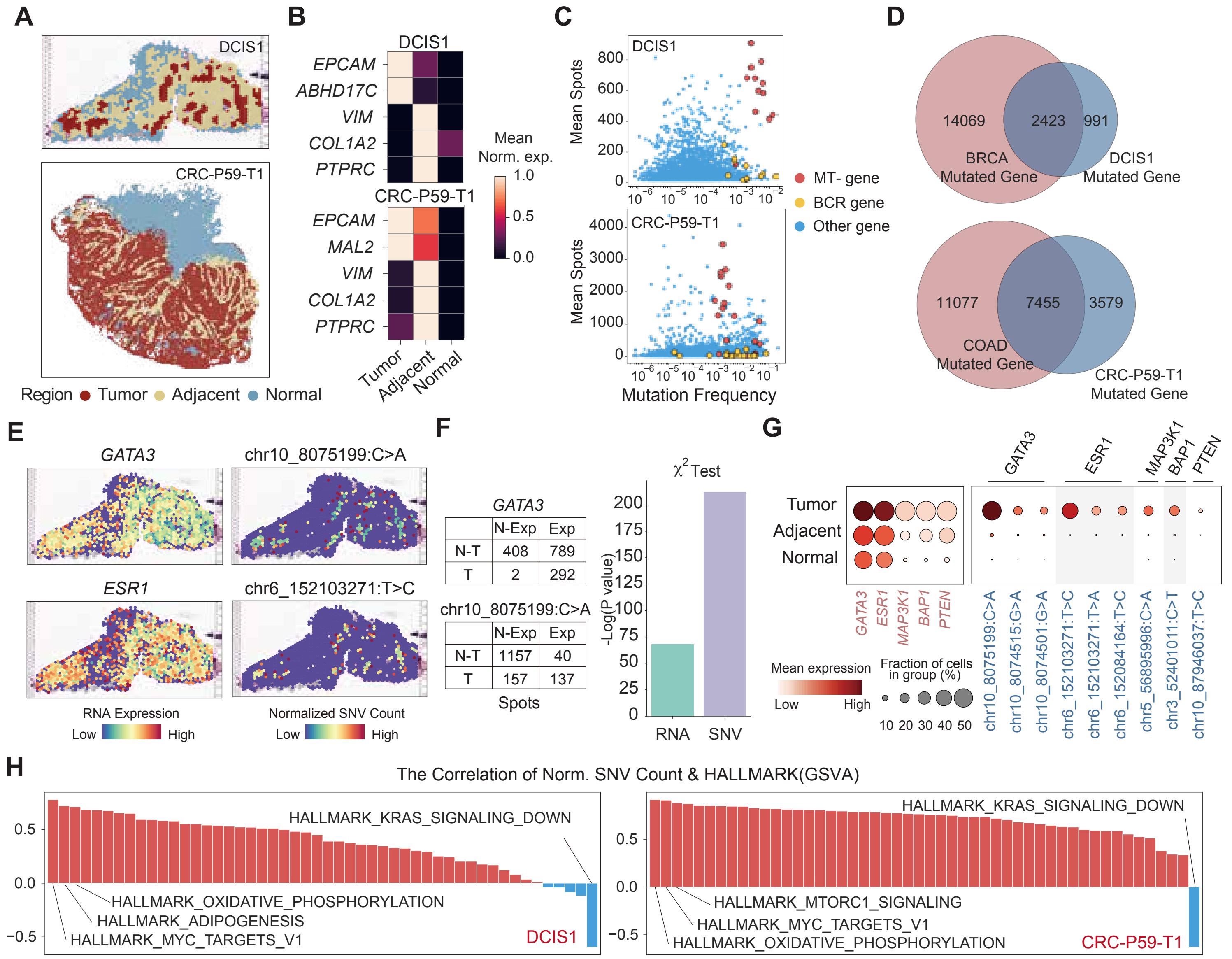

Figure3

Click here to access/download;Figure;Figure3.pdf

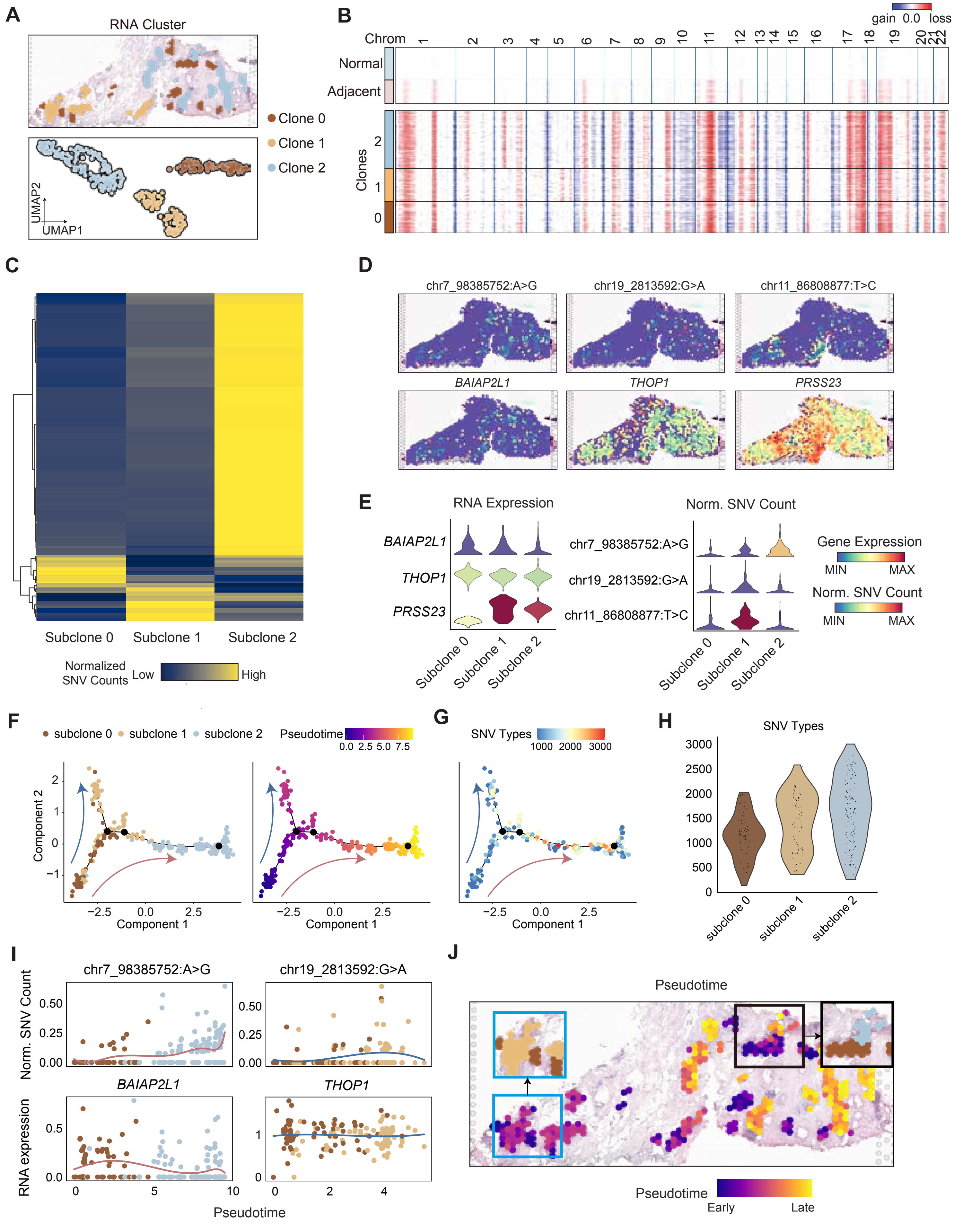

Figure 4

Click here to access/download;Figure;Figure4_r2.pdf

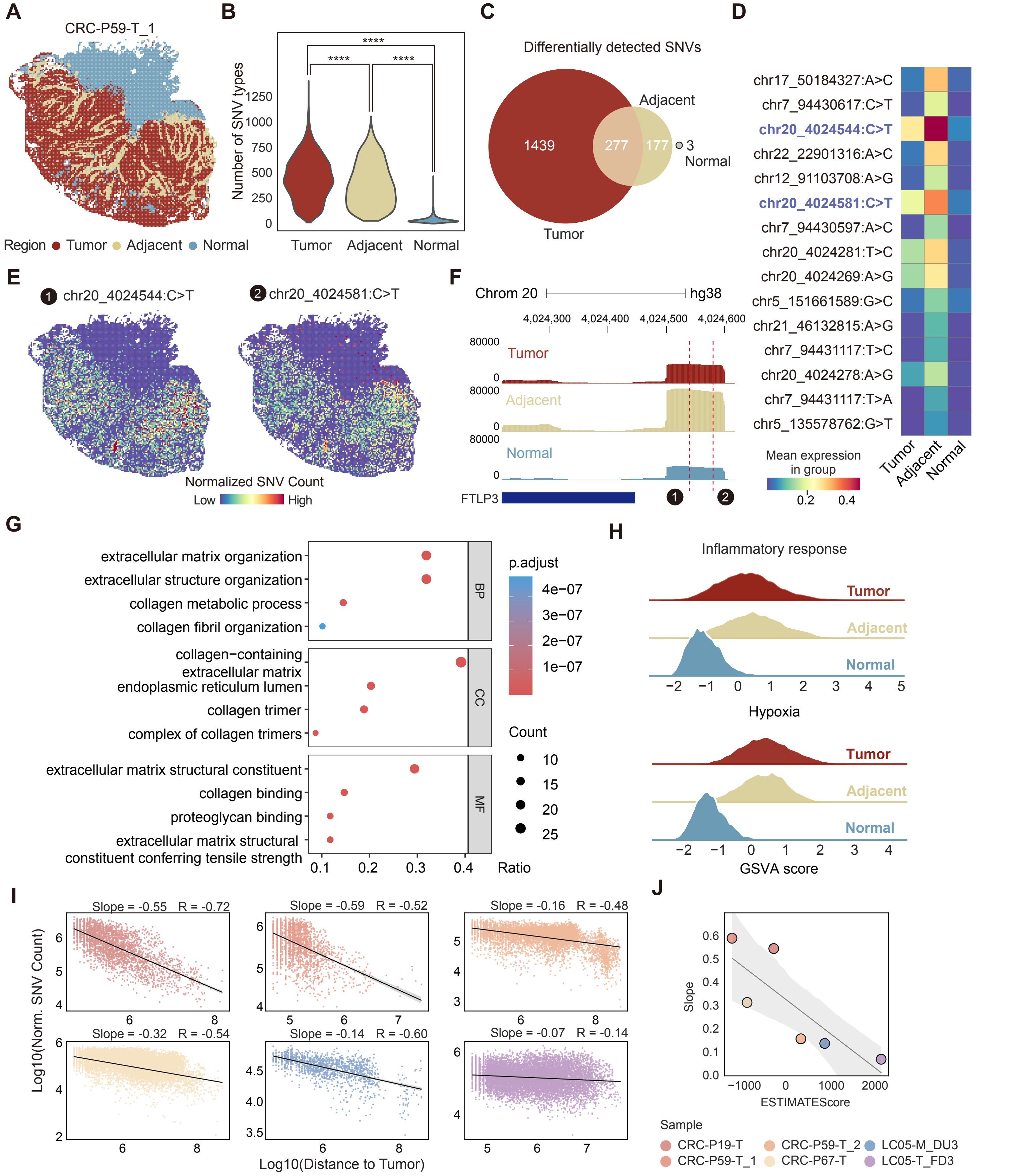

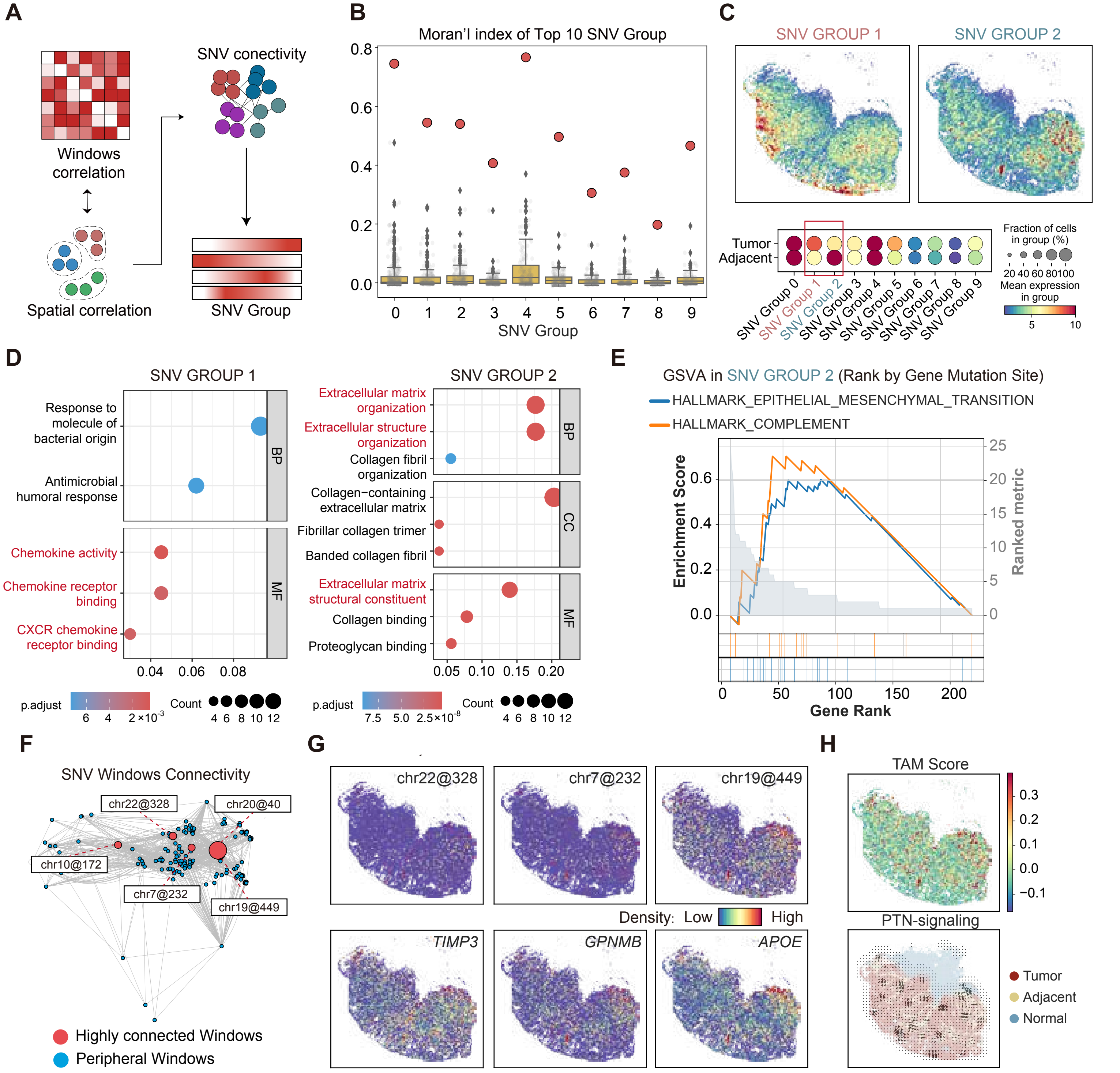
A
Windows correlation
Spatial correlation
SNV conectivity
SNV Group
B
Moran'I index of Top 10 SNV Group
SNV Group
C
SNV GROUP 1
SNV GROUP 2
Tumor
Adjacent
Fraction of cells in group (%)
Mean expression in group
D
SNV GROUP 1
SNV GROUP 2
Response to molecule of bacterial origin
Antimicrobial humoral response
Chemokine activity
Chemokine receptor binding
CXCR chemokine receptor binding
Extracellular matrix organization
Extracellular structure organization
Collagen fibril organization
Collagen−containing extracellular matrix
Fibrillar collagen trimer
Banded collagen fibril
Extracellular matrix structural constituent
Collagen binding
Proteoglycan binding
p.adjust
Count
E
GSVA in SNV GROUP 2 (Rank by Gene Mutation Site)
HALLMARK_EPITHELIAL_MESENCHYMAL_TRANSITION
HALLMARK_COMPLEMENT
Enrichment Score
Ranked metric
Gene Rank
F
SNV Windows Connectivity
chr22@328
chr20@40
chr10@172
chr7@232
chr19@449
Highly connected Windows
Peripheral Windows
G
chr22@328
chr7@232
chr19@449
Density: Low High
TIMP3
GPNMB
APOE
H
TAM Score
PTN-signaling
Tumor
Adjacent
Normal

Figure 6

Click here to access/download;Figure;Figure6_r2.pdf

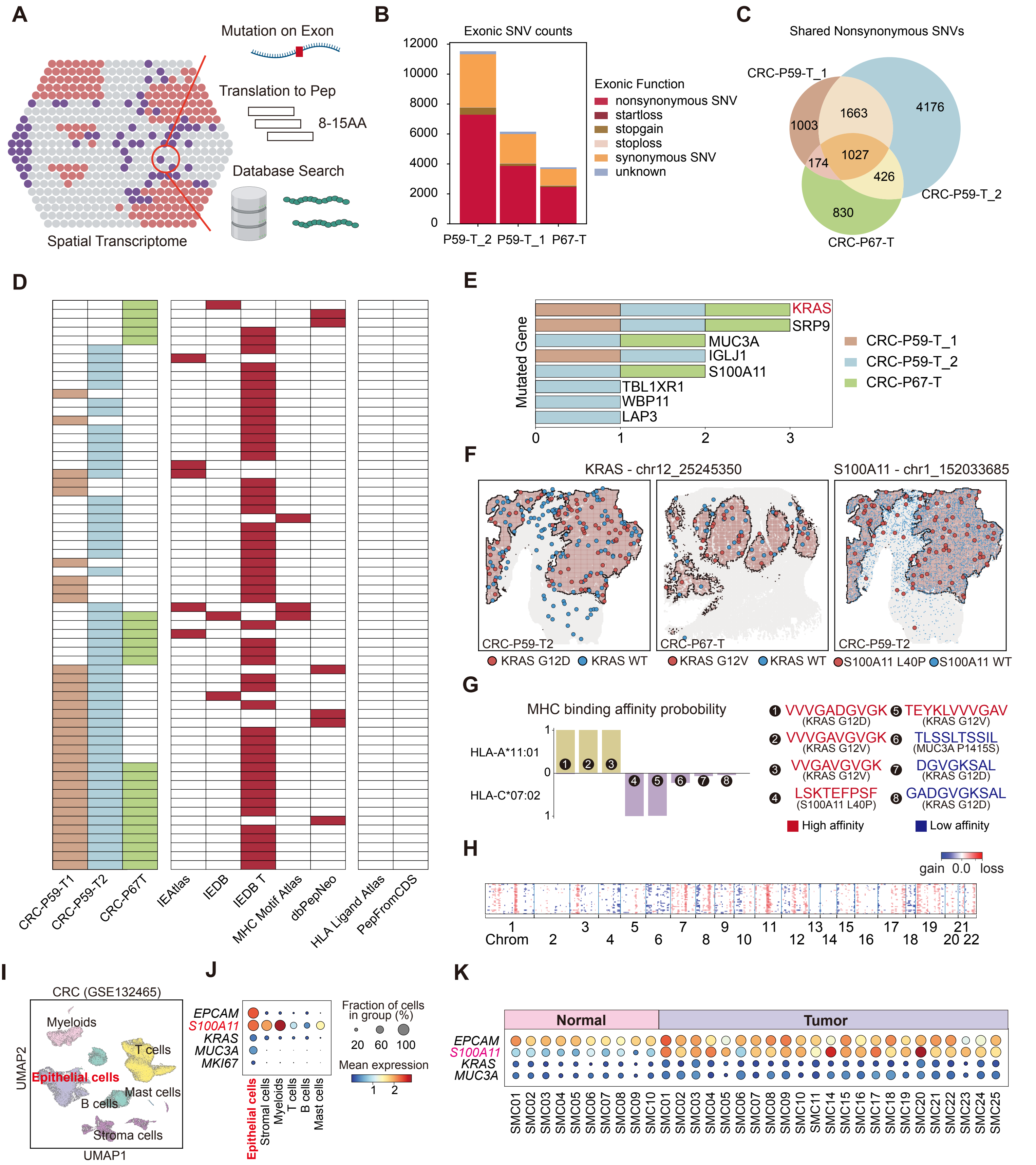

Click here to access/download
Supplementary Material
Supplementary_Files_r3.docx

Cover Letter
Click here to access/download;Personal Cover;Cover_letter_spatialsnv_gigascience.pdf

Dear Editor,

I am pleased to submit our manuscript entitled "***SpatialSNV: A Novel Method for Identifying and Analyzing Spatially Resolved SNVs in Tumor Microenvironments***" for consideration for publication on ***GigaScience***. All the authors listed have approved the manuscript that is enclosed.

Understanding the dynamics of SNVs is crucial for comprehending tumor development. However, the impact of SNVs on tumor microenvironments has remained largely unclear. In our study, we developed SpatialSNV for calling and analyzing effective SNVs from spatial transcriptomics data. SNVs within the tumor context using spatial transcriptomics, revealing critical insights into tumor microenvironments:

1. We developed SpatialSNV to identify effective SNVs from multiple spatial transcriptomics platforms across tumor sections. Our findings indicate that SNVs harbor regional tumor evolutionary traces beyond RNA expression changes.
2. By exploring SNVs in different tumor contexts, we observed increased mutational pressure with the presence of novel SNVs at the tumor margins, which declined distance-dependent from the tumor boundary and were significantly associated with inflammatory and hypoxic microenvironments.
3. SpatialSNV can identify spatially correlated SNV groups exhibiting similar spatial patterns. Specific SNV groups were found to be responsible for the crosstalk between tumors and the immune system at the tumor margins, suggesting that tumor mutations could regulate the surrounding immune microenvironment.
4. SpatialSNV is capable of identifying tumor neoantigens. We observed that critical tumor SNVs exhibit specific spatial patterns within the tissue context. Examination of exonic nonsynonymous SNVs identified $S100A11^{L40P}$ as a tumor region-specific mutation in colorectal cancer (CRC) samples. This mutation may generate neoantigens presented by HLAs, representing a potential therapeutic target.

These findings demonstrate that SpatialSNV can identify spatially resolved SNVs and help to illustrate a more comprehensive view of tumor microenvironments, enhancing our understanding of the spatial heterogeneity of tumor tissues and providing potential therapeutic targets for precision medicine. We believe that our SpaitalSNV will be of great interest to researchers in this field, as it

reveals new insights on the role of SNVs in tumor biology and provides potential avenues for therapeutic intervention.

Thank you for considering our manuscript for publication. We look forward to your response.

Sincerely,

Young Li on behalf of all authors
BGI Research, Hangzhou 310030, China.
Email: liyang13@genomics.cn

revision cover letter

Click here to access/download;Personal Cover;revision_letter.pdf

Dear Editor,

Thank you very much for your email and decision regarding our manuscript titled "***SpatialSNV: A Novel Method for Identifying and Analyzing Spatially Resolved SNVs in Tumor Microenvironments***". We appreciate the opportunity to revise our manuscript and address the reviewers' comments. We have carefully considered all the feedback and made significant revisions to improve the clarity, accuracy, and overall quality of the manuscript. Below, we summarize the key changes made in response to the reviewers' comments:

1. Language and Terminology Refinement:

We have softened and refined several statements throughout the manuscript to avoid overinterpretation or generalization of the results. For example, we replaced terms such as "responsible for" with more accurate language like "associated with" to better reflect the evidence. Additionally, due to the lack of strong evidence and concerns raised by reviewers, we revised "mutational pressure" to "mutational dynamics" to ensure that our conclusions are not overstated and remain well-supported by the data.

2. Sample and Data Clarification:

We have provided additional details about the samples used in the study, including their stage, grade, and mutation status, in Supplementary Figure 1A. We have also clarified the mapping of samples to platforms and improved the consistency of sample labeling across figures.

3. Neoantigen Discovery and Validation:

We have clarified the limitations of our neoantigen predictions and emphasized that these predictions are intended to guide experimental validation rather than serve as definitive conclusions. We have also acknowledged the potential biases introduced by 3' end-based sequencing methods and included this discussion in the manuscript.

4. Figure and Supplementary Material Updates:

We have revised several figures (e.g., Figure 1D, Figure 1H, Supplementary Figure 5C) to improve clarity and address specific reviewer concerns. Additional details and annotations have been added to ensure that the figures are more informative and easier to interpret.

5. Generalization and Overstatement:

We have carefully reviewed the manuscript to ensure that our conclusions are appropriately supported by the data. We have softened broad generalizations and added caveats where necessary, particularly in the Discussion section, to acknowledge the limitations of our study and the need for further validation.

We believe these revisions have significantly strengthened the manuscript and addressed the reviewers' concerns. We are grateful for the opportunity to improve our work and hope that the revised version meets the journal's standards for publication.

Thank you for your time and consideration. Sincerely yours,

Young Li on behalf of all authors
Young Li, Ph.D., BGI Research, Hangzhou 310030, China.
Email: liyang13@genomics.cn

revision detail

Click here to access/download;Personal Cover;revision_detail.pdf

Reviewer #1:

1. This study develops a new computational tool using data from other spatial datasets to make inferences about tumor biology. Further clarification of the samples used in the study and how they were selected is needed. Additional details about those samples would also be useful if available (stage, grade, mutation status, metastatic). In figure 1, the samples used for 1D is unclear.

Reply: We thank the reviewer for pointing this out. We revised Figure 1D and incorporated the sample information as shown in the updated figure. Additionally, we included more detailed sample information in Supplementary Figure 1A. We hope these modifications can address the reviewer's concerns and enhance the overall clarity of the manuscript.

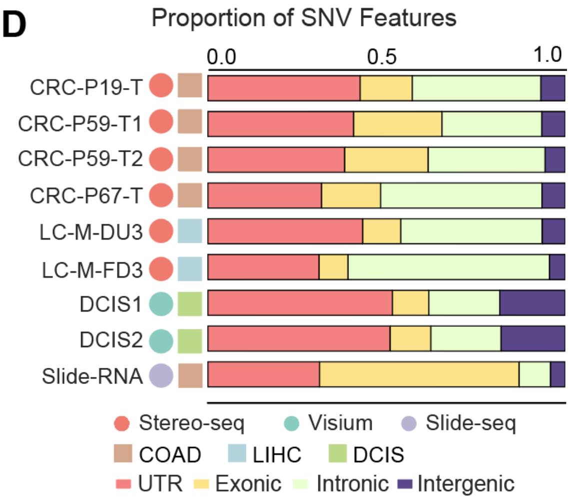

Figure 1D: Stacked percentage bar chart showing genomic regions containing effective SNVs. Circular markers denote the platforms of the samples, and square markers specify the cancer types.

A

Sample Name	Platform	Type	Additional Info	PMID	Sample Name	Platform	Type	Additional Info	PMID
DCIS1	Visium	Ductal carcinoma in situ	ER+/PR-	35314812	CRC-P67-T	Stereo-seq	Colorectal cancer	pMMR	39592630
DCIS2	Visium	Ductal carcinoma in situ	ER+/PR+	35314812	LC05-M_DU3	Stereo-seq	Intrahepatic cholangiocarcinoma	Stage II	37337030
CRC-P19-T	Stereo-seq	Colorectal cancer	pMMR	38933545	LC05-T_FD3	Steroe-seq	Intrahepatic cholangiocarcinoma	Stage II	37337030
CRC-P59-T_1	Stereo-seq	Colorectal cancer	pMMR	39592630	Slide-RNA	Slide-RNA-seq	Colorectal cancer	Stage IIIB	34912115
CRC-P59-T_2	Stereo-seq	Colorectal cancer	pMMR	39592630	Slide-DNA	Slide-DNA-seq	Colorectal cancer	Stage IIIB	34912115

Supplementary Figure 1A: Table presenting the data sources for each sample.

2. The study describes increased mutational boundaries at the tumor periphery, but the data to support this limited. Clearer visualization of these SNVs at the periphery would be useful. Which SNVs are associated with these phenotypes? The inferences in the abstract and text are overstated without additional data

Reply: We sincerely thank the reviewer for raising this important issue. We acknowledge that the concept of mutational pressure at the tumor margins may have been overstated due to insufficient data support. To address this, we have revised the Abstract and Results sections to soften our language regarding mutational pressure. The specific changes are as follows:

1. In the Abstract, we revised:

"**Increased mutational pressure was observed at tumor margins**, with novel SNVs diminishing in a distance-dependent manner from the tumor boundary."

to:

"**The tumor margins exhibited a distinct mutational profile**, with newly identified SNVs diminishing in a distance-dependent manner from the tumor boundary."

2. In the Results, we revised:

“We further investigated the genes associated with SNVs predominantly present in tumor margins and found them to be highly related to the extracellular matrix and cell adhesion processes (Fig. 4G), suggesting that the **mutational stress** in the adjacent margins may help reshape the microenvironment, potentially contributing to tumor immune escape.”
to:
“We further investigated the genes associated with SNVs predominantly present in tumor margins and found them to be highly related to the extracellular matrix and cell adhesion processes (Fig. 4G), suggesting that the **tumor cells** in the adjacent margins may help reshape the microenvironment, potentially contributing to tumor immune escape.”

3. In the Results, we revised:
“We found that the expression of genes related to both pathways declined along with the normalized SNV count in the tumor margins as the distance increased from the tumor boundary (Fig. 4H, Supplementary Fig. 6B), **confirming that the mutational stress in tumor margins contributes to the reshaping of the tumor microenvironment.**”
to:
“We found that the expression of genes related to both pathways declined along with the normalized SNV count in the tumor margins as the distance increased from the tumor boundary (Fig. 4H, Supplementary Fig. 6B). **This suggests that tumor expansion may lead to the emergence of new tumor cells at the margin, which could subsequently give rise to novel SNVs**.”

4. In the Results, we revised:
“The relationship between **the strength of mutational stress in the tumor margins** and tumor progression remains largely unknown.”
to:
“The relationship between **the characteristics of the tumor margin microenvironment** and tumor progression remains largely unknown.”

5. In the Results, we revised:
“Furthermore, we observed different rates of decline in **mutational stress** across tumor samples and found that the rate of decline in mutational stress is highly correlated with the immune activity of the tumor section.”
to:
“Furthermore, we observed varying rates of decline in the **mutational dynamics** at the tumor margins across tumor samples and found that the rate of decline in mutational dynamics is highly correlated with the immune activity of the tumor section.”

6. In the Discussion, we added the following sentence:
“…**We recognized that mutations at the tumor margins may originate from newly proliferating tumor cells, which influence the tumor microenvironment in the peripheral regions**…”

Additionally, to identify which SNVs are associated with tumor margin cells, we included Supplementary Figure 6B and integrated it after Figure 4F:
“…**For instance, the mutation chr5_151661589:G>C is located in the UTR region of *SPARC*, a gene highly expressed in metastatic tumors (PMID: 18849185), potentially indicating the invasiveness of the tumor margin. Similarly, the UTR mutation chr7_23274960:A>T in GPNMB may also influence *GPNMB* expression, thereby promoting tumor metastasis (PMID: 36050467) (Supplementary Figure 6B).** …”

C

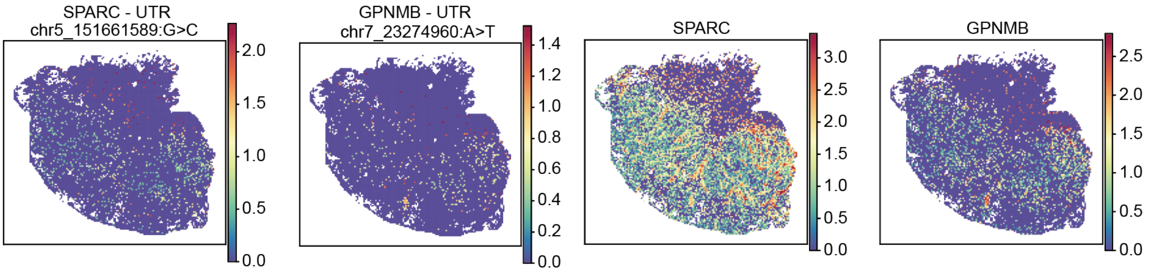

Supplementary Figure 6C: Spatial visualization of *SPARC* and *GPNMB* gene expression and the distribution of corresponding representative SNVs.

3. Hypoxia has also been described in tumor cores, but this study primarily focuses on the tumor boundary. For example, PMID: 34002049 describes these evolutionary mechanisms, is this observed in these datasets?

Reply: We sincerely thank the reviewer for their valuable input. The results of this study (PMID: 34002049) supports our view well. But unfortunately, we do not have access to the raw data of this study. Thus, we cannot check whether hypoxia also occurs at the tumor margin of this dataset. Additionally, another recent study (PMID: 37285434) also showed that significant collagen deposition at the tumor margin increases the density of the extracellular matrix (ECM), thereby reducing oxygen supply and creating a hypoxic environment. We included both papers in the Discussion section as follows:
"… **Although the tumor center in ccRCC has been characterized by proliferation, necrosis, and hypoxia (PMID: 34002049), the tumor margin exhibits significant collagen deposition, leading to increased ECM density and stiffness, which further reduces oxygen supply and creates a hypoxic environment (PMID: 37285434).** …"

Reviewer #2: In "SpatialSNV: A Novel Method for ...", the authors describe a new method for elucidating the spatial patterns of single nucleotide variants as tumors evolve and grow in tissue. It's a very interesting paper that I'm sure many people in the research community will find useful and want to apply to their own data.
- This work reminded me of the paper that was on somatic mutations in healthy eyelids. Do you remember that one? It would be so interesting to see this method applied to healthy eyelid epidermis. But it brings up an interesting thought: in the manuscript there's a fair amount of discussion of a sort of leading edge of mutations putting "pressure" on the adjacent tissue. And while it's true that as a tumor grows in tissue, there are mechanical forces that put pressure on surrounding tissue, I think using that terminology is not quite accurate in this case. Consider eyelids, it doesn't seem quite apt to discuss the somatic clones as putting pressure on the surrounding eyelid tissue. To me it's maybe more of an ecosystem, a collection of clones. I would suggest softening the descriptions of "pressure" from mutations, it just doesn't seem to make sense to me.

Reply: We sincerely thank the reviewer for their insightful comments. We acknowledge that the concept of mutational pressure at the tumor margins may have been overstated. To soft our language, we revised our manuscript to rephrase the mutational pressure at the tumor boundary as the broader mutational landscape of the tumor margin microenvironment. The specific changes we have made include:
1. In the Abstract, we revised:
"**Increased mutational pressure was observed at tumor margins**, with novel SNVs diminishing in a distance-dependent manner from the tumor boundary."
to:
"**The tumor margins exhibited a distinct mutational profile, with newly identified SNVs** diminishing in a distance-dependent manner from the tumor boundary."

2. In the Results, we revised:
"We further investigated the genes associated with SNVs predominantly present in tumor margins and found them to be highly related to the extracellular matrix and cell adhesion processes (Fig. 4G), suggesting that the **mutational stress in the adjacent margins** may help reshape the microenvironment, potentially contributing to tumor immune escape."
to:
"We further investigated the genes associated with SNVs predominantly present in tumor margins and found them to be highly related to the extracellular matrix and cell adhesion processes (Fig. 4G), suggesting that the **tumor cells** in the adjacent margins may help reshape the microenvironment, potentially contributing to tumor immune escape."

3. In the Results, we revised:
"We found that the expression of genes related to both pathways declined along with the normalized SNV count in the tumor margins as the distance increased from the tumor boundary (Fig. 4H, Supplementary Fig.

6B), **confirming that the mutational stress in tumor margins contributes to the reshaping of the tumor microenvironment.**"
to:
"We found that the expression of genes related to both pathways declined along with the normalized SNV count in the tumor margins as the distance increased from the tumor boundary (Fig. 4H, Supplementary Fig. 6B). **This suggests that tumor expansion may lead to the emergence of new tumor cells at the margin, which could subsequently give rise to novel SNVs.**"

4. In the Results, we revised:
"**The relationship between the strength of mutational stress in the tumor margins and tumor progression** remains largely unknown."
to:
"**The relationship between the characteristics of the tumor margin microenvironment and tumor progression** remains largely unknown."

5. In the Results, we revised:
"Furthermore, we observed different rates of decline in **mutational stress** across tumor samples and found that the rate of decline in **mutational stress** is highly correlated with the immune activity of the tumor section."
to:
"Furthermore, we observed varying rates of decline in the **mutational dynamics** at the tumor margins across tumor samples and found that the rate of decline in **mutational dynamics** is highly correlated with the immune activity of the tumor section."

6. In addition, we have further enriched the Discussion :
"… **We recognized that mutations at the tumor margins may originate from newly proliferating tumor cells, which may influence the tumor microenvironment in the peripheral regions. However, it is important to note that somatic mutation frequencies are also high in normal non-blood tissues (PMID:25999502). Therefore, when examining the mutational landscape of the tumor microenvironment, the extent of the influence of tumor cells on this landscape remains unclear.** …"

- Around line 164, where you observe increased numbers of SNVs at the margin.. well, isn't this exactly where the tumor is growing, at the margin, and therefore would have all the previous somatic mutations of the lineage plus new ones? So this seems to be what's expected.

Reply: We sincerely thank the reviewer for their insightful guidance. We acknowledge that the mutations at the tumor margins may reflect the direction of tumor growth, potentially explaining the emergence of new mutations in these regions. To incorporate this perspective, we revised the statement from:
"…We found that the expression of genes related to both pathways declined along with the normalized SNV count in the tumor margins as the distance increased from the tumor boundary (Fig. 4H, Supplementary Fig. 6B), **confirming that the mutational stress in tumor margins contributes to the reshaping of the tumor microenvironment…**"
to:
"…We found that the expression of genes related to both pathways declined along with the normalized SNV count in the tumor margins as the distance increased from the tumor boundary (Fig. 4H, Supplementary Fig. 6B). **This suggests that tumor expansion may lead to the emergence of new tumor cells at the margin, which could subsequently give rise to novel SNVs**…"

- line 179, again, like my previous comment, the mutation are accumulating at the margin as the tumor is growing out... so I don't think the phrase "...a distance-dependent manner of mutational stress ..." makes sense to me. In regards to immune response, it's potentially that the margin has a greater of neoantigens, and the immune systems senses *that*... not some "mutational pressure" which I feel is slightly misleading. Maybe soften the language here, there's a lot we don't know.

Reply: We thank the reviewer for their insightful comments, which have helped us refine our manuscript. We revised and softened the sentence from:

"…Tumors with higher immune activity exhibited a slower decline in **mutational stress**, indicating that **the presence of immune cells may facilitate mutational pressure in the tumor margins**…"
to:
"…Tumors with higher immune activity exhibited a slower decline in **mutational dynamics**, suggesting that **the tumor margin may harbor more neoantigens, leading to the accumulation of immune cells**…"
This change provides a more plausible explanation for the observed phenomenon by linking immune activity to the potential presence of neoantigens in the tumor margin.

- line 220, Regarding immune cells, I think we can't say that a because a SNV was *inferred*, that means any sort of interaction with any particular cell type is happening. Genes often are multifunctional, because a gene is associated with a cell type external to cancer cells, I have trouble imagining how that indicates that a cancer cell (should be the source of the somatic mutation) is interacting with any population of cells, say B cells. They mutation could just as easily affect a process internal to the cell. Are we talking cell surface receptors? Then maybe. But are they transcription factors? Then probably not. Again, my suggestion is to soften the language here.

Reply: We sincerely thank the reviewer for their thoughtful guidance. We acknowledge that some of our original statements may have overinterpreted the data, particularly in inferring interactions between tumor cells and other cells based on SNVs. Initially, we hypothesized that tumor cells might induce more mutations in the tumor microenvironment, but this remains speculative. To address this, we have softened our language as follows:
1. We revised:
"To reveal **how SNVs within the same SNV group can affect each other**, we analyzed the levels of calculated connectivity between SNVs."
to:
"To reveal **the potential connections between SNV groups**, we analyzed the levels of calculated connectivity between SNVs."

2. We revised:
"In SNV Group 2, for example, chr22@328, chr7@232, and chr19@449 were associated with TIMP3, GPNMB, and APOE, respectively, which are marker genes of tumor-associated macrophages (TAMs) (Fig. 5G), **implying that SNV group 2 may be contributing to the interaction of TAMs with the tumor**."
to:
"In SNV Group 2, for example, chr22@328, chr7@232, and chr19@449 were associated with TIMP3, GPNMB, and APOE, respectively, which are marker genes of tumor-associated macrophages (TAMs) (Fig. 5G). **This suggests that SNV Group 2 may reflect the presence of TAMs in the tumor microenvironment and the genomic alterations driven by their strong transcriptional activity**."

3. We revised:
"We also observed B cell and plasma cell marker genes such as JCHAIN and CD27 in SNV Group 1 enriched region (Supplementary Fig. 7G), **suggesting an active role of B cells and plasma cells in tumor metastasis**."
to:
"We also observed B cell and plasma cell marker genes such as JCHAIN and CD27 in SNV Group 1 enriched region (Supplementary Fig. 7G), **suggesting high transcriptional activity of B cells and plasma cells.**"

4. Additionally, we have added the following statement to the Discussion section:
"… **Moreover, the biological significance of SNV Groups may require further investigation. We hypothesize that SNVs appearing in other cells may indicate high transcriptional activity in these cells within the tumor microenvironment, leading to certain genetic mutations.** …"

- line 292, for example, ".. we noticed that specific SNV groups are responsible for EMT..." I would suggest replacing "responsible" with "associated". We don't know they're responsible!

Reply: We sincerely thank the reviewer for pointing out this issue. Indeed, EMT is a complex process, and the original phrasing was not appropriate. In response to the reviewer's comment, we have revised the sentence

"Moreover, we noticed that specific SNV groups are **responsible** for the EMT process of the tumor margin regions"
to
"Moreover, we noticed that specific SNV groups are **associated** with the EMT process of the tumor margin regions.".

- There is a tendency towards making somewhat broad generalizations, but it needs to be remembered, this is actually a very small sample of patients and tumors. These results are certainly very interesting, and should be followed up, but do not qualify as a sort of universal discovery.

Reply: We sincerely thank the reviewer for their constructive suggestion. We fully acknowledge the issue raised and have taken steps to soften the generalized statements in the Results section.
For example:
1. we revised the sentence "We found that the expression of genes related to both pathways declined along with the normalized SNV count in the tumor margins as the distance increased from the tumor boundary (Fig. 4H, Supplementary Fig. 6B), **suggesting** that the mutational stress in tumor margins contributes to the reshaping of the tumor microenvironment." by replacing "confirming" with "suggesting".
2. Additionally, we added the following sentence in the Discussion section to emphasize the need for further validation:
"… **We observed these intriguing phenomena in the collected samples, but the potential role of SNV groups in EMT requires more data to support, as does the role of SNVs at the tumor margin**…"

- On that subject, there are methods only devoted to neoantigen discovery, and it's my understanding that it's actually very difficult to make those predictions accurately. I am concerned that there's no ground truth to compare your results to.. are there?

Reply: We sincerely thank the reviewer for raising this important issue. Indeed, obtaining ground truth data for neoantigens is challenging, which is why our manuscript primarily focuses on potential neoantigens. To address this limitation, we have implemented a screening strategy to enhance the reliability of our predictions: we performed 6-frame translation of the nucleotide sequences upstream and downstream of SNVs to obtain their corresponding proteins and required that these peptides be reported in existing epitope databases (provided they originate from the same gene) and absent in healthy individuals. This approach aligns with many neoantigen discovery pipelines. Our primary goal is to provide potential neoantigen predictions to guide the rational selection of neoantigen targets for experimental validation. Notably, we identified KRAS neoantigens in CRC samples, which have been widely documented in the literature to be genuinely present in colorectal cancer. These results proved the reliability of our method to some extent.
In light of this, we have added the following statement to the discussion:
"…**We provided a neoantigen prediction guidance in this study, but not performed experimental validations. However, the *KRAS* associated neoantigens, which have been widely documented in the literature to be genuinely present in colorectal cancer, could demonstrate the reliability of our methodology to some extent.** …"

- For neoantigen discovery for therapeutics ... we wouldn't want region specific neoantigens, those would be the ones to filter out. I think we would rather have non-regional (i.e. universal) neoantigens. But maybe, by performing the analysis spatially, neoantigens can be discovered that otherwise would be lost in the bulk signal? What do you think?

Reply: We greatly appreciate the reviewer's thoughtful comment. Indeed, in tumor therapy, there is a greater emphasis on identifying non-regional neoantigens rather than focusing solely on neoantigens specific to tumor subtypes. Therefore, in our manuscript, we primarily analyzed potential neoantigens present in the tumor regions. While spatial analysis may not fully recover information lost in bulk sequencing due to the higher coverage of bulk sequencing, its strength lies in the spatial context it provides. For example, in following studies, we could incorporate spatial immune repertoire analysis (such as spatialVDJ, slide-TCR-seq, etc.) with spaitalSNV to identify potential TCR/BCR clones targeting tumor neoantigens. This would further advance the application of spatialSNV.

- I have a question regarding the number of cells in each sequenced spot. 10X says that there's between 1-10 cells per spot, since you created a bin for the stereo-seq I imagine it's similar. How do you think your results are affected by a mixture of cells being present in each spot? And the spot size? Typically people use something like deconvolution to determine what types of cells are present. Is there a trend across platforms, maybe in the number of identified SNVs?

Reply: We appreciate the reviewer's insightful question. We used a square-bin approach to intergrade DNB spots located in the same place to lagger spots for enhanced visualization which is similar to 10x. In essence, we identified SNV from the pseudo-bulk level and then mapped them to spatial context. Thus, the number of identified SNVs is more affected by the sequencing depth, not by spot size or cell composition. But spot size is important in downstream analysis. For example, when calculating SNV Groups, the results may be influenced by spot size (or the number of cells present in spots) because the calculation considers the distances between spots. When the resolution varies, the distances between spots will change. We recommend using 5 times the minimum distance (10x resolution: 100 µm, Stereo-seq bin100 resolution: 50 µm) as a reference. Cell composition could also be valuable in downstream analysis, such as the responsible cell types for specific SNV or SNV group. We would like to further investigate this in following studies.

- Clarification in exactly what data was used --where was it downloaded, and what was the platform-- should be made clearly in the methods and before, maybe early in the results section. Many of the assertions are made in a very general way, but perhaps only apply to a few of the datasets (i.e. slides). It's all publicly available data, right?

Reply: We thank the reviewer for the thoughtful question. To provide further clarity regarding the sample details, we have included more comprehensive information in Supplementary Figure 1A. We also acknowledge that some of our original conclusions were overly generalized, and we have softened the language in manuscript.

A

Sample Name	Platform	Type	Additional Info	PMID
DCIS1	Visium	Ductal carcinoma in situ	ER+/PR-	35314812
DCIS2	Visium	Ductal carcinoma in situ	ER+/PR+	35314812
CRC-P19-T	Stereo-seq	Colorectal cancer	pMMR	38933545
CRC-P59-T_1	Stereo-seq	Colorectal cancer	pMMR	39592630
CRC-P59-T_2	Stereo-seq	Colorectal cancer	pMMR	39592630
CRC-P67-T	Stereo-seq	Colorectal cancer	pMMR	39592630
LC05-M_DU3	Stereo-seq	Intrahepatic cholangiocarcinoma	Stage II	37337030
LC05-T_FD3	Steroe-seq	Intrahepatic cholangiocarcinoma	Stage II	37337030
Slide-RNA	Slide-RNA-seq	Colorectal cancer	Stage IIIB	34912115
Slide-DNA	Slide-DNA-seq	Colorectal cancer	Stage IIIB	34912115

Supplementary Figure 1A: Table presenting the data sources for each sample.

- In figure 1H, there's six samples listed. Then in Fig 1H, there's three... but which DCIS1 sample is that? and which CRC sample is that? Perhaps sample IDs should be added.

Reply: We thank the reviewer for their valuable suggestion and apologize for any confusion caused. In Figure 1F, we focused on examining the stability of SNVs across different samples (from different patients) while controlling for the same platform. In Figure 1H, we aimed to provide a spatial visualization of SNVs overall. To address this issue, we have made the distinction clearer by adding more explicit annotations to Figure 1H and ensuring consistency in sample labeling across all panels in Figure 1. We hope these adjustments resolve the misunderstanding and improve the clarity of our presentation.

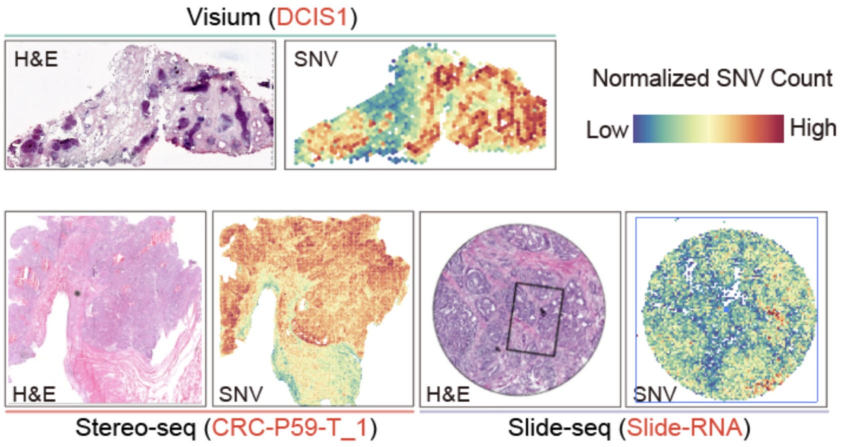

Figure 1H: Hematoxylin and eosin (H&E) staining (left) and spatial visualization(right) of normalized SNV counts across representative samples from various platforms.

- In Fig 1F, it would help if we knew how samples map to platform, maybe color the sample IDs?

Reply: We thank the reviewer for the suggestion. In Figure 1F, to test the accuracy of SNVs identified from spatial transcriptomics, we selected samples from the same platform (Stereo-seq). Additionally, these samples were derived from the same patient or the same cancer type. Therefore, all samples in Figure 1F are from Stereo-seq. For clarity, we colored the sample IDs based on patient information and updated Figure 1F. Additionally, we included all sample information in Supplementary Figure 1A to further enhance clarity.

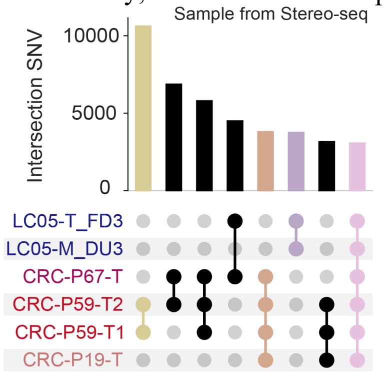

Figure 1F: UpSet plots demonstrating the intersections of effective SNVs among different sections. Yellow and purple bars represent samples from the same patient, while brown and purple bars correspond to samples from liver cancer and colorectal cancer, respectively. Pink bars indicate SNVs that are common in all patients. Sample IDs are colored according to the same patient.

A

Sample Name	Platform	Type	Additional Info	PMID	Sample Name	Platform	Type	Additional Info	PMID
DCIS1	Visium	Ductal carcinoma in situ	ER+/PR-	35314812	CRC-P67-T	Stereo-seq	Colorectal cancer	pMMR	39592630
DCIS2	Visium	Ductal carcinoma in situ	ER+/PR+	35314812	LC05-M_DU3	Stereo-seq	Intrahepatic cholangiocarcinoma	Stage II	37337030
CRC-P19-T	Stereo-seq	Colorectal cancer	pMMR	38933545	LC05-T_FD3	Steroe-seq	Intrahepatic cholangiocarcinoma	Stage II	37337030
CRC-P59-T_1	Stereo-seq	Colorectal cancer	pMMR	39592630	Slide-RNA	Slide-RNA-seq	Colorectal cancer	Stage IIIB	34912115
CRC-P59-T_2	Stereo-seq	Colorectal cancer	pMMR	39592630	Slide-DNA	Slide-DNA-seq	Colorectal cancer	Stage IIIB	34912115

Supplementary Figure 1A: Table presenting the data sources for each sample.

- Is this sequencing -- say for 10X Visium -- similar to single cell sequencing where only the 3' (or 5') end of the gene in sequenced? There's not reads all the way across the exon, so does that limit the SNVs that can be identified?

Reply: We thank the reviewer for raising this important issue. It is indeed noteworthy that the spatial transcriptomics technologies used in this study (Stereo-seq, Visium, and Slide-seq) are all 3' end-based sequencing methods, which may introduce a bias toward detecting SNVs at the 3' end and limit the ability to capture mutations across the entire gene. Recent spatial techniques, such as Patho-DBiT, could provide a relatively even gene body coverage. This experimental advancement is expected to significantly enhance the detection capability of SNVs across exons, providing a more comprehensive mutation landscape.
Therefore, we added the following to the Discussion section:
"…**It is also important to note that platforms such as Visium, Stereo-seq, and Slide-seq are poly(T) capturing-based sequencing methods, which may introduce a strong bias toward SNVs near the 3' end. Recent spatial techniques, such as Patho-DBiT (PMID: 39353436), could provide a relatively even gene body coverage. This experimental advancement may help to improve the efficiency in capturing variations across the gene body**…"

- line 61, "...a specific SNV group was enriched at the tumor margins, and it might enhance the invasive capabilities by regulating the immune evasion progress." how? which group?

Reply: We sincerely thank the reviewer for pointing out this error. In the revised manuscript, we removed this statement and rephrased it as:
"SNV groups may reflect the states of tumor cells and surrounding cells in the microenvironment at the tumor margin."

- line 122 "However, we observed ... displayed different spatial ... patterns.." --- what was the pattern observed and what was compared? DCIS and CRC?

Reply: We thank the reviewer for pointing out this error and apologize for any confusion caused. The term "pattern" refers to the spatial distribution of gene expression (e.g., *GATA3* and *ESR1*) and SNVs. In the DCIS_1 sample, we observed a clear distinction between the distribution of driver genes and SNVs. Specifically, the driver genes exhibited a relatively uniform spatial distribution, while the majority of detected SNVs were concentrated within the tumor regions. This spatial discrepancy led us to conclude that these represent distinct patterns. We hope this explanation helps to clarify our approach and findings.
To prevent ambiguity, we revised:
"However, we observed that tumor driver genes such as *GATA3* and *ESR* in DCIS **displayed different spatial SNV mutation patterns with gene expression**"
to:
"However, we observed that tumor driver genes such as *GATA3* and *ESR* in DCIS **were almost uniformly distributed spatially, but their corresponding SNVs were only present in the tumor. The SNVs and gene expression exhibited different patterns.**"

- line 124, a technicality perhaps, but most statistics depend on the samples being independent, and in spatial data, they are not independent, they are spatially dependent! :-) Don't you think so?

Reply: We sincerely thank the reviewer for raising this important issue. We greatly agree with your perspective. We acknowledge that the statistical method used relies on the assumption of sample independence, while tumorigenesis is closely linked to the spatial distribution of SNVs and gene expression, which may not be entirely independent. We apologize for this oversight and have made efforts to address it. Although we explored alternative methods for validation, we were unable to identify a suitable approach. Therefore, we have softened our language in this section to better reflect the limitations of our analysis. Specifically, we revised:
"Chi-square tests of SNVs and RNA with tumor incidence indicated that the occurrence of SNVs is more closely associated with tumor regions (Fig. 2F). Despite these driver genes being widely expressed across different spatial regions, the SNVs associated with these driver genes were confined solely to the tumor region (Fig. 2G)."
to:
"Chi-square tests of SNVs and RNA with tumor incidence indicated that the occurrence of SNVs is more closely associated with tumor regions (Fig. 2F). **Given the close association of SNVs and genes with tumorigenesis, we further examined the distribution of other driver genes and SNVs to provide additional support for this perspective.** Despite these driver genes being widely expressed across different

spatial regions, the SNVs associated with these driver genes were confined solely to the tumor region (Fig. 2G).”

- line 136, here where you have three subclones, as identified in the source paper, and you perform CNV detection and clustering, and you can reproduce the clonality (right so far?). Can you reproduce the clonality clustering with SNVs? I know you performed some differential SNV analysis across subclones, but it wasn’t clear to me if with just SNVs you could reproduce the clustering?

Reply: We sincerely thank the reviewer for their valuable suggestion. In response, we attempted to use SNVs to reproduce the clustering patterns. However, due to the sparsity of SNVs, the initial results were suboptimal. To address this, we employed an SNV window approach (consistent with the method used for calculating SNV correlations), which successfully resolved the sparsity issue and reproduced the clustering. We have added these results to Supplementary Fig. 5C.
Additionally, we revised the original text:
“We also analyzed the CNVs using the InferCNV algorithm, which confirmed the clonal diversity of the three tumor subclones (Fig. 3B).”
to:
“We also analyzed the CNVs using the InferCNV algorithm, which confirmed the clonal diversity of the three tumor subclones (Fig. 3B). **Additionally, to determine whether SNVs could reproduce the clustering patterns, we performed dimensionality reduction and clustering on the spots using SNV windows as features. This analysis revealed clustering results similar to those observed with CNVs (Supplementary Fig. 5C)**.”

C

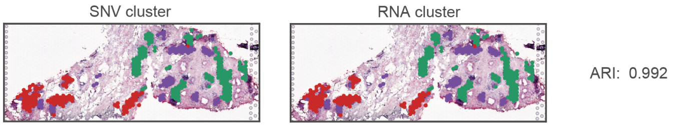

Supplementary Fig. 5C: Spatial visualization of SNV and RNA clustering for tumor subclones.

Minor details
- In "Spots containing fewer than 100-200 genes and genes present in fewer than 325 10 cells were excluded to ensure data quality.", was that indended as "...10 spots..." rather than 10 cells?

Reply: We sincerely thank the reviewer for pointing out this error. We apologize for the incorrect use of terminology in the original text. To ensure data quality, we have revised the sentence as follows:
“Spots containing fewer than 100-200 genes and genes present in fewer than 10 **cells** were excluded to ensure data quality.”
to:
“Spots containing fewer than 100-200 genes and genes present in fewer than 10 **spots** were excluded to ensure data quality.”

- line 35, "Given SNVs occur at the single-nucleotide level ..." well, "SN" stands for single nucleotide, so of course it does. I think this sentence needs a reference and a revision.

Reply: We sincerely thank the reviewer for pointing out this issue. To eliminate redundancy and improve clarity, we have revised the sentence as follows:
“**Given that SNVs occur at the single-nucleotide level, they can emerge more frequently and vary from cell to cell during tumorigenesis.**”
To
“**SNVs accumulate during tumorigenesis and exhibit intercellular heterogeneity (PMID: 34850073)**.”

- line 38 suggested edit: “However, creating a comprehensive map of SNVs with high resolution and precision remains challenging.”

Reply: We sincerely thank the reviewer for pointing out this issue with language usage. To improve clarity and conciseness, we have revised the sentence as follows:
"However, creating a comprehensive map of SNVs with high resolution and precision remains **a big** challenge."
to
"However, creating a comprehensive map of SNVs with high resolution and precision remains challenging."

- line 82: "Most SNVs appeared in only a small number of cells.."... was that a small number of spots?

Reply: We sincerely thank the reviewer for pointing out this issue. Similar to the previous concern, we acknowledge the need for greater precision in our language. To address this, we have revised the sentence as follows:
"Most SNVs appeared in only a small number of cells."
To
"Most SNVs appeared in only a small number of spots."
Additionally, we have carefully reviewed the manuscript to identify and correct similar inaccuracies.
