# Supplementary material for: SpatialSNV: A novel method for identifying and analyzing spatially resolved SNVs in tumor microenvironments
Source: Gigascience. 2025 Jun 14;14:giaf065. doi: 10.1093/gigascience/giaf065 (PMC12166308; doi:10.1093/gigascience/giaf065)
Supplement: giaf065_Authors_Response_To_Reviewer_Comments_original_submission [file giaf065_authors_response_to_reviewer_comments_original_submission.pdf]

Author's Response To Reviewer Comments

Reviewer #1:
1. This study develops a new computational tool using data from other spatial datasets to make inferences about tumor biology. Further clarification of the samples used in the study and how they were selected is needed. Additional details about those samples would also be useful if available (stage, grade, mutation status, metastatic). In figure 1, the samples used for 1D is unclear.

Reply: We thank the reviewer for pointing this out. We revised Figure 1D and incorporated the sample information as shown in the updated figure. Additionally, we included more detailed sample information in Supplementary Figure 1A. We hope these modifications can address the reviewer's concerns and enhance the overall clarity of the manuscript.

Figure 1D: Stacked percentage bar chart showing genomic regions containing effective SNVs. Circular markers denote the platforms of the samples, and square markers specify the cancer types.

Supplementary Figure 1A: Table presenting the data sources for each sample.

2. The study describes increased mutational boundaries at the tumor periphery, but the data to support this limited. Clearer visualization of these SNVs at the periphery would be useful. Which SNVs are associated with these phenotypes? The inferences in the abstract and text are overstated without additional data

Reply: We sincerely thank the reviewer for raising this important issue. We acknowledge that the concept of mutational pressure at the tumor margins may have been overstated due to insufficient data support. To address this, we have revised the Abstract and Results sections to soften our language regarding mutational pressure. The specific changes are as follows:
1. In the Abstract, we revised:
"Increased mutational pressure was observed at tumor margins, with novel SNVs diminishing in a distance-dependent manner from the tumor boundary."
to:
"The tumor margins exhibited a distinct mutational profile, with newly identified SNVs diminishing in a distance-dependent manner from the tumor boundary."

2. In the Results, we revised:
"We further investigated the genes associated with SNVs predominantly present in tumor margins and found them to be highly related to the extracellular matrix and cell adhesion processes (Fig. 4G), suggesting that the mutational stress in the adjacent margins may help reshape the microenvironment, potentially contributing to tumor immune escape."
to:
"We further investigated the genes associated with SNVs predominantly present in tumor margins and found them to be highly related to the extracellular matrix and cell adhesion processes (Fig. 4G), suggesting that the tumor cells in the adjacent margins may help reshape the microenvironment, potentially contributing to tumor immune escape."

3. In the Results, we revised:
"We found that the expression of genes related to both pathways declined along with the normalized SNV count in the tumor margins as the distance increased from the tumor boundary (Fig. 4H, Supplementary Fig. 6B), confirming that the mutational stress in tumor margins contributes to the reshaping of the tumor microenvironment."
to:
"We found that the expression of genes related to both pathways declined along with the normalized SNV count in the tumor margins as the distance increased from the tumor boundary (Fig. 4H, Supplementary

Fig. 6B). This suggests that tumor expansion may lead to the emergence of new tumor cells at the margin, which could subsequently give rise to novel SNVs."

4. In the Results, we revised:
"The relationship between the strength of mutational stress in the tumor margins and tumor progression remains largely unknown."
to:
"The relationship between the characteristics of the tumor margin microenvironment and tumor progression remains largely unknown."

5. In the Results, we revised:
"Furthermore, we observed different rates of decline in mutational stress across tumor samples and found that the rate of decline in mutational stress is highly correlated with the immune activity of the tumor section."
to:
"Furthermore, we observed varying rates of decline in the mutational dynamics at the tumor margins across tumor samples and found that the rate of decline in mutational dynamics is highly correlated with the immune activity of the tumor section."

6. In the Discussion, we added the following sentence:
"…We recognized that mutations at the tumor margins may originate from newly proliferating tumor cells, which influence the tumor microenvironment in the peripheral regions…"

Additionally, to identify which SNVs are associated with tumor margin cells, we included Supplementary Figure 6B and integrated it after Figure 4F:
"…For instance, the mutation chr5_151661589:G>C is located in the UTR region of SPARC, a gene highly expressed in metastatic tumors (PMID: 18849185), potentially indicating the invasiveness of the tumor margin. Similarly, the UTR mutation chr7_23274960:A>T in GPNMB may also influence GPNMB expression, thereby promoting tumor metastasis (PMID: 36050467) (Supplementary Figure 6B). …"

Supplementary Figure 6C: Spatial visualization of SPARC and GPNMB gene expression and the distribution of corresponding representative SNVs.

3. Hypoxia has also been described in tumor cores, but this study primarily focuses on the tumor boundary. For example, PMID: 34002049 describes these evolutionary mechanisms, is this observed in these datasets?

Reply: We sincerely thank the reviewer for their valuable input. The results of this study (PMID: 34002049) supports our view well. But unfortunately, we do not have access to the raw data of this study. Thus, we cannot check whether hypoxia also occurs at the tumor margin of this dataset. Additionally, another recent study (PMID: 37285434) also showed that significant collagen deposition at the tumor margin increases the density of the extracellular matrix (ECM), thereby reducing oxygen supply and creating a hypoxic environment. We included both papers in the Discussion section as follows:
"… Although the tumor center in ccRCC has been characterized by proliferation, necrosis, and hypoxia (PMID: 34002049), the tumor margin exhibits significant collagen deposition, leading to increased ECM density and stiffness, which further reduces oxygen supply and creates a hypoxic environment (PMID: 37285434). …"

Reviewer #2: In "SpatialSNV: A Novel Method for ...", the authors describe a new method for elucidating the spatial patterns of single nucleotide variants as tumors evolve and grow in tissue. It's a very interesting paper that I'm sure many people in the research community will find useful and want to apply to their own data.
- This work reminded me of the paper that was on somatic mutations in healthy eyelids. Do you remember that one? It would be so interesting to see this method applied to healthy eyelid epidermis. But it brings up an interesting thought: in the manuscript there's a fair amount of discussion of a sort of leading edge of mutations putting "pressure" on the adjacent tissue. And while it's true that as a tumor grows in tissue,

there are mechanical forces that put pressure on surrounding tissue, I think using that terminology is not quite accurate in this case. Consider eyelids, it doesn't seem quite apt to discuss the somatic clones as putting pressure on the surrounding eyelid tissue. To me it's maybe more of an ecosystem, a collection of clones. I would suggest softening the descriptions of "pressure" from mutations, it just doesn't seem to make sense to me.

Reply: We sincerely thank the reviewer for their insightful comments. We acknowledge that the concept of mutational pressure at the tumor margins may have been overstated. To soft our language, we revised our manuscript to rephrase the mutational pressure at the tumor boundary as the broader mutational landscape of the tumor margin microenvironment. The specific changes we have made include:
1. In the Abstract, we revised:
"Increased mutational pressure was observed at tumor margins, with novel SNVs diminishing in a distance-dependent manner from the tumor boundary."
to:
"The tumor margins exhibited a distinct mutational profile, with newly identified SNVs diminishing in a distance-dependent manner from the tumor boundary."

2. In the Results, we revised:
"We further investigated the genes associated with SNVs predominantly present in tumor margins and found them to be highly related to the extracellular matrix and cell adhesion processes (Fig. 4G), suggesting that the mutational stress in the adjacent margins may help reshape the microenvironment, potentially contributing to tumor immune escape."
to:
"We further investigated the genes associated with SNVs predominantly present in tumor margins and found them to be highly related to the extracellular matrix and cell adhesion processes (Fig. 4G), suggesting that the tumor cells in the adjacent margins may help reshape the microenvironment, potentially contributing to tumor immune escape."

3. In the Results, we revised:
"We found that the expression of genes related to both pathways declined along with the normalized SNV count in the tumor margins as the distance increased from the tumor boundary (Fig. 4H, Supplementary Fig. 6B), confirming that the mutational stress in tumor margins contributes to the reshaping of the tumor microenvironment."
to:
"We found that the expression of genes related to both pathways declined along with the normalized SNV count in the tumor margins as the distance increased from the tumor boundary (Fig. 4H, Supplementary Fig. 6B). This suggests that tumor expansion may lead to the emergence of new tumor cells at the margin, which could subsequently give rise to novel SNVs."

4. In the Results, we revised:
"The relationship between the strength of mutational stress in the tumor margins and tumor progression remains largely unknown."
to:
"The relationship between the characteristics of the tumor margin microenvironment and tumor progression remains largely unknown."

5. In the Results, we revised:
"Furthermore, we observed different rates of decline in mutational stress across tumor samples and found that the rate of decline in mutational stress is highly correlated with the immune activity of the tumor section."
to:
"Furthermore, we observed varying rates of decline in the mutational dynamics at the tumor margins across tumor samples and found that the rate of decline in mutational dynamics is highly correlated with the immune activity of the tumor section."

6. In addition, we have further enriched the Discussion :
"… We recognized that mutations at the tumor margins may originate from newly proliferating tumor cells,

which may influence the tumor microenvironment in the peripheral regions. However, it is important to note that somatic mutation frequencies are also high in normal non-blood tissues (PMID:25999502). Therefore, when examining the mutational landscape of the tumor microenvironment, the extent of the influence of tumor cells on this landscape remains unclear. …"

- Around line 164, where you observe increased numbers of SNVs at the margin.. well, isn't this exactly where the tumor is growing, at the margin, and therefore would have all the previous somatic mutations of the lineage plus new ones? So this seems to be what's expected.

Reply: We sincerely thank the reviewer for their insightful guidance. We acknowledge that the mutations at the tumor margins may reflect the direction of tumor growth, potentially explaining the emergence of new mutations in these regions. To incorporate this perspective, we revised the statement from:
"…We found that the expression of genes related to both pathways declined along with the normalized SNV count in the tumor margins as the distance increased from the tumor boundary (Fig. 4H, Supplementary Fig. 6B), confirming that the mutational stress in tumor margins contributes to the reshaping of the tumor microenvironment…"
to:
"…We found that the expression of genes related to both pathways declined along with the normalized SNV count in the tumor margins as the distance increased from the tumor boundary (Fig. 4H, Supplementary Fig. 6B). This suggests that tumor expansion may lead to the emergence of new tumor cells at the margin, which could subsequently give rise to novel SNVs…"

- line 179, again, like my previous comment, the mutation are accumulating at the margin as the tumor is growing out... so I don't think the phrase "...a distance-dependent manner of mutational stress ..." makes sense to me. In regards to immune response, it's potentially that the margin has a greater of neoantigens, and the immune systems senses *that*... not some "mutational pressure" which I feel is slightly misleading. Maybe soften the language here, there's a lot we don't know.

Reply: We thank the reviewer for their insightful comments, which have helped us refine our manuscript. We revised and softened the sentence from:
"…Tumors with higher immune activity exhibited a slower decline in mutational stress, indicating that the presence of immune cells may facilitate mutational pressure in the tumor margins…"
to:
"…Tumors with higher immune activity exhibited a slower decline in mutational dynamics, suggesting that the tumor margin may harbor more neoantigens, leading to the accumulation of immune cells…"
This change provides a more plausible explanation for the observed phenomenon by linking immune activity to the potential presence of neoantigens in the tumor margin.

- line 220, Regarding immune cells, I think we can't say that a because a SNV was *inferred*, that means any sort of interaction with any particular cell type is happening. Genes often are multifunctional, because a gene is associated with a cell type external to cancer cells, I have trouble imagining how that indicates that a cancer cell (should be the source of the somatic mutation) is interacting with any population of cells, say B cells. They mutation could just as easily affect a process internal to the cell. Are we talking cell surface receptors? Then maybe. But are they transcription factors? Then probably not. Again, my suggestion is to soften the language here.

Reply: We sincerely thank the reviewer for their thoughtful guidance. We acknowledge that some of our original statements may have overinterpreted the data, particularly in inferring interactions between tumor cells and other cells based on SNVs. Initially, we hypothesized that tumor cells might induce more mutations in the tumor microenvironment, but this remains speculative. To address this, we have softened our language as follows:
1. We revised:
"To reveal how SNVs within the same SNV group can affect each other, we analyzed the levels of calculated connectivity between SNVs."
to:
"To reveal the potential connections between SNV groups, we analyzed the levels of calculated connectivity

between SNVs.”

2. We revised:
“In SNV Group 2, for example, chr22@328, chr7@232, and chr19@449 were associated with TIMP3, GPNMB, and APOE, respectively, which are marker genes of tumor-associated macrophages (TAMs) (Fig. 5G), implying that SNV group 2 may be contributing to the interaction of TAMs with the tumor.”
to:
“In SNV Group 2, for example, chr22@328, chr7@232, and chr19@449 were associated with TIMP3, GPNMB, and APOE, respectively, which are marker genes of tumor-associated macrophages (TAMs) (Fig. 5G). This suggests that SNV Group 2 may reflect the presence of TAMs in the tumor microenvironment and the genomic alterations driven by their strong transcriptional activity.”

3. We revised:
“We also observed B cell and plasma cell marker genes such as JCHAIN and CD27 in SNV Group 1 enriched region (Supplementary Fig. 7G), suggesting an active role of B cells and plasma cells in tumor metastasis.”
to:
“We also observed B cell and plasma cell marker genes such as JCHAIN and CD27 in SNV Group 1 enriched region (Supplementary Fig. 7G), suggesting high transcriptional activity of B cells and plasma cells.”

4. Additionally, we have added the following statement to the Discussion section:
“… Moreover, the biological significance of SNV Groups may require further investigation. We hypothesize that SNVs appearing in other cells may indicate high transcriptional activity in these cells within the tumor microenvironment, leading to certain genetic mutations. …”

- line 292, for example, ".. we noticed that specific SNV groups are responsible for EMT..." I would suggest replacing "responsible" with "associated". We don't know they're responsible!

Reply: We sincerely thank the reviewer for pointing out this issue. Indeed, EMT is a complex process, and the original phrasing was not appropriate. In response to the reviewer’s comment, we have revised the sentence
“Moreover, we noticed that specific SNV groups are responsible for the EMT process of the tumor margin regions”
to
“Moreover, we noticed that specific SNV groups are associated with the EMT process of the tumor margin regions.”.

- There is a tendency towards making somewhat broad generalizations, but it needs to be remembered, this is actually a very small sample of patients and tumors. These results are certainly very interesting, and should be followed up, but do not qualify as a sort of universal discovery.

Reply: We sincerely thank the reviewer for their constructive suggestion. We fully acknowledge the issue raised and have taken steps to soften the generalized statements in the Results section.
For example:
1. we revised the sentence “We found that the expression of genes related to both pathways declined along with the normalized SNV count in the tumor margins as the distance increased from the tumor boundary (Fig. 4H, Supplementary Fig. 6B), suggesting that the mutational stress in tumor margins contributes to the reshaping of the tumor microenvironment.” by replacing “confirming” with “suggesting”.
2. Additionally, we added the following sentence in the Discussion section to emphasize the need for further validation:
“… We observed these intriguing phenomena in the collected samples, but the potential role of SNV groups in EMT requires more data to support, as does the role of SNVs at the tumor margin…”

- On that subject, there are methods only devoted to neoantigen discovery, and it's my understanding that it's actually very difficult to make those predictions accurately. I am concerned that there's no ground truth to compare your results to.. are there?

Reply: We sincerely thank the reviewer for raising this important issue. Indeed, obtaining ground truth

data for neoantigens is challenging, which is why our manuscript primarily focuses on potential neoantigens. To address this limitation, we have implemented a screening strategy to enhance the reliability of our predictions: we performed 6-frame translation of the nucleotide sequences upstream and downstream of SNVs to obtain their corresponding proteins and required that these peptides be reported in existing epitope databases (provided they originate from the same gene) and absent in healthy individuals. This approach aligns with many neoantigen discovery pipelines. Our primary goal is to provide potential neoantigen predictions to guide the rational selection of neoantigen targets for experimental validation. Notably, we identified KRAS neoantigens in CRC samples, which have been widely documented in the literature to be genuinely present in colorectal cancer. These results proved the reliability of our method to some extent.
In light of this, we have added the following statement to the discussion:
"…We provided a neoantigen prediction guidance in this study, but not performed experimental validations. However, the KRAS associated neoantigens, which have been widely documented in the literature to be genuinely present in colorectal cancer, could demonstrate the reliability of our methodology to some extent. …"

- For neoantigen discovery for therapeutics ... we wouldn't want region specific neoantigens, those would be the ones to filter out. I think we would rather have non-regional (i.e. universal) neoantigens. But maybe, by performing the analysis spatially, neoantigens can be discovered that otherwise would be lost in the bulk signal? What do you think?

Reply: We greatly appreciate the reviewer's thoughtful comment. Indeed, in tumor therapy, there is a greater emphasis on identifying non-regional neoantigens rather than focusing solely on neoantigens specific to tumor subtypes. Therefore, in our manuscript, we primarily analyzed potential neoantigens present in the tumor regions. While spatial analysis may not fully recover information lost in bulk sequencing due to the higher coverage of bulk sequencing, its strength lies in the spatial context it provides. For example, in following studies, we could incorporate spatial immune repertoire analysis (such as spatialVDJ, slide-TCR-seq, etc.) with spaitalSNV to identify potential TCR/BCR clones targeting tumor neoantigens. This would further advance the application of spatialSNV.

- I have a question regarding the number of cells in each sequenced spot. 10X says that there's between 1-10 cells per spot, since you created a bin for the stereo-seq I imagine it's similar. How do you think your results are affected by a mixture of cells being present in each spot? And the spot size? Typically people use something like deconvolution to determine what types of cells are present. Is there a trend across platforms, maybe in the number of identified SNVs?

Reply: We appreciate the reviewer's insightful question. We used a square-bin approach to intergrade DNB spots located in the same place to lagger spots for enhanced visualization which is similar to 10x. In essence, we identified SNV from the pseudo-bulk level and then mapped them to spatial context. Thus, the number of identified SNVs is more affected by the sequencing depth, not by spot size or cell composition. But spot size is important in downstream analysis. For example, when calculating SNV Groups, the results may be influenced by spot size (or the number of cells present in spots) because the calculation considers the distances between spots. When the resolution varies, the distances between spots will change. We recommend using 5 times the minimum distance (10x resolution: 100 μm, Stereo-seq bin100 resolution: 50 μm) as a reference. Cell composition could also be valuable in downstream analysis, such as the responsible cell types for specific SNV or SNV group. We would like to further investigate this in following studies.

- Clarification in exactly what data was used --where was it downloaded, and what was the platform-- should be made clearly in the methods and before, maybe early in the results section. Many of the assertions are made in a very general way, but perhaps only apply to a few of the datasets (i.e. slides). It's all publicly available data, right?
Reply: We thank the reviewer for the thoughtful question. To provide further clarity regarding the sample details, we have included more comprehensive information in Supplementary Figure 1A. We also acknowledge that some of our original conclusions were overly generalized, and we have softened the language in manuscript.

Supplementary Figure 1A: Table presenting the data sources for each sample.

- In figure 1H, there's six samples listed. Then in Fig 1H, there's three... but which DCIS1 sample is that? and which CRC sample is that? Perhaps sample IDs should be added.

Reply: We thank the reviewer for their valuable suggestion and apologize for any confusion caused. In Figure 1F, we focused on examining the stability of SNVs across different samples (from different patients) while controlling for the same platform. In Figure 1H, we aimed to provide a spatial visualization of SNVs overall. To address this issue, we have made the distinction clearer by adding more explicit annotations to Figure 1H and ensuring consistency in sample labeling across all panels in Figure 1. We hope these adjustments resolve the misunderstanding and improve the clarity of our presentation.

Figure 1H: Hematoxylin and eosin (H&E) staining (left) and spatial visualization(right) of normalized SNV counts across representative samples from various platforms.

- In Fig 1F, it would help if we knew how samples map to platform, maybe color the sample IDs?
Reply: We thank the reviewer for the suggestion. In Figure 1F, to test the accuracy of SNVs identified from spatial transcriptomics, we selected samples from the same platform (Stereo-seq). Additionally, these samples were derived from the same patient or the same cancer type. Therefore, all samples in Figure 1F are from Stereo-seq. For clarity, we colored the sample IDs based on patient information and updated Figure 1F. Additionally, we included all sample information in Supplementary Figure 1A to further enhance clarity.

Figure 1F: UpSet plots demonstrating the intersections of effective SNVs among different sections. Yellow and purple bars represent samples from the same patient, while brown and purple bars correspond to samples from liver cancer and colorectal cancer, respectively. Pink bars indicate SNVs that are common in all patients. Sample IDs are colored according to the same patient.

Supplementary Figure 1A: Table presenting the data sources for each sample.

- Is this sequencing -- say for 10X Visium -- similar to single cell sequencing where only the 3' (or 5') end of the gene in sequenced? There's not reads all the way across the exon, so does that limit the SNVs that can be identified?

Reply: We thank the reviewer for raising this important issue. It is indeed noteworthy that the spatial transcriptomics technologies used in this study (Stereo-seq, Visium, and Slide-seq) are all 3’ end-based sequencing methods, which may introduce a bias toward detecting SNVs at the 3’ end and limit the ability to capture mutations across the entire gene. Recent spatial techniques, such as Patho-DBiT, could provide a relatively even gene body coverage. This experimental advancement is expected to significantly enhance the detection capability of SNVs across exons, providing a more comprehensive mutation landscape. Therefore, we added the following to the Discussion section:
“…It is also important to note that platforms such as Visium, Stereo-seq, and Slide-seq are poly(T) capturing-based sequencing methods, which may introduce a strong bias toward SNVs near the 3’ end. Recent spatial techniques, such as Patho-DBiT (PMID: 39353436), could provide a relatively even gene body coverage. This experimental advancement may help to improve the efficiency in capturing variations across the gene body…”

- line 61, "...a specific SNV group was enriched at the tumor margins, and it might enhance the invasive capabilities by regulating the immune evasion progress." how? which group?

Reply: We sincerely thank the reviewer for pointing out this error. In the revised manuscript, we removed this statement and rephrased it as:
“SNV groups may reflect the states of tumor cells and surrounding cells in the microenvironment at the tumor margin.”

- line 122 "However, we observed ... displayed different spatial ... patterns.." --- what was the pattern observed and what was compared? DCIS and CRC?

Reply: We thank the reviewer for pointing out this error and apologize for any confusion caused. The term “pattern” refers to the spatial distribution of gene expression (e.g., GATA3 and ESR1) and SNVs. In the DCIS_1 sample, we observed a clear distinction between the distribution of driver genes and SNVs. Specifically, the driver genes exhibited a relatively uniform spatial distribution, while the majority of detected SNVs were concentrated within the tumor regions. This spatial discrepancy led us to conclude that these represent distinct patterns. We hope this explanation helps to clarify our approach and findings.
To prevent ambiguity, we revised:
“However, we observed that tumor driver genes such as GATA3 and ESR in DCIS displayed different spatial SNV mutation patterns with gene expression”
to:
“However, we observed that tumor driver genes such as GATA3 and ESR in DCIS were almost uniformly distributed spatially, but their corresponding SNVs were only present in the tumor. The SNVs and gene expression exhibited different patterns.”

- line 124, a technicality perhaps, but most statistics depend on the samples being independent, and in spatial data, they are not independent, they are spatially dependent! :-) Don't you think so?

Reply: We sincerely thank the reviewer for raising this important issue. We greatly agree with your perspective. We acknowledge that the statistical method used relies on the assumption of sample independence, while tumorigenesis is closely linked to the spatial distribution of SNVs and gene expression, which may not be entirely independent. We apologize for this oversight and have made efforts to address it. Although we explored alternative methods for validation, we were unable to identify a suitable approach. Therefore, we have softened our language in this section to better reflect the limitations of our analysis.
Specifically, we revised:
“Chi-square tests of SNVs and RNA with tumor incidence indicated that the occurrence of SNVs is more closely associated with tumor regions (Fig. 2F). Despite these driver genes being widely expressed across different spatial regions, the SNVs associated with these driver genes were confined solely to the tumor region (Fig. 2G).”
to:
“Chi-square tests of SNVs and RNA with tumor incidence indicated that the occurrence of SNVs is more closely associated with tumor regions (Fig. 2F). Given the close association of SNVs and genes with tumorigenesis, we further examined the distribution of other driver genes and SNVs to provide additional support for this perspective. Despite these driver genes being widely expressed across different spatial regions, the SNVs associated with these driver genes were confined solely to the tumor region (Fig. 2G).”

- line 136, here where you have three subclones, as identified in the source paper, and you perform CNV detection and clustering, and you can reproduce the clonality (right so far?). Can you reproduce the clonality clustering with SNVs? I know you performed some differential SNV analysis across subclones, but it wasn’t clear to me if with just SNVs you could reproduce the clustering?

Reply: We sincerely thank the reviewer for their valuable suggestion. In response, we attempted to use SNVs to reproduce the clustering patterns. However, due to the sparsity of SNVs, the initial results were suboptimal. To address this, we employed an SNV window approach (consistent with the method used for calculating SNV correlations), which successfully resolved the sparsity issue and reproduced the clustering. We have added these results to Supplementary Fig. 5C.
Additionally, we revised the original text:
“We also analyzed the CNVs using the InferCNV algorithm, which confirmed the clonal diversity of the three tumor subclones (Fig. 3B).”
to:
“We also analyzed the CNVs using the InferCNV algorithm, which confirmed the clonal diversity of the three tumor subclones (Fig. 3B). Additionally, to determine whether SNVs could reproduce the clustering patterns, we performed dimensionality reduction and clustering on the spots using SNV windows as features. This analysis revealed clustering results similar to those observed with CNVs (Supplementary Fig.

5C).”

Supplementary Fig. 5C: Spatial visualization of SNV and RNA clustering for tumor subclones.

Minor details
- In "Spots containing fewer than 100-200 genes and genes present in fewer than 325 10 cells were excluded to ensure data quality.", was that indended as "...10 spots..." rather than 10 cells?

Reply: We sincerely thank the reviewer for pointing out this error. We apologize for the incorrect use of terminology in the original text. To ensure data quality, we have revised the sentence as follows:
“Spots containing fewer than 100-200 genes and genes present in fewer than 10 cells were excluded to ensure data quality.”
to:
“Spots containing fewer than 100-200 genes and genes present in fewer than 10 spots were excluded to ensure data quality.”

- line 35, "Given SNVs occur at the single-nucleotide level ..." well, "SN" stands for single nucleotide, so of course it does. I think this sentence needs a reference and a revision.

Reply: We sincerely thank the reviewer for pointing out this issue. To eliminate redundancy and improve clarity, we have revised the sentence as follows:
“Given that SNVs occur at the single-nucleotide level, they can emerge more frequently and vary from cell to cell during tumorigenesis.”
To
“SNVs accumulate during tumorigenesis and exhibit intercellular heterogeneity (PMID: 34850073).”

- line 38 suggested edit: “However, creating a comprehensive map of SNVs with high resolution and precision remains challenging.”

Reply: We sincerely thank the reviewer for pointing out this issue with language usage. To improve clarity and conciseness, we have revised the sentence as follows:
“However, creating a comprehensive map of SNVs with high resolution and precision remains a big challenge.”
to
“However, creating a comprehensive map of SNVs with high resolution and precision remains challenging.”

- line 82: "Most SNVs appeared in only a small number of cells.."... was that a small number of spots?

Reply: We sincerely thank the reviewer for pointing out this issue. Similar to the previous concern, we acknowledge the need for greater precision in our language. To address this, we have revised the sentence as follows:
“Most SNVs appeared in only a small number of cells.”
To
“Most SNVs appeared in only a small number of spots.”
Additionally, we have carefully reviewed the manuscript to identify and correct similar inaccuracies.

Cookie Preference Center

We use cookies which are necessary to make our site work. We may also use additional cookies to analyze, improve and personalize our content and your digital experience. For more information, see our Cookie Policy.

You may choose not to allow some types of cookies. However, blocking some types may impact your experience of our site and the services we are able to offer. See the different category headings below to find out more or change your settings.

Allow all

Manage Consent Preferences

Strictly Necessary Cookies

Always active

These cookies are necessary for the website to function and cannot be switched off in our systems. They are usually only set in response to actions made by you which amount to a request for services, such as setting your privacy preferences, logging in or filling in forms. You can set your browser to block or alert you about these cookies, but some parts of the site will not then work. These cookies do not store any personally identifiable information.

Functional Cookies

These cookies enable the website to provide enhanced functionality and personalisation. They may be set by us or by third party providers whose services we have added to our pages. If you do not allow these cookies then some or all of these services may not function properly.

Performance Cookies

These cookies allow us to count visits and traffic sources so we can measure and improve the performance of our site. They help us to know which pages are the most and least popular and see how visitors move around the site.

Targeting Cookies

These cookies may be set through our site by our advertising partners. They may be used by those companies to build a profile of your interests and show you relevant adverts on other sites. If you do not allow these cookies, you will experience less targeted advertising.

Cookie List

Clear

Apply Cancel
Consent Leg.Interest

Confirm my choices

Powered by onetrust
