# Supplementary material for: SpatialSNV: A novel method for identifying and analyzing spatially resolved SNVs in tumor microenvironments
Source: Gigascience. 2025 Jun 14;14:giaf065. doi: 10.1093/gigascience/giaf065 (PMC12166308; doi:10.1093/gigascience/giaf065)
Supplement: giaf065_Reviewer_1_Report_Original_submission — Lindsay LaFave -- 2/22/2025 [file giaf065_reviewer_1_report_original_submission.pdf]

Reviewer Report

Title: SpatialSNV: A Novel Method for Identifying and Analyzing Spatially Resolved SNVs in Tumor Microenvironments

Version: Original Submission **Date:** 2/22/2025

Reviewer name: Lindsay LaFave

Reviewer Comments to Author:

Liu et al. describes the development of SpatialSNV, an approach that makes use of SNV calling from spatial transcriptomic/genomic data to further describe the role of variants in cancer evolution. In the manuscript the authors make use of published data to develop their tool. Interestingly, they describe patterns for SNV localization at the tumor boundary, which they associate with inflammation programs. They describe the identification of a unique S100A11 variant, as well as other variants, which may contribute to neoantigen load and serve as a potential therapeutic target. The approach is rigorous and has led to some interesting insights, although further clarification of some details and validation would be useful.1. This study develops a new computational tool using data from other spatial datasets to make inferences about tumor biology. Further clarification of the samples used in the study and how they were selected is needed. Additional details about those samples would also be useful if available (stage, grade, mutation status, metastatic). In figure 1, the samples used for 1D is unclear.2. The study describes increased mutational boundaries at the tumor periphery, but the data to support this limited. Clearer visualization of these SNVs at the periphery would be useful. Which SNVs are associated with these phenotypes? The inferences in the abstract and text are overstated without additional data.3. Hypoxia has also been described in tumor cores, but this study primarily focuses on the tumor boundary. For example, PMID: 34002049 describes these evolutionary mechanisms, is this observed in these datasets?

Level of Interest

Please indicate how interesting you found the manuscript: Choose an item.

Quality of Written English

Please indicate the quality of language in the manuscript: Choose an item.

Declaration of Competing Interests

Please complete a declaration of competing interests, considering the following questions:

- Have you in the past five years received reimbursements, fees, funding, or salary from an organisation that may in any way gain or lose financially from the publication of this manuscript, either now or in the future?

- Do you hold any stocks or shares in an organisation that may in any way gain or lose financially from the publication of this manuscript, either now or in the future?
- Do you hold or are you currently applying for any patents relating to the content of the manuscript?
- Have you received reimbursements, fees, funding, or salary from an organization that holds or has applied for patents relating to the content of the manuscript?
- Do you have any other financial competing interests?
- Do you have any non-financial competing interests in relation to this paper?

If you can answer no to all of the above, write 'I declare that I have no competing interests' below. If your reply is yes to any, please give details below.

I declare that I have no competing interests

I agree to the open peer review policy of the journal. I understand that my name will be included on my report to the authors and, if the manuscript is accepted for publication, my named report including any attachments I upload will be posted on the website along with the authors' responses. I agree for my report to be made available under an Open Access Creative Commons CC-BY license (http://creativecommons.org/licenses/by/4.0/). I understand that any comments which I do not wish to be included in my named report can be included as confidential comments to the editors, which will not be published.

Choose an item.

To further support our reviewers, we have joined with Publons, where you can gain additional credit to further highlight your hard work (see: https://publons.com/journal/530/gigascience). On publication of this paper, your review will be automatically added to Publons, you can then choose whether or not to claim your Publons credit. I understand this statement.

Yes Choose an item.
