# Supplementary material for: SpatialSNV: A novel method for identifying and analyzing spatially resolved SNVs in tumor microenvironments
Source: Gigascience. 2025 Jun 14;14:giaf065. doi: 10.1093/gigascience/giaf065 (PMC12166308; doi:10.1093/gigascience/giaf065)
Supplement: giaf065_Reviewer_2_Report_Original_submission — David Gibbs -- 3/16/2025 [file giaf065_reviewer_2_report_original_submission.pdf]

Reviewer Report

Title: SpatialSNV: A Novel Method for Identifying and Analyzing Spatially Resolved SNVs in Tumor Microenvironments

Version: Original Submission **Date:** 3/16/2025

Reviewer name: David Gibbs

Reviewer Comments to Author:

In "SpatialSNV: A Novel Method for ...", the authors describe a new method for elucidating the spatial patterns of single nucleotide variants as tumors evolve and grow in tissue. It's a very interesting paper that I'm sure many people in the research community will find useful and want to apply to their own data.- This work reminded me of the paper that was on somatic mutations in healthy eyelids. Do you remember that one? It would be so interesting to see this method applied to healthy eyelid epidermis. But it brings up an interesting thought: in the manuscript there's a fair amount of discussion of a sort of leading edge of mutations putting "pressure" on the adjacent tissue. And while it's true that as a tumor grows in tissue, there are mechanical forces that put pressure on surrounding tissue, I think using that terminology is not quite accurate in this case. Consider eyelids, it doesn't seem quite apt to discuss the somatic clones as putting pressure on the surrounding eyelid tissue. To me it's maybe more of an ecosystem, a collection of clones. I would suggest softening the descriptions of "pressure" from mutations, it just doesn't seem to make sense to me.- Around line 164, where you observe increased numbers of SNVs at the margin.. well, isn't this exactly where the tumor is growing, at the margin, and therefore would have all the previous somatic mutations of the lineage plus new ones? So this seems to be what's expected.- line 179, again, like my previous comment, the mutation are accumulating at the margin as the tumor is growing out... so I don't think the phrase "...a distance-dependent manner of mutational stress ..." makes sense to me. In regards to immune response, it's potentially that the margin has a greater of neoantigens, and the immune systems senses *that*... not some "mutational pressure" which I feel is slightly misleading. Maybe soften the language here, there's a lot we don't know.- line 220, Regarding immune cells, I think we can't say that a because a SNV was *inferred*, that means any sort of interaction with any particular cell type is happening. Genes often are multifunctional, because a gene is associated with a cell type external to cancer cells, I have trouble imagining how that indicates that a cancer cell (should be the source of the somatic mutation) is interacting with any population of cells, say B cells. They mutation could just as easily affect a process internal to the cell. Are we talking cell surface receptors? Then maybe. But are they transcription factors? Then probably not. Again, my suggestion is to soften the language here.- line 292, for example, ".. we noticed that specific SNV groups are responsible for EMT..." I would suggest replacing "responsible" with "associated". We don't know they're responsible!- There is a tendency towards making somewhat broad generalizations, but it needs to be remembered, this is actually a very small sample of patients and tumors. These results are certainly very interesting, and should be followed up, but do not qualify as a sort of universal discovery.- On that subject, there are methods only devoted to neoantigen discovery, and it's my understanding that it's actually very difficult to make those predictions accurately. I am concerned that there's no

ground truth to compare your results to.. are there?- For neoantigen discovery for therapeutics ... we wouldn't want region specific neoantigens, those would be the ones to filter out. I think we would rather have non-regional (i.e. universal) neoantigens. But maybe, by performing the analysis spatially, neoantigens can be discovered that otherwise would be lost in the bulk signal? What do you think?- I have a question regarding the number of cells in each sequenced spot. 10X says that there's between 1-10 cells per spot, since you created a bin for the stereo-seq I imagine it's similar. How do you think your results are affected by a mixture of cells being present in each spot? And the spot size? Typically people use something like deconvolution to determine what types of cells are present. Is there a trend across platforms, maybe in the number of identified SNVs?- Clarification in exactly what data was used --where was it downloaded, and what was the platform-- should be made clearly in the methods and before, maybe early in the results section. Many of the assertions are made in a very general way, but perhaps only apply to a few of the datasets (i.e. slides). It's all publicly available data, right?- In figure 1H, there's six samples listed. Then in Fig 1H, there's three... but which DCIS1 sample is that? and which CRC sample is that? Perhaps sample IDs should be added.- In Fig 1F, it would help if we knew how samples map to platform, maybe color the sample IDs?- Is this sequencing -- say for 10X Visium -- similar to single cell sequencing where only the 3' (or 5') end of the gene in sequenced? There's not reads all the way across the exon, so does that limit the SNVs that can be identified?- line 61, "...a specific SNV group was enriched at the tumor margins, and it might enhance the invasive capabilities by regulating the immune evasion progress.." how? which group?- line 122 "However, we observed ... displayed different spatial ... patterns.." --- what was the pattern observed and what was compared? DCIS and CRC?- line 124, a technicality perhaps, but most statistics depend on the samples being independent, and in spatial data, they are not independent, they are spatially dependent! :-) Don't you think so?- line 136, here where you have three subclones, as identified in the source paper, and you perform CNV detection and clustering, and you can reproduce the clonality (right so far?). Can you reproduce the clonality clustering with SNVs? I know you performed some differential SNV analysis across subclones, but it wasn't clear to me if with just SNVs you could reproduce the clustering?Minor details- In "Spots containing fewer than 100-200 genes and genes present in fewer than 325 10 cells were excluded to ensure data quality.", was that indended as "...10 spots..." rather than 10 cells?- line 35, "Given SNVs occur at the single-nucleotide level ..." well, "SN" stands for single nucleotide, so of course it does. I think this sentence needs a reference and a revision.- line 38 suggested edit: "However, creating a comprehensive map of SNVs with high resolution and precision remains challenging."- line 82: "Most SNVs appeared in only a small number of cells.."... was that a small number of spots?

Level of Interest

Please indicate how interesting you found the manuscript: Choose an item.

Quality of Written English

Please indicate the quality of language in the manuscript: Choose an item.

Declaration of Competing Interests

Please complete a declaration of competing interests, considering the following questions:

- Have you in the past five years received reimbursements, fees, funding, or salary from an organisation that may in any way gain or lose financially from the publication of this manuscript, either now or in the future?
- Do you hold any stocks or shares in an organisation that may in any way gain or lose financially from the publication of this manuscript, either now or in the future?
- Do you hold or are you currently applying for any patents relating to the content of the manuscript?
- Have you received reimbursements, fees, funding, or salary from an organization that holds or has applied for patents relating to the content of the manuscript?
- Do you have any other financial competing interests?
- Do you have any non-financial competing interests in relation to this paper?

If you can answer no to all of the above, write 'I declare that I have no competing interests' below. If your reply is yes to any, please give details below.

I declare that I have no competing interests

I agree to the open peer review policy of the journal. I understand that my name will be included on my report to the authors and, if the manuscript is accepted for publication, my named report including any attachments I upload will be posted on the website along with the authors' responses. I agree for my report to be made available under an Open Access Creative Commons CC-BY license (http://creativecommons.org/licenses/by/4.0/). I understand that any comments which I do not wish to be included in my named report can be included as confidential comments to the editors, which will not be published.

Choose an item.

To further support our reviewers, we have joined with Publons, where you can gain additional credit to further highlight your hard work (see: https://publons.com/journal/530/gigascience). On publication of this paper, your review will be automatically added to Publons, you can then choose whether or not to claim your Publons credit. I understand this statement.

Yes Choose an item.
